# Signaling Modulation Mediated by Ligand Water Interactions with the Sodium Site at μOR

**DOI:** 10.1021/acscentsci.4c00525

**Published:** 2024-07-17

**Authors:** Rohini S. Ople, Nokomis Ramos-Gonzalez, Qiongyu Li, Briana L. Sobecks, Deniz Aydin, Alexander S. Powers, Abdelfattah Faouzi, Benjamin J. Polacco, Sarah M. Bernhard, Kevin Appourchaux, Sashrik Sribhashyam, Shainnel O. Eans, Bowen A. Tsai, Ron O. Dror, Balazs R. Varga, Haoqing Wang, Ruth Hüttenhain, Jay P. McLaughlin, Susruta Majumdar

**Affiliations:** † Center for Clinical Pharmacology, University of Health Sciences & Pharmacy at St. Louis and Washington University School of Medicine, St. Louis, Missouri 63110, United States; # Department of Anesthesiology and Washington University Pain Center, Washington University School of Medicine, St. Louis, Missouri 63110, United States; ‡ Department of Cellular and Molecular Pharmacology, University of California, San Francisco, San Francisco, California 94158, United States; ∇ Department of Computer Science, Stanford University, Stanford, California 94305, United States; § Department of Structural Biology, Stanford University School of Medicine, Stanford, California 94305, United States; ∥ Department of Pharmacodynamics, University of Florida, Gainesville, Florida 032610, United States; ⊥ Department of Molecular & Cellular Physiology, Stanford University School of Medicine, Stanford, California 94305, United States

## Abstract

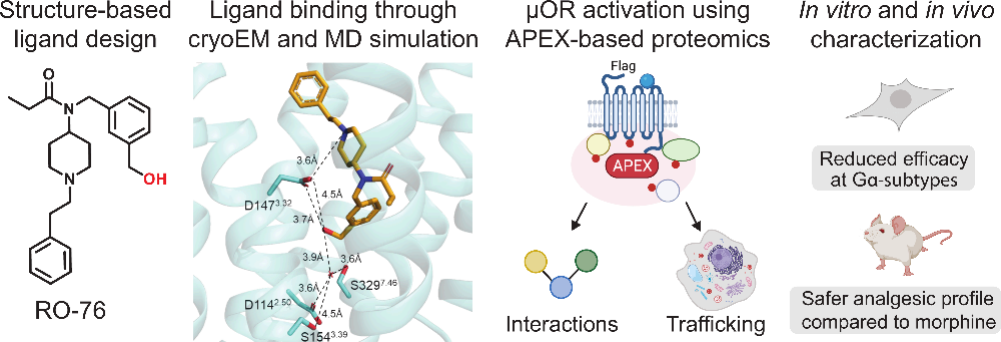

The mu opioid receptor (μOR) is a target for clinically used analgesics. However, adverse effects, such as respiratory depression and physical dependence, necessitate the development of alternative treatments. Recently we reported a novel strategy to design functionally selective opioids by targeting the sodium binding allosteric site in μOR with a supraspinally active analgesic named **C6guano**. Presently, to improve systemic activity of this ligand, we used structure-based design, identifying a new ligand named **RO76** where the flexible alkyl linker and polar guanidine guano group is swapped with a benzyl alcohol, and the sodium site is targeted indirectly through waters. A cryoEM structure of **RO76** bound to the μOR-G_i_ complex confirmed that **RO76** interacts with the sodium site residues through a water molecule, unlike **C6guano** which engages the sodium site directly. Signaling assays coupled with APEX based proximity labeling show binding in the sodium pocket modulates receptor efficacy and trafficking. In mice, **RO76** was systemically active in tail withdrawal assays and showed reduced liabilities compared to those of morphine. In summary, we show that targeting water molecules in the sodium binding pocket may be an avenue to modulate signaling properties of opioids, and which may potentially be extended to other G-protein coupled receptors where this site is conserved.

## Abstract 2

To develop functionally selective opioids, we identified a new ligand named **RO76** in which the sodium site is targeted indirectly through waters.

## Introduction

Agonists targeting the mu opioid receptor (μOR) represent the second line of treatment for neuropathic pain behind antidepressants, SSRIs, and GABA analogs.^1^ However, activation of μOR is also associated with adverse effects like respiratory depression, dependence, tolerance, and constipation.^2,3^ In particular, opioid overdose was responsible for more than 82,820 deaths in 2023 in the US alone.^4^ Thus, developing newer and safer analgesics targeting the μOR is an urgent necessity.

To reduce the side effects associated with μOR activation, various approaches targeting opioid receptors have been reported in the literature. These include peripherally restricted agonists,^5−8^ biased μOR agonists,^9−12^ ligands targeting μOR splice variants,^13^ or opioids with mixed receptor activity for different subtypes.^14−16^ Most of these approaches have targeted the orthosteric binding site except for a few reported allosteric modulators.^17^

Numerous G-protein coupled receptors (GPCRs),^18,19^ including the μOR,^20^ contain a highly conserved sodium allosteric binding site deep in the ligand binding pocket at the center of the 7TM bundle which is surrounded by water molecules.^21,22^

Recently, we reported a “bitopic approach” in which the lead ligand **C6guano** engages a key residue D114^2.50^ in the sodium site through a salt bridge interaction with a guanidine group in the ligand.^23^ While **C6guano** shows μOR-dependent antinociception with reduced adverse effects compared to clinically used opioids, the charged guanidine is highly polar which renders the ligand active only after supraspinal administration.^23^ To optimize the systemic activity of **C6guano** and to increase the blood brain barrier (BBB) penetration, we developed second generation ligands by rigidifying the aliphatic linker group with a phenyl ring and replacing the polar charged guanidine group with neutral groups.

Synthesis and screening of a small library of analogs led to the identification of a lead named **RO76**. This compound displayed a partial agonism profile at all G_α__i/o/z_ subtypes in the TRUPATH assay^24^ and reduced β-arrestin recruitment at both subtypes. To determine the binding pose of this compound, we solved the cryo-EM structure of **RO76** bound to μOR. Snapshots of the μOR-G_i1_-scFv16 complex revealed that **RO76** engages the key sodium site residue through a single water molecule. This finding was further corroborated by molecular dynamics (MD)-simulation studies, which demonstrated the presence of water molecules as mediators in the interaction with sodium site residues. Though water molecules in the sodium binding site are mobile,^18^ some water molecules are stable irrespective of the active or inactive conformational state of GPCRs, as they are engaged in a polar interaction network with G-protein coupled residues.^22^ To the best of our knowledge, these waters have not been rationally targeted before at the μOR or other GPCRs in ligand design.

To systemically characterize receptor activation by **RO76**, we carried out proximity biotin labeling based on an engineered ascorbic acid peroxidase (APEX) coupled with quantitative proteomics.^25^ By capturing snapshots of the μOR proximal proteome at several time points following **RO76** treatment, we demonstrated that, unlike DAMGO, **RO76** does not induce internalization of μOR, consistent with the low β-arrestin-2 recruitment seen in our BRET (bioluminescence resonance energy transfer) assays. Instead, **RO76**-dependent changes in the proximal proteome of μOR are comparable to those of PZM21, a partial G protein-biased μOR agonist.

Finally, in mice, **RO76** was systemically active in the 55 °C warm-water tail withdrawal (WWTW) assay and displayed reduced respiratory depression compared to morphine. When administered chronically, the drug showed far fewer withdrawal symptoms compared to morphine. Withdrawal effects like frequency of jumping, teeth chattering, and forepaw licking were insignificant, whereas rearing frequencies were notably more pronounced in the case of **RO76** as compared to those of saline but less pronounced compared to those of morphine.

Considering the results of *in vitro* cell-based BRET assays, cryo-EM of **RO76** bound to the μOR-G_i1_-scFv16 complex, APEX-based proximity labeling, and behavioral studies in mice, the partial agonist profile of **RO76** appears promising for μOR-dependent analgesia with attenuated side effects.

## Results

### Design and Synthesis of Second-Generation μOR-Bitopics Based on the Fentanyl Scaffold

**Figure 1:**
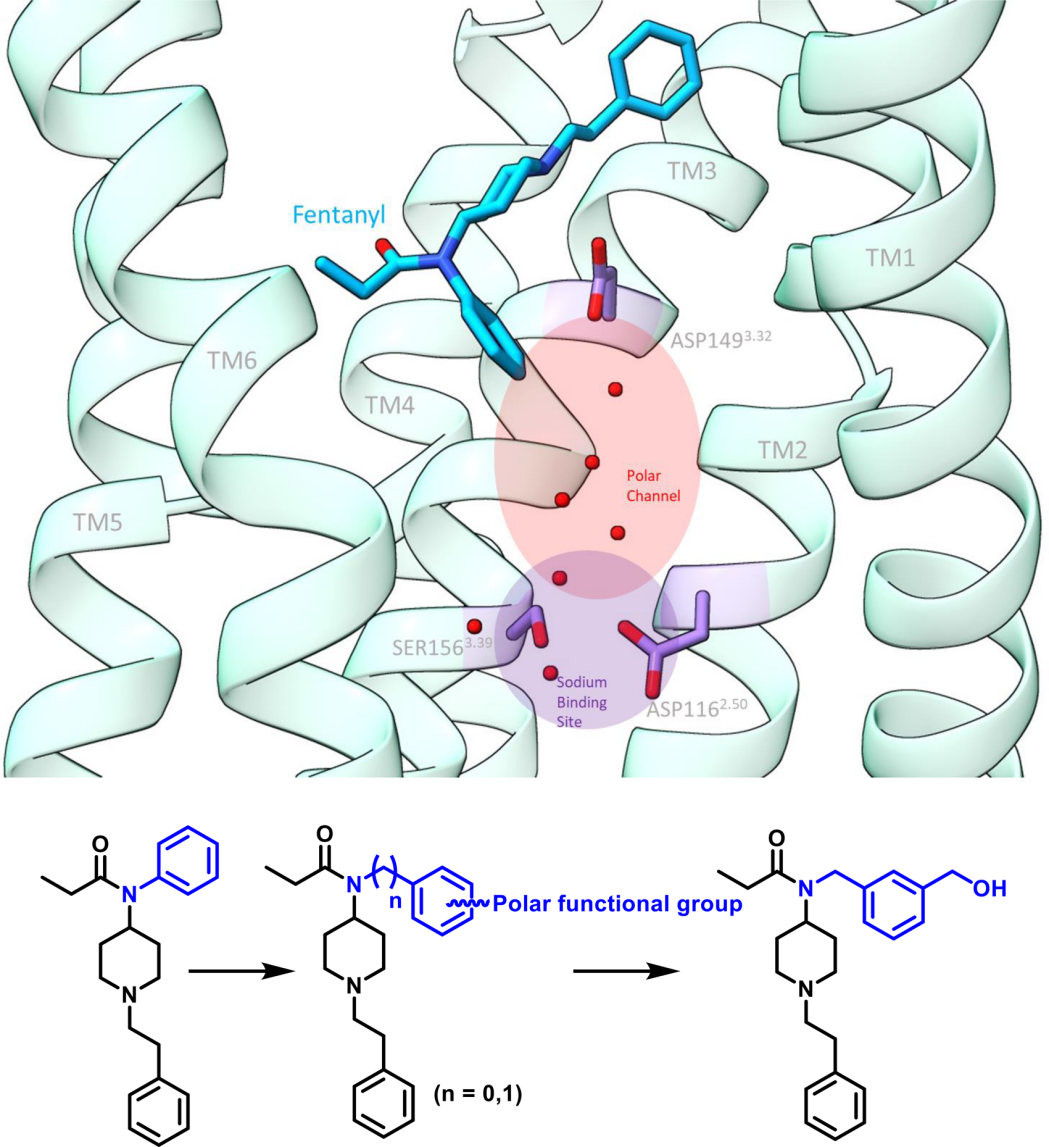
Design of fentanyl analogs targeting the polar cavity of μOR.

**Figure 2:**
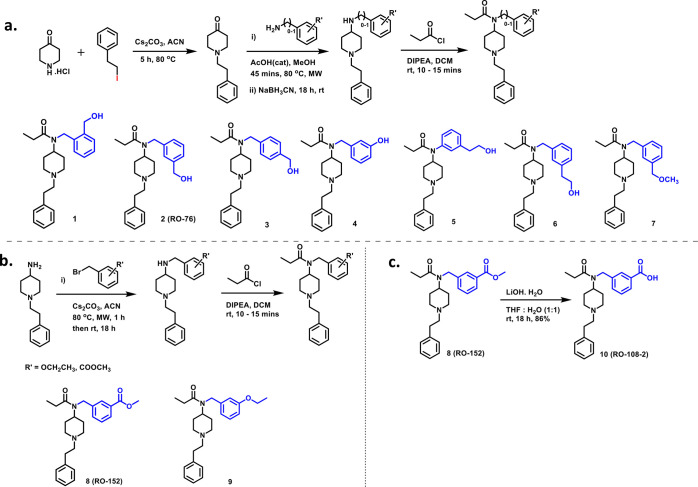
(a–c) General synthetic route for fentanyl analogues.

The design of analogs based on fentanyl templates was aimed to target the water molecules present in the Na^+^ binding site. The phenyl ring linked to the amide nitrogen was modified with polar moieties (Figure 1). We synthesized 10 analogs (Figure 2) and studied their pharmacology using cell based *in vitro* assays (Table 1). The synthesis of these analogues was achieved using a general synthetic method^23^ shown in Figure 2a–c.

**Table 1:**
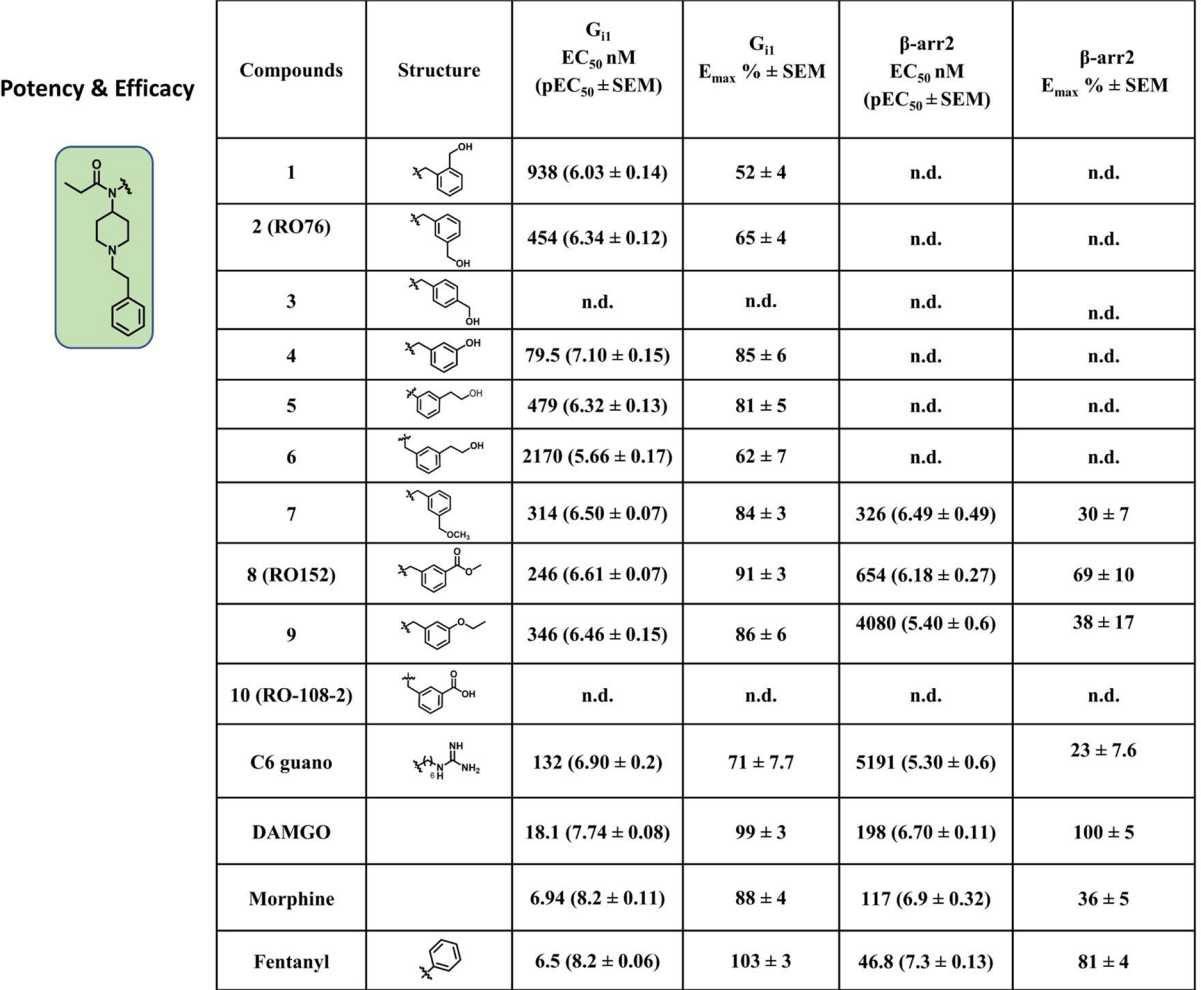
Efficacy and Potency of Fentanyl Analogs at G_i1_-μOR Using G_αβγ_ Biosensors and β-Arrestin-2^a^

a Data were presented as means ± SEM for 2 or 3 biological replicates, where each data set has four technical replicates. The data sets were normalized in reference to the maximum response of DAMGO.

The synthesis began with the alkylation of 4-piperidone monohydrate hydrochloride with (2-iodoethyl) benzene in the presence of cesium carbonate in acetonitrile at 80 °C, resulting in 1-phenethylpiperidin-4-one with 80% yield. In the next step, 1-phenethyl piperidin-4-one underwent reductive amination using sodium cyanoborohydride in the presence of catalytic acetic acid to produce substituted secondary amines. These secondary amines were subsequently acylated using propionyl chloride with DIPEA as a base to furnish fentanyl analogues. Compounds **8** and **9** were synthesized by alkylation of 1-phenethylpiperidin-4-amine with substituted benzyl halides using cesium carbonate as a base followed by propionylation of the secondary amine intermediates (Figure 2b). The putative metabolite of the lead compound **RO76** was synthesized by hydrolyzing an analog with an ester group (Figure 2c). Detailed synthetic procedures are covered in the experimental section of the Supporting Information.

### RO76 Is a μOR-Selective Partial Agonist with Reduced β-Arrestin-1/2 Recruitment

All the synthesized analogues were screened for G-protein (G_i1_) activity and β-arrestin-2 recruitment at the μOR subtype. These activities were evaluated in HEK293T cells using BRET based assays^15,26^ (Table 1). The first set of analogs with a −CH_2_OH group at the ortho, meta, and para positions of the phenyl ring attached to the amide nitrogen of fentanyl template were evaluated. Among the three, the meta substituted analogue **2** (**RO76**) showed higher G_i1_ activity with moderate potency (EC_50_ = 454 nM, *E*_max_ = 65%) compared to the ortho isomer, **1**. The para isomer **3** was inactive in this assay. All three analogs showed no activity in the β-arrestin-2 pathway. We next synthesized a meta substituted phenol derivative, which exhibited 5-fold higher potency over **2** (**RO76**) in G_i1_ assays while retaining the null efficacy in the β-arrestin-2 pathway seen with the former analog. Two other analogs where the −OH group was pushed downward in the receptor were synthesized: **5** with an additional −CH_2_ group between the phenyl ring and −OH, and **6**, with an additional −CH_2_ between the amide nitrogen and phenyl ring of **2** (**RO76**). Evaluation of G_i1_ activity and β-arrestin-2 recruitment revealed that **5** was 5-fold less potent at G_i1_, while **6** retained the G_i1_ potency of **2** and that both **5** and **6** have no detectable β-arrestin-2 recruitment.

Next, we modified the hydroxy group of the 3′-methylphenylmethanol moiety to OCH_3_ leading to **7** displaying similar G_i1_ potency (EC_50_ = 314 nM) and slightly higher efficacy (*E*_max_ = 84%) compared to **RO76**. The drug, however, showed β-arrestin-2 recruitment (EC_50_ = 326 nM, *E*_max_ = 30%). In the next modification, **8**, we replaced the −CH_2_OH arm of 3′-methylphenyl methanol with COOCH_3_, resulting in improved potency (EC_50_ = 246 nM) and efficacy (*E*_max=_ 91%) for G_i1_-μOR as well as robust β-arrestin-2 recruitment (EC_50_ = 654 nM, *E*_max_ = 69%).

Next, we synthesized **9** with 1-ethoxy-3′-methylbenzene, which showed reasonable G_i1_ potency (EC_50_ = 346 nM) and efficacy (*E*_max_ = 86%) for G_i1_-μOR, compared to **RO76**, and displayed β-arrestin-2 recruitment (EC_50_ = 4080 nM, *E*_max_ = 38%). The putative metabolite of **RO76** (discussed later in this manuscript), compound **10** with a 1-carboxy-3-methylbenzene group, also did not exhibit any activity for G_i_ and β-arrestin-2 at μOR.

Overall, the modifications made around the fentanyl scaffold led to the identification of **RO76** as a partial agonist at the G_i1_-μOR with moderate potency. Brief structure–activity relationship studies (SARs) suggested that substituents with a hydrogen donor in the tail region abolished β-arrestin-2 efficacy.

**Figure 3:**
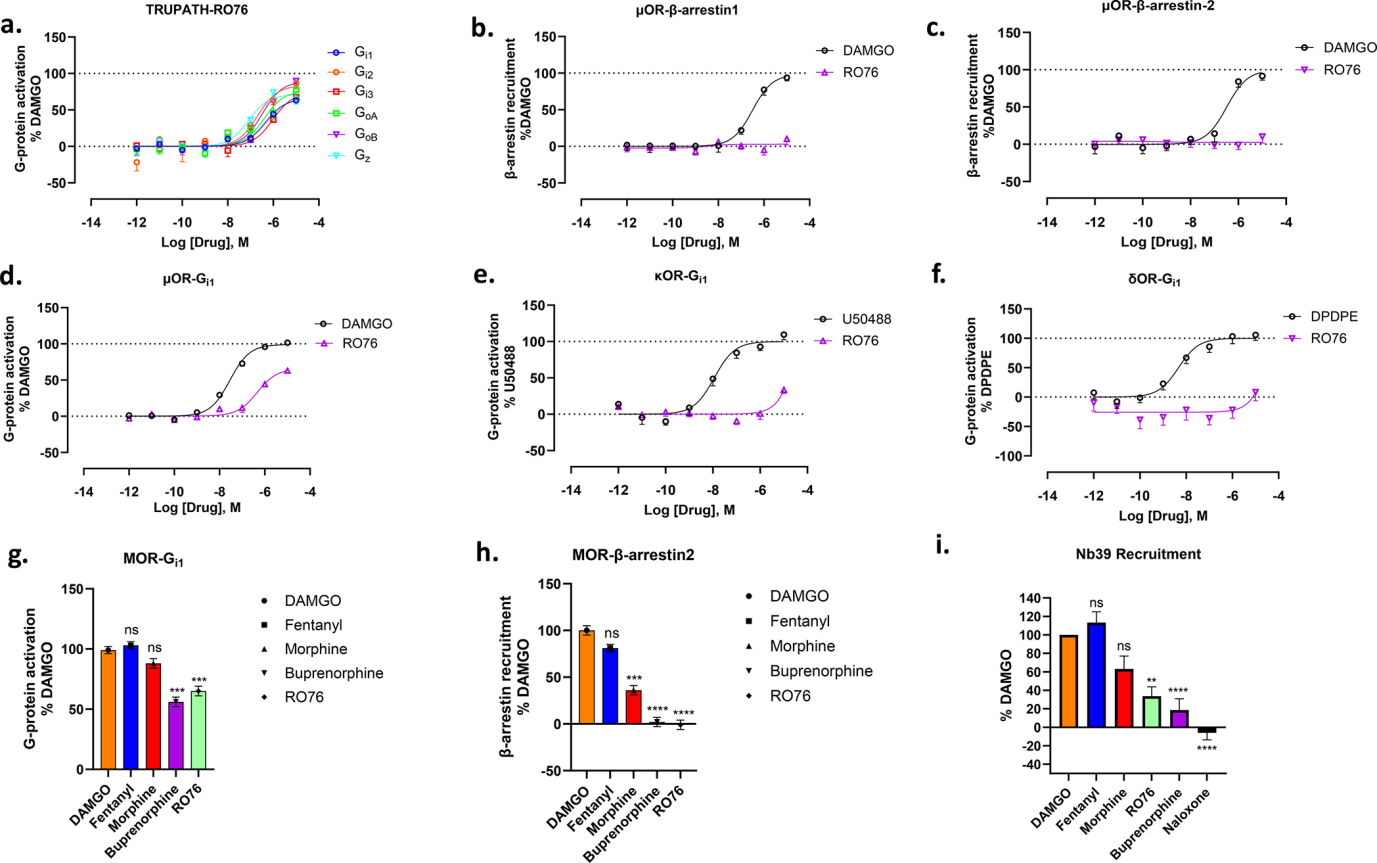
G protein signaling and β-arrestin profiling of **RO76**. Data are presented as means ± SEM for 2 or 3 biological replicates, where each data set has four technical replicates. The data sets were normalized in reference to the maximum response of the prototypic agonist. (a) TRUPATH assay showing G_α_ subtype selectivity profiles with μOR for **RO76**. *E*_max_% ± SEM for G_i1_: 65 ± 5; G_i2_: 84 ± 11; G_i3_: 74 ± 7; G_oA_: 76 ± 9; G_oB_: 89 ± 5; G_*z*_: 71 ± 5. EC_50_ nM (pEC_50_ ± SEM) for G_i1_: 461 (6.34 ± 0.14); G_i2_: 201 (6.7 ± 0.29); G_i3_: 970 (6.01 ± 0.16); G_oA_: 400 (6.4 ± 0.24); G_oB_: 330 (6.48 ± 0.13); G_*z*_: 82.1 (7.09 ± 0.15). (b), (c) **RO76** showed reduced efficacy profiles for μOR β-arrestin-1 and β-arrestin-2 recruitment. (d–f) Efficacy profiles of **RO76** with μOR – G_i1_, κOR – G_i1_, δOR – G_i1_ respectively, representing **RO76** is selective for μOR. *E*_max_% ± SEM for DAMGO: 99 ± 3 and **RO76**: 65 ± 5; EC_50_ nM (pEC_50_ ± SEM) for DAMGO: 28.6 (7.5 ± 0.06) and **RO76**: 461 (6.34 ± 0.14) respectively. (g) G protein signaling of μOR for DAMGO, fentanyl (ns), morphine (ns), buprenorphine (***p = 0.0001), and **RO76** (***p = 0.0002). The data sets were normalized in reference to the maximum response of DAMGO. Data were presented as means ± SEM for 3 or 4 biological replicates. The results were determined by ordinary one-way ANOVA followed by Dunnett’s multiple-comparison test. (h) β-arrestin-2 recruitment profiling of μOR for DAMGO, fentanyl (ns), morphine (***p = 0.0003), buprenorphine (****p < 0.0001), and **RO76** (****p < 0.0001). The data sets were normalized in reference to the maximum response of DAMGO. Data were presented as means ± SEM for 3 or 4 biological replicates. The results were determined by ordinary one-way ANOVA followed by Dunnett’s multiple-comparison test. (i) BRET measurement of recruitment of Nb39 to μOR in the presence of 10 μM DAMGO, fentanyl (ns), morphine (ns), **RO76** (**p < 0.003), buprenorphine (****p < 0.0001), or 10 μM morphinan antagonist Naloxone (****p < 0.0001). Increased recruitment of Nb39 indicates the agonist is shifting the receptor into an active conformation the interaction of Nb39 further stabilizes the conformation. The lower signal for buprenorphine and **RO76** compared to DAMGO and fentanyl is indicative of partial agonism. Data are presented as mean ± SEM for 4 biological replicates where each data set has 4 technical replicates. The results were determined by ordinary one-way ANOVA followed by Dunnett’s multiple-comparison test.

**RO76** was next characterized at other G_αi/o/z_ subtypes (G_i1_, G_i2_, G_i3_, G_oA_, G_oB_, G_Z_) using the TRUPATH assay (Figure 3a). At all G_αi/o/z_ subtypes, **RO76** showed partial agonist activity. β-Arrestin-1 recruitment was tested using a BRET-based assay and, like β-arrestin-2, **RO76** displayed minimal efficacy (Figure 3b–c).

We next investigated the selectivity of **RO76** for μOR, kappa (κOR), and delta opioid receptors (δOR) (Figure 3d–f) based on G_i1_ activation. The data were normalized with respect to their corresponding standard full agonists: DAMGO for μOR, U50,488H for κOR, and DPDPE for δOR. **RO76** showed selectivity for μOR over κOR and δOR, although at a 10 μM concentration, a G_i1_ activation of 33% through κOR and 9% through δOR was generated.

Compared to other μOR ligands such as DAMGO, fentanyl, morphine, and buprenorphine, **RO76** displayed a lower intrinsic G protein efficacy than all ligands except buprenorphine (*E*_max_ = 56% for buprenorphine compared to *E*_max_ = 65% for **RO76**) (Figure 3g). No measurable efficacy was seen for either **RO76** or buprenorphine at β-arrestin-2, while the *E*_max_ for both morphine and fentanyl was higher than that produced by **RO76** (Figure 3h). To evaluate the efficacy of **RO76** accurately in a less amplified assay, we used the nanobody39 (Nb39) recruitment assay^27^ (Figure 3i). Nb39 is recruited to and stabilizes the active confirmation of the receptor.^27^ At 10 μM concentrations, **RO76** showed a lower intrinsic efficacy (*E*_max_ = 30%) compared to morphine (*E*_max_ = 61%), DAMGO (*E*_max_ set at 100%), and fentanyl (*E*_max_ = 123%), while showing a similar efficacy to buprenorphine (*E*_max_ = 23%).

### Cryo-EM Studies of RO76-μOR-G_i1_-scFv16 Complex Revealed an Interaction with Sodium Site Residues Mediated through a Water Molecule

**Figure 4:**
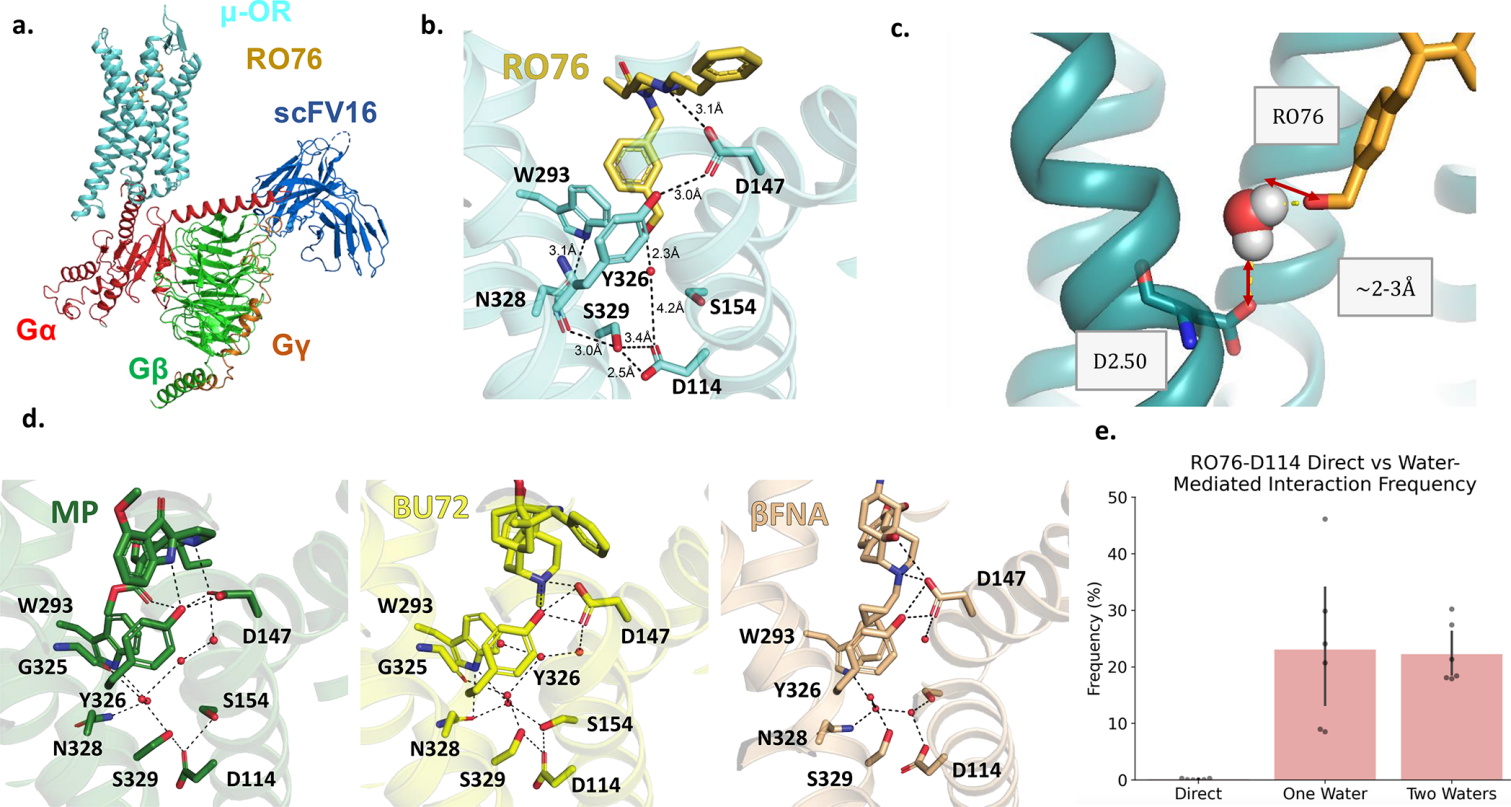
Cryo-EM structure of **RO76** bound to the μOR-G_i1_-scFv16 complex. (a) Cryo-EM structure showing colored G-protein subunits of μOR-G_i1_-scFv16 complex bound to **RO76**. (b) Piperidine nitrogen of **RO76** interacts with orthosteric site through the D147 residue, and the hydroxy group interacts with sodium site residues D114^2.50^, S329^7.46^, Y326^7.43^ through water molecules. (c) MD-Simulation studies showed the existence of water molecules involved in the interaction of the hydroxy group of **RO76** with sodium site residues. (d) Zoom-in view of the receptor revealing the detailed interaction between **RO76**/MP/BU72/β-FNA and orthosteric pocket residues. The receptor is oriented the same way as in panel b. (e) Frequency of direct vs water-mediated interactions between **RO76** and D114^2.50^. Frequency is defined as the fraction of simulation frames in which the hydroxyl of **RO76** was in contact either directly with D114^2.50^, engaged with D114^2.50^ via a single water molecule (excluding frames also containing a direct interaction), or engaged via two consecutive water molecules (excluding frames with direct or single water molecule mediated interactions). Frames involving either no direct and no water mediated interaction or interaction via three or more waters are not shown. Black dots represent the frequency for each individual simulation replicate. Black bars display the standard error of the mean (SEM).

Considering the TRUPATH assay data, which demonstrated that **RO76** has moderate potency in all G_α_-protein subtypes coupling activity, we pursued structural studies to gain further insight into how **RO76** activates μOR. We solved a cryo-EM structure of **RO76** bound to the μOR–G_i1_ complex at 3.2 Å resolution (Figures 4a,b and S1, S2). The **RO76**-bound μOR structure revealed the expected binding pose of the ligand. The fentanyl scaffold overlapped with other fentanyl/fentanyl analog models in the orthosteric pocket, while the benzyl alcohol group projected toward the sodium site (Figure 4b).^23,28,29^ Intriguingly, we observed the density for a water molecule situated between the orthosteric and sodium binding sites (Figure S1-c). This water molecule forms a hydrogen bond with the **RO76** benzyl alcohol oxygen atom, which would suggest a key role in mediating the interaction between **RO76** and sodium site residue D114^2.50^. We also performed MD simulations which confirmed that the interaction between **RO76** and D114^2.50^ is typically water-mediated (Figure 4c,e). Furthermore, the overall conformation of the **RO76**-bound μOR closely mirrors that of other agonists bound to G_i1_-coupled μOR structures, including G protein-biased **C6guano** bitopic ligand, β-arrestin-biased agonist lofentanil, and balanced agonist DAMGO (Figure S1d–f).^23,28,30^ As we previously hypothesized, this conformation is stabilized by interactions with both agonist and a nucleotide-free G_i1_ and so therefore may not truly represent the μOR structure and dynamics modulated solely by the agonist.^31^

To elucidate the detailed interaction between **RO76** and the residues defining the binding pocket, we compared the side chain conformations of **RO76** bound μOR with previously published structures of μOR bound to guano bitopic ligands.^23^ We also considered other high-resolution μOR structures, including the crystal structure of β-funaltrexamine (β-FNA) bound to an inactive receptor (2.8 Å resolution), the crystal structure of bridged pyrrolidinomorphinan (BU72) bound active receptor (2.2 Å resolution), and the cryoEM structure of mitragynine pseudoindoxyl (MP) bound to an active receptor (2.5 Å resolution) (Figure 4d).^32^ Consistent with all μOR structures, D147^3.32^ establishes direct polar contact with the bound ligand, anchoring the binding pose (Figure S1–f). Notably, we detected a rotamer switch of S329^7.46^ and D114^2.50^ in the **RO76** bound receptor structure (Figure 4b). Contrary to the MP- and BU72-bound μOR, the S329^7.46^ side chain orients away from the agonist, breaking the previously observed polar contact network seen in MP and BU72-bound receptors (Figure 4d). Also distinct from the structures of the guano bitopic ligands bound μOR, neither S329^7.46^ nor D114^2.50^ directly engage with the hydrophilic warhead of the bitopic ligand, **RO76** (Figure 4b). We hypothesize that **RO76**’s intermediate length benzyl alcohol group facilitates a unique means of modulating residues in the μOR sodium binding site conferring unique pharmacological properties.

### Confirmation of Water Molecules As Interaction Mediators between Sodium Site Residues and RO76 by Molecular Dynamic Simulation

To gain deeper insight into the binding pose of **RO76** and the mechanism leading to reduced β-arrestin efficacy at μOR we carried out MD simulations, using the **RO76-**bound μOR-G_i1_ complex, which displayed the presence of a water molecule in the sodium binding pocket. MD also revealed water molecules frequently situated between the pendant −CH_2_OH of **RO76** and D114^2.50^ residue of the Na^+^ site, validating our design (Figure 4c,e). The distances between the −OH of the ligand and water as well as between the water and D114^2.50^ fell within the H-bonding distance of 2.5–3 Å (Figure 4c).

**Figure 5:**
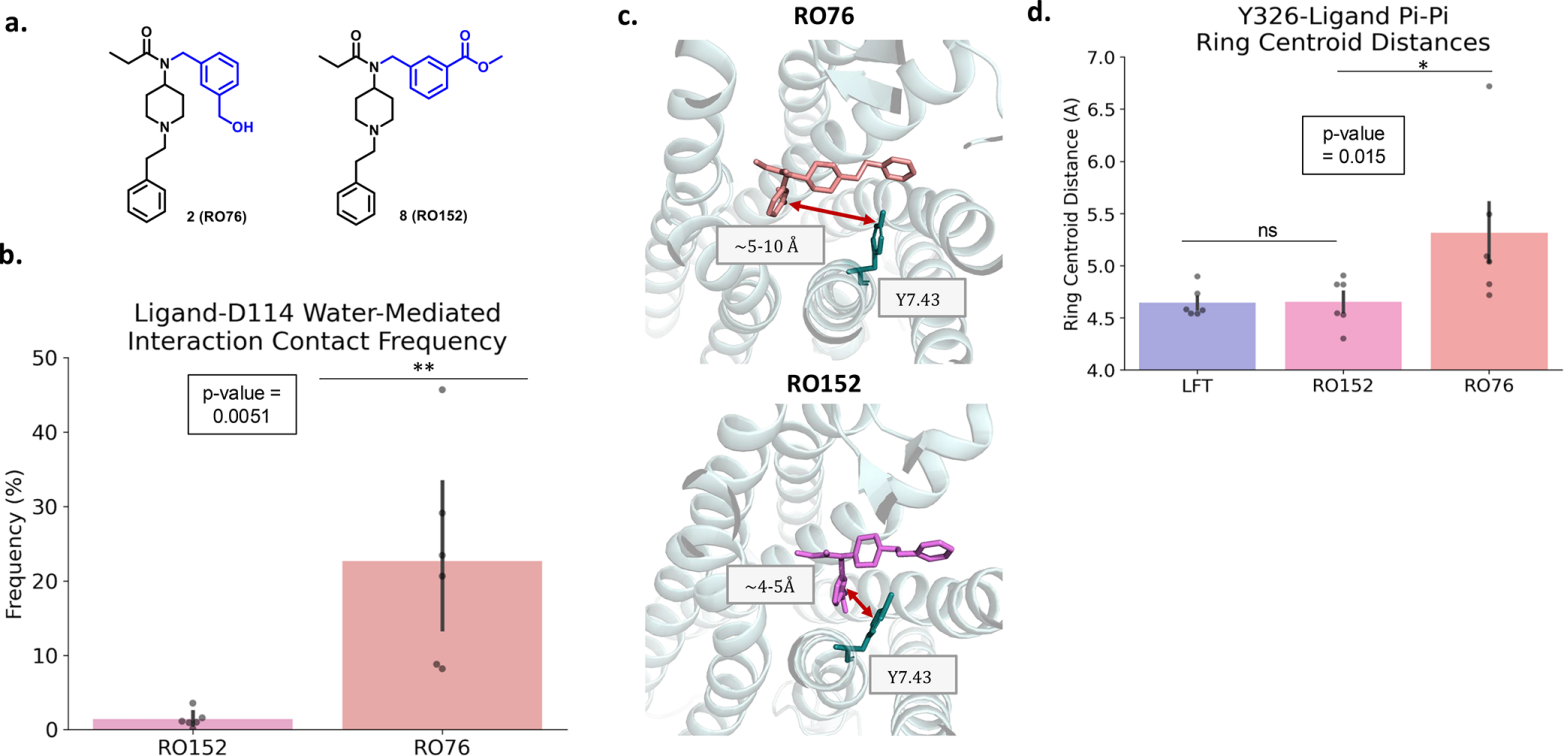
Differences in interaction between the hydrogen bond donor and hydrogen bond acceptor fentanyl derivatives. (a) Chemical structures for **RO76** (hydrogen bond donor) and **RO152** (hydrogen bond acceptor). The phenyl group and altered substituents are shown in blue. (b) Frequency of single water molecule-mediated interaction between ligands and D^2.50^. Black dots represent the frequency for each individual simulation replicate. Black bars display the standard error of the mean (SEM). Significance was calculated using the Mann–Whitney U test (*p* values: * < 0.05, ** < 0.01). (c) Representative MD frames depicting the extent of interaction between the phenyls on Y^7.43^ and ligands **RO76** and **RO152**. Relevant residues are shown in licorice representation. **RO76** is colored salmon and engages in little interaction with Y^7.43^. **RO152** is colored pink and is displayed exhibiting pi-pi stacking with Y^7.43^. (d) Difference in pi-pi stacking frequency between LFT, **RO76**, and **RO152**. **RO152** resembles the β-arrestin-biased LFT molecule in terms of pi-pi stacking, while **RO76** expresses significantly less frequent interactions. Black dots represent the frequency for each individual simulation replicate. Black bars display the standard error of the mean (SEM). Significance was calculated using the Mann–Whitney U test (*p* values: * < 0.05, ** < 0.01).

We also conducted MD on compound **8** (**RO152**) based on the hypothesis that converting a hydrogen bond donor to an acceptor will increase β-arrestin-2 efficacy owing to less engagement of the Na^+^ site through water molecules. Consistent with our SAR observations, we found that **RO152** engages less frequently than **RO76** with the D114^2.50^ residue in the Na^+^ site in our simulations (Figure 5b). The collective evidence from cryoEM and MD simulations suggests that **RO76** indirectly interacts with the Na^+^ site through water molecules.

To understand the mechanism of reduced β-arrestin recruitment we looked at π–π stacking with Y326^7.43^ residue^28,29^ (Figure 5c,d). We have previously shown that the high efficacy agonist lofentanil (LFT) recruits β-arrestin-2 by engaging this residue through π–π stacking through the phenyl ring attached to the amide nitrogen of LFT. Conversely, the low efficacy agonist MP, which does not recruit β-arrestin-2, does not engage Y326^7.43^ (Figure 5c,d). These findings are consistent with reports where Y326^7.43^ has been linked to β-arrestin recruitment and β-arrestin bias in multiple class-A GPCRs.^33−36^

Our findings revealed that the phenyl ring linked to the amide nitrogen of **RO76** is >5 Å away from Y326^7.43^, precluding the formation of a robust π–π stacking interaction with Y326^7.43^ (Figure 5c). In contrast, an analogue of **RO76**, **RO152** can establish a strong π–π stacking interaction with Y326^7.43^, resulting in increased efficacy (*E*_max_ = 69%) at the β-arrestin-2 pathway compared to **RO76** (*E*_max_ = not measurable). Similar to MP and in contrast to LFT, **RO76** showed a lower Y326^7.43^-Q124^2.60^ hydrogen-bond frequency (Figure S4). Our previous work with the μOR demonstrated that this hydrogen bond is prevalent in the “alternative” GPCR conformation, which is associated with β-arrestin binding.^28,33^ Taken together, MD studies suggest that engaging the sodium site or the Y326^7.43^ residue may promote β-arrestin recruitment.

### Proximal Protein Network Mapping of the RO76-Activated μOR Reveals Similarities with the Partial Agonist PZM21

**Figure 6:**
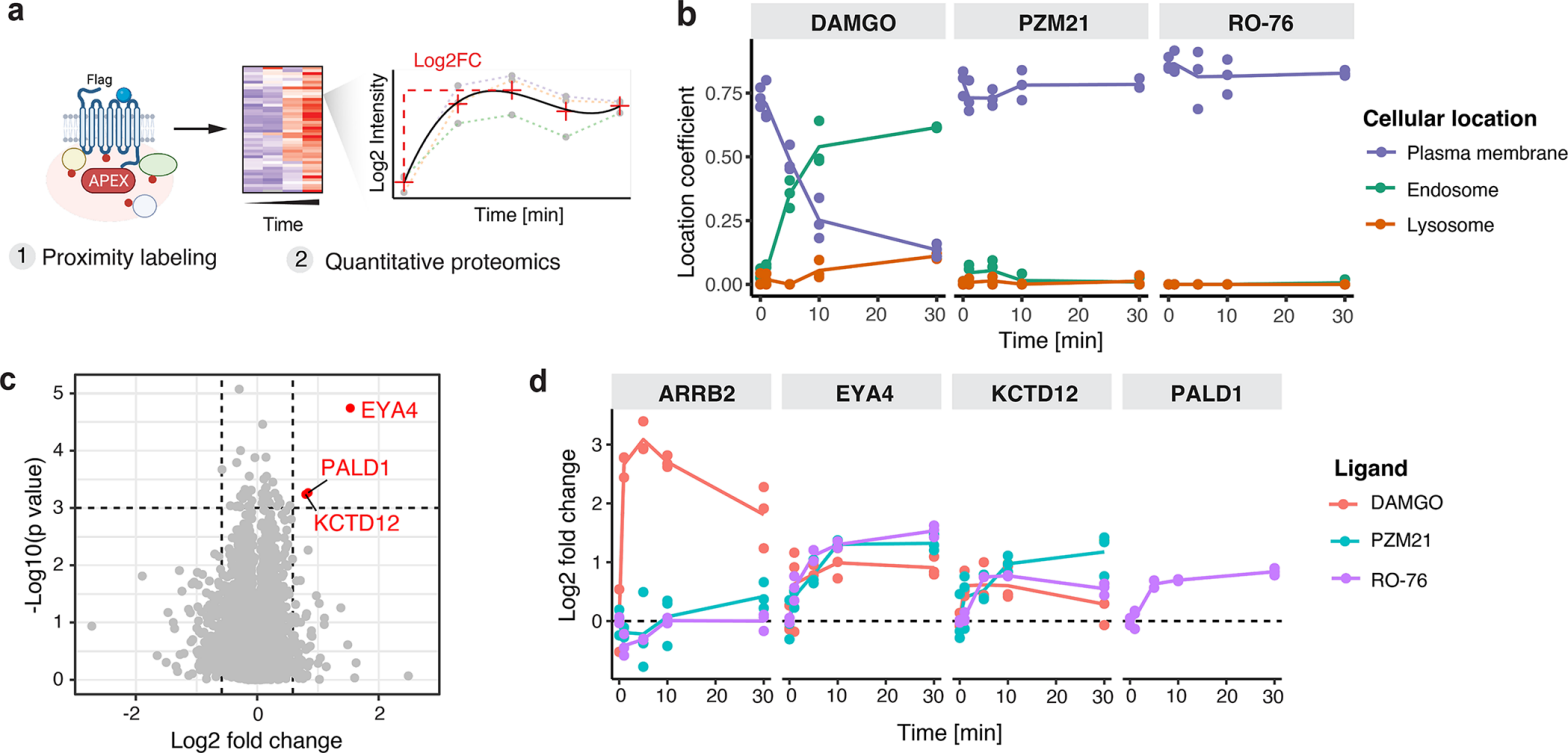
Combining APEX-based proximity labeling and quantitative mass spectrometry for unbiased characterization of RO76-dependent activation of mu-opioid receptor. (a) Schematic of the GPCR-APEX approach taking snapshots of the proximal receptor proteome with minute resolution based on the engineered ascorbic acid peroxidase (APEX) and analyzing the receptor-proximal labeling profile using quantitative mass spectrometry (MS). (b) Determination of location specific coefficients to model receptor trafficking. Intensities of location specific indicator proteins are utilized to calculate coefficients for each location. Location specific coefficients for **RO76** are compared to data for DAMGO and PZM21 from our previous study (Reference: https://www.biorxiv.org/content/10.1101/2022.03.28.486115v1). (c) Volcano plot depicting significantly changing proteins in the proximity of the mu-opioid receptor upon activation with **RO76**. (d) Line charts showing log2 fold change in proximal labeling over the time course of receptor activation for known mu-opioid receptor interactor ARRB2 and significantly changing proteins, EYA4, KCTD12, and PALD1 discovered in this study. Results for **RO76** are overlaid with the results for DAMGO and PZM21 obtained in our previous study (Reference: https://www.biorxiv.org/content/10.1101/2022.03.28.486115v1). For all panels, data represent 3 biological replicates.

We next sought to characterize the μOR activation by **RO76** in an unbiased manner. Recently, we established GPCR-APEX, a proximity labeling method based on fusing the engineered APEX enzyme to the receptor, taking snapshots of its proximal proteome with minute resolution, and analyzing the receptor-proximal labeling profile using quantitative mass spectrometry (MS).^25^ Applying this approach to μOR upon activation with chemically diverse ligands, we were recently able to model ligand-dependent receptor trafficking directly from GPCR-APEX data and identify novel μOR network components regulating receptor signaling and trafficking.^37^ Presently, we extended this approach to examine **RO76**. In brief, to perform proximity labeling, HEK293T cells expressing the μOR-APEX construct were pretreated with biotin-phenol followed by activation of the μOR using **RO76** at a concentration of 10 μM over a time course of 30 min (Figure 6a). At selected time points following receptor activation, proximal biotin labeling was initiated by addition of hydrogen peroxide (H_2_O_2_) for 45 s, followed by cell lysis and enrichment of biotinylated proteins using streptavidin. Finally, we determined relative abundance changes in biotin-labeled proteins after **RO76** stimulation using quantitative proteomics (Figure 6a).

To estimate the fraction of the μOR at a given cellular location across the time course of receptor activation, we utilized a system of spatial APEX references for the plasma membrane, early endosome, and lysosome that were expressed in HEK293T cells and processed in parallel with the μOR-APEX samples. We calculated location coefficients for each time point and biological replicate by solving a linear model based on the protein intensities of location-specific indicator proteins across the spatial references and the μOR-APEX samples (Figure 6b). Notably, over a time course of 30 min the **RO76** activated receptor did not show any detectable receptor internalization and trafficking, which is consistent with the lack of activity for β-arrestin recruitment at μOR (Figure 3b,c). Compared to our previous data for the opioid peptide agonist DAMGO and the “biased” partial agonist PZM21,^37^**RO76** elicited a nearly identical μOR trafficking pattern as PZM21, while activation with DAMGO provoked rapid receptor internalization and trafficking of the μOR (Figure 6b).

To this end, we performed statistical analysis on the protein intensities by fitting the time course with a polynomial curve and performing an F-test to measure the significance of the changes over time. Proteins with significant changes in biotinylation (Log2FC > 0.58 and *p*-value < 0.001) were considered part of the proximal interaction network of the μOR. Based on these criteria, three proteins, EYA4, KCTD12, and PALD1, exhibited significant changes after μOR activation with **RO76** (Figure 6c). These results are comparable to our previous data set for the DAMGO- and PZM21-activated μOR (Figure 6d), in which EYA4 and KCTD12 were discovered with proximal labeling changes for both ligands. We demonstrated in our previous study that (1) EYA4 and KCTD12’s recruitment in the proximity of the μOR is dependent on both receptor and G_i_ activity and (2) EYA4 and KCTD12 impact cellular G_i_ protein responses.^37^ Interestingly, the APEX data for **RO76** revealed the recruitment of putative phosphatase Paladin 1 (PALD1) in the proximal network of activated μOR, a protein that was not detected in our previous study (Figure 6d). While the absence of detection in the previous study does not necessarily imply that PALD1 is specific for **RO76** and might explain **RO76** pharmacology, it would be intriguing to investigate PALD1’s role in μOR signaling in the future.

Taken together, our APEX-based proximity labeling data for **RO76** suggest μOR activation, which induces proximal proteome changes similar to those caused by the G protein-biased partial agonist PZM21, but distinct from the opioid peptide agonist DAMGO: (1) **RO76** does not trigger receptor internalization and trafficking and (2) it induced limited alterations in the proximal interaction network.

### RO76 Shows μOR-Selective Antinociception with Attenuated Adverse Effects

**Figure 7:**
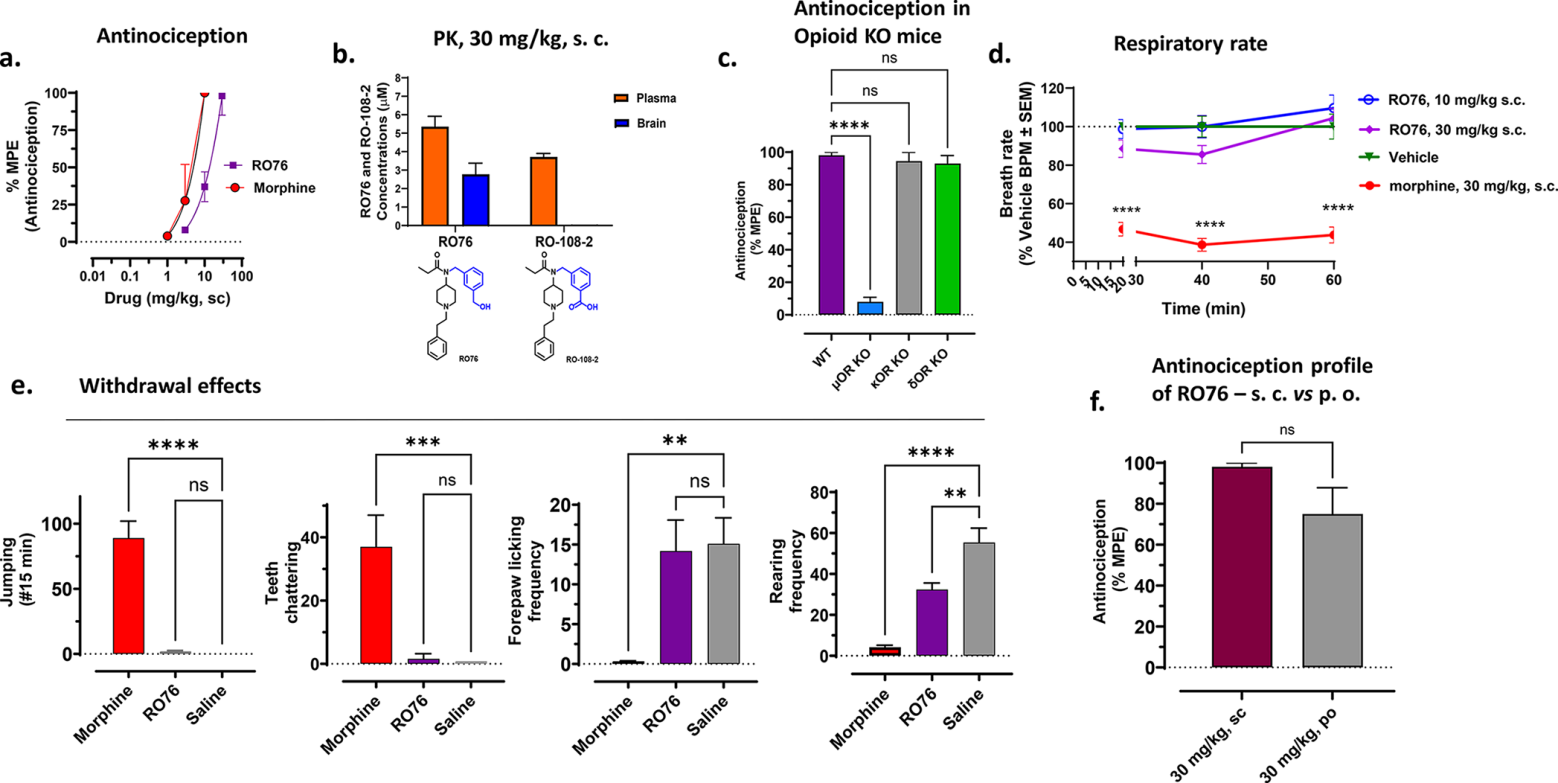
**RO76** exhibits μOR dependent antinociception and attenuated side effects at equianalgesic doses compared to morphine. (a) Antinociception. C57BL/6J (*n* = 8) mice were administered subcutaneously with morphine at doses of 1, 3, 10 mg/kg and **RO76** at doses of 3, 10, and 30 mg/kg; antinociception was measured using the 55 °C tail withdrawal assay. Data are shown as mean % MPE ± SEM. **RO76** showed an antinociception profile similar to morphine. ED_50_ (and 95% C.I.) value at 10 min for **RO76**: 10.54 (8.69–12.91) mg/kg, s.c. (b) Brain and plasma exposure of **RO76**. C57BL/6J mice (*n* = 4) were administered at 30 mg/kg, s.c. dose of **RO76** and brain and plasma were analyzed for intact drug and metabolites at 20 min time point. 5.3 ± 0.56 μM and 2.8 ± 0.59 μM concentrations of **RO76** were found in plasma and brain samples, respectively, using LC-MS/MS. The metabolized – COOH product RO-108–2 was also observed in plasma samples at a 3.7 ± 0.18 μM concentration, whereas in brain samples, metabolized product was not observed. (c) **RO76** antinociception profile in knockout mice. **RO76** (30 mg/kg, s.c.) was administered toC57BL/6J (*n* = 8) WT, MOR KO, KOR KO, and DOR KO mice for antinociceptive effect. The attenuated antinociception was observed prominently in MOR KO mice whereas in DOR KO and KOR KO mice, antinociception was not significantly different from wild-type mice. These results were analyzed with one-way ANOVA and further with Tukey’s posthoc test, F(3,30) = 101.6, *p < 0.0001 relative to WT, ns = *p* > 0.05 relative to WT. Data are shown as mean % MPE ± SEM (d) Respiratory rate. C57BL/6J mice were administered with vehicle (*n* = 12), morphine (30 mg/kg, s.c., *n* = 12) and RO76 (10 mg/kg, sc, *n* = 6 and 30 mg/kg, sc, *n* = 16) and their breath rates were summated every 20 min time point across 120 min. Following subcutaneous drug administration, morphine showed a reduction in breath rate at 20 min (****p = 0.0001), 40 min (****p = 0.0001), and 60 min (****p = 0.0001). **RO76** did not reduce breath rate at any time points. These breath rates were analyzed by 2-way ANOVA followed by Dunnett’s multiple-comparison test. (e) The physical dependence test using “flat dosing” chronic treatment. Saline, morphine (10 mg/kg; s.c.; *n* = 10), **RO76** (30 mg/kg, s.c.; *n* = 10) were administered 2 times a day for 4 days to C57BL/6J mice. On the fifth day, after administration of a final dose, antinociception was checked, followed by Naloxone (10 mg/kg, s.c.) treatment 120 min later, with withdrawal signs measured for 15 min. Bars represents jumping (****p < 0.0001) and teeth chattering frequency (***p = 0.0003) of mice after Naloxone treatment for morphine in first and second graphs whereas for **RO76**, jumping (*p* = 0.9832) and teeth chattering frequencies (*p* = 0.9847) were not significant compared to saline. In third and fourth graph, forepaw licking (*p* = 0.9662) and rearing (**p < 0.0022) frequencies were significant in the case of **RO76** as compared to saline. Morphine showed significant forepaw licking (**p = 0.0025) and rearing (****p < 0.0001) frequencies compared to saline. Statistical significance for these withdrawal effects were determined by 2-way ANOVA followed by Dunnett’s multiple-comparison test. (f) Antinociception profile of **RO76** after subcutaneous vs oral administration. **RO76** was administered to C57BL/6J (*n* = 8) mice at a 30 mg/kg dose by subcutaneous or oral administration. Antinociception (% MPE) observed was 98% via s.c. route, whereas 75% MPE was seen following p.o. administration.

With the partial agonist profile of **RO76** observed across all G_α_-protein subtypes, we conducted further investigations into its *in vivo* pharmacology using mouse models. To assess the antinociceptive effect of **RO76**, male C57BL/6J mice were examined in the 55 °C warm water tail withdrawal assay (WWTW).^26^ Morphine was administered at doses of 1, 3, and 10 mg/kg, while **RO76** was administered at doses of 3, 10, and 30 mg/kg subcutaneously (s.c.) producing dose dependent antinociception in mice (*n* = 8/condition).The estimated ED_50_ (and 95% CI) for **RO76** was 10.18 (8.3–12.6) mg/kg, s.c., which was slightly higher than morphine’s ED_50_ of 4 (8.7–12.9) mg/kg, s.c. (Figure 7a).

Next, an *in vivo* pharmacokinetic study^23,38^ in C57BL/6J mice (*n* = 4) was performed to check the distribution of **RO76** in brain and plasma after subcutaneous administration (30 mg/kg, s.c.) (Figure 7b). After dosing, the brain and plasma samples were collected at the 20 min time point and analyzed for intact drug and metabolite. Liquid chromatography (LC)-MS/MS analysis indicated a **RO76** concentration of 5.3 μM in plasma and 2.8 μM in brain samples.

Notably, a putative metabolite of **RO76** is its acid form after oxidation of the −CH_2_OH group of **RO76** to −COOH (**10,**Table 1). In the plasma samples taken from treated mice, a 3.6 μM concentration of the metabolized −COOH adduct was observed, but the metabolized −COOH adduct was undetected in matching brain samples. However, this is consistent with the polar nature of the −COOH group, which would be expected to prevent the metabolized product from crossing the blood brain barrier. Moreover, the metabolized acid form of **RO76** was found to be inactive at μOR, as determined through an *in vitro* Gi_1_-protein BRET assay (Table 1). Collectively, these data indicate that the antinociceptive effect observed in mice was due to **RO76** and not from the metabolized product.

Subsequently, we examined the dependence of antinociception of **RO76** on μOR *in vivo* using transgenic knockout (KO) mice (Figure 7c). In μOR-KO mice, the antinociceptive effect of **RO76** was significantly attenuated, while in δOR-KO mice and κOR-KO mice, no prominent difference was observed in the antinociception compared to that of wild type (WT) mice (Figure 7c). This marked difference in the antinociceptive profile of μOR-KO indicates that **RO76** antinociception is mediated by μOR. These *in vivo* KO mice experiments results on opioid receptor selectivity of **RO76** align with the *in vitro* Gi BRET assay results.

Furthermore, we evaluated **RO76** for respiratory depression using the Comprehensive Lab Animal Monitoring System (CLAMS).^26^ C57BL/6J mice were administered morphine (30 mg/kg, s.c., *n* = 12) or **RO76** at doses of (10 mg/kg, s.c., *n* = 15) and (30 mg/kg, s.c., *n* = 16) to measure their breath rates at each 20 min interval across 120 min (Figure 7d). While morphine led to a significant decrease in the breath rates (F_(6,118)_ = 2.66, p = 0.02; Two-way RM ANOVA with Tukey’s multiple comparison post hoc test), such effects were not observed at either dose of **RO76**. We also note that at doses higher than 30 mg/kg, sc; **RO76** showed limited motor activity so these doses were not tested for respiration. The exact reasons and the nature of this motor activity are unknown, and will be investigated in the future.

Using chronic “flat dosing” treatment twice a day for 4 days with saline (s.c.), **RO76** (at 30 mg/kg, s.c., n = 10) or morphine (at 10 mg/kg; s.c., *n* = 10), we next examined naloxone-precipitated withdrawal symptoms (Figure 7e). In **RO76**-treated mice, the withdrawal effects of jumping, teeth chattering, and forepaw licking frequencies were not significantly different from saline yet significantly different compared to the responses of morphine-treated mice. Although rearing frequencies were significantly altered after chronic treatment with **RO76** compared to saline, mice that received morphine displayed a significantly greater measured withdrawal effect.

To determine the oral activity of **RO76**, it was administered at 30 mg/kg, p.o. via oral gavage to C57BL/6J (*n* = 8) mice and antinociception tested in the warm water tail withdrawal (WWTW) assay (Figure 7f). The observed antinociception of **RO76** was only slightly lower (75% MPE at 20 min) than that observed with the same dose administered through the subcutaneous route (98% MPE at 20 min). These results suggest that **RO76** has potential for development as an orally active drug.

Collectively, the behavioral studies demonstrate that **RO76** is a μOR-selective agonist displaying similar antinociception to morphine, but with reduced adverse effects of respiratory depression or symptoms of withdrawal denoting reduced physical dependence.

## Discussion

In most class A GPCRs, Na^+^ acts as a negative allosteric modulator (NAM).^18,19^ The Na^+^ site is known to control efficacy,^39−41^ function,^42^ β-arrestin signaling, and basal activity. We have recently reported a bitopic approach using a guanidine group derivatized fentanyl analog to target the Na^+^ site.^23^ Our lead analogue, **C6guano** showed reduced β-arrestin recruitment as well as G_i1_/G_i2_ selectivity and reduced efficacy for G_*z*_ subtype in TRUPATH assays. In mice administered **C6guano** intracerebroventricularly (icv), the drug showed μOR-dependent antinociception in a battery of pain models with reduced CPP in comparison to the prototypic opioid morphine.

To address the blood-brain penetration issues that limit the use of **C6guano**, we used structure-based design to develop the next generation of ligands. In our simulations we found that **C6guano** is unstable with respect to interactions with D114^2.50^, constantly moving up and down within the receptor and migrating toward the orthosteric site. Accordingly, the flexible alkyl chain in **C6guano** was swapped with a phenyl ring, and the charged guanidino group was replaced with a H-bond donor like −OH to engage the polar channel^20^ in which the D114^2.50^ residue resides in the Na^+^ binding pocket.

Screening of ten compounds led to identification of **RO76** with a m-CH_2_OH group as the lead ligand with moderate activity at μOR in G protein assays but with reduced β-arrestin recruitment and no detectable receptor internalization and trafficking, similar to the profile of our first-generation analog. Cryo-EM coupled with MD simulations showed the −CH_2_OH engaging with the D114^2.50^ residue through water molecules. While water molecules have previously been proposed in GPCR structures,^20,22,37,43^ the propensity of stable waters in structures seems limited, according to recent studies by the Dror group.^22^ Efforts across diverse areas of medicinal chemistry have used the strategy of either engaging or displacing structural waters in the binding site for ligand optimization.^44−47^ However, to the best of our knowledge so far, waters have not been rationally exploited in ligand discovery at GPCR’s. SAR studies across the compounds synthesized suggest that a H-bond donor group can engage the water molecules found in the Na^+^ binding pocket. Compounds that engage the Na^+^ site through water molecules displayed reduced β-arrestin recruitment compared to compounds that did not. MD simulations also confirmed that similar to MP^17,28,48^ (a kratom analog which shows reduced β-arrestin recruitment), the phenyl ring attached to the amide nitrogen of **RO76** did not show π–π stacking with Y326^7.43^, while analogs of **RO76** which showed π–π stacking with Y326^7.43^ showed increased β-arrestin recruitment, a pattern further observed with the high efficacy agonist LFT. Taken together, this evidence suggests that the Y326^7.43^ acts as a functional switch which controls β-arrestin recruitment at μOR across diverse templates.

In mice, **RO76** showed optimal brain penetration (brain: plasma ratio = 0.5) compared to **C6guano** and was active when administered by both the subcutaneous and oral routes, reaffirming our design strategy. The *in vivo* potency of **RO76** was surprisingly comparable to morphine although its *in vitro* potency was 65-fold lower than morphine. Other parameters like morphine being a PGP substrate^49^ and possibly more favorable brain:plasma ratios for **RO76** versus morphine^12^ could be at play here. However, the opioid field is replete with compounds showing modest in vitro potency and/or efficacy which prove to be potent analgesics in *in vivo* activity.^50−54^**RO76** did not decrease breath rate at doses of 10 or 30 mg/kg, sc, and in general had reduced respiratory depression and physical dependence after chronic administration as compared to morphine in mice. Thus, targeting the Na^+^ site indirectly through the bitopic approach with water molecules leads to a systemically active and a safer μ opioid antinociceptive agent.

Our approach does have limitations. First, the observed G_α_-selectivity seen with **C6guano** was lost with **RO76**, suggesting that indirect engagement of the Na^+^ site might lead to a loss of G_α_-selectivity. The profile of **RO76** was similar to **C5guano** which was predicted to engage D114^2.50^ through water molecules in our MD simulations.^23^ Although **RO76** is systemically active, it has reduced potency compared with other μOR controls. It was 3-fold, 25-fold, 65-fold, and 69-fold less potent compared to **C6guano**, DAMGO, morphine, and fentanyl, respectively. DAMGO^30^ and morphine^29^ have a different binding mode from fentanyl,^29^ LFT,^28^ and **RO76**. The former two compounds have stabilizing hydrogen bonds that account for their high activity. Fentanyl and LFT do not form hydrogen bonds, but rather form several hydrophobic interactions within the binding pocket. Structures of the μOR bound to fentanyl or LFT reveal a π–π stacking interaction between the phenyl at the base of the binding pocket and residue Y326^7.43^. In simulations, we observed the hydroxymethyl on **RO76** pushing Y326^7.43^ away from the ligand, interrupting the π–π stacking between the ligand phenyl and Y326^7.43^. Therefore, this π–π stacking interaction may contribute to the high potency of fentanyl and LFT, while the reduced π–π stacking frequency in **RO76** explains the lower potency. Further refinement is required to attain a higher G protein activity.

Most importantly, it is not clear why **RO76** shows reduced respiratory depression and physical dependence, suggesting the value of further studies beyond the scope of the current initial testing. In the APEX-based proximity labeling assay,^37^ the proximal proteome changes elicited by **RO76** were similar to PZM21. Notably, the proteomics data for **RO76** also revealed the presence of the putative phosphatase Paladin 1 (PALD1) proximal to the activated μOR. While it remains unclear whether PALD1 is specific for **RO76** compared to other μOR ligands, future investigations into its role in μOR signaling would be intriguing, given PALD1’s demonstrated modulation of signaling through other receptors, including Smoothened^55^ and VEGFR2.^56^

In addition to the similarities observed in the proximity labeling data^37^ between **RO76** and PZM21, PZM21 is reported to have reduced respiratory depression at least by two independent groups,^57,9^ though a dip in respiratory depression is seen at the 15 min time point.^58^ It is possible that the lower intrinsic efficacy seen in Nb39 recruitment assays and TRUPATH assays compared to DAMGO and morphine may be one indicator of **RO76**’s distinct profile in mice. Supporting this, work from Canal,^57,59^ Javitch,^26,60^ and co-workers have proposed low efficacy agonism^61−63^ with an *E*_max_ < buprenorphine as an avenue to separate respiratory depression from antinociception. Further application of the current ligand design strategies may yield ligands useful in testing this theory.

## Conclusion

In summary, by targeting the Na^+^ site through water molecules, we have developed a systemically active bitopic ligand on the fentanyl template. We report that modulation of G protein and β-arrestin-2 pathways can be controlled by utilizing waters in the Na^+^ binding pocket. Given the presence of water molecules in other GPCR^22^ such as δOR,^21^ A_2A_, and β1AR, rationally targeting these might be used to develop pathway selective drugs in those receptors as well.

## Data Availability

The authors declare that all the data supporting the findings of this study are available within the article, extended data, and Supporting Information.

## Supporting Information Available

General synthetic procedures, chemical characterization of compounds, materials and methods for cell based assays, cryo-EM, MD-simulations, mice study and ^1^H and ^13^C NMR spectra of synthesized compounds (PDF)

## Accession Codes

The cryo-EM maps and corresponding coordinates have been deposited in the Electron Microscopy Data Bank (EMDB) under accession codes 44812 (RO76-μOR-G_i1_-scFv16) and the Protein Data Bank (PDB) under accession codes 9BQJ (RO76-μOR-G_i1_-scFv16).
