# Supplementary material for: Signaling Modulation Mediated by Ligand Water Interactions with the Sodium Site at μOR
Source: ACS Cent Sci. 2024 Jul 17;10(8):1490–503. doi: 10.1021/acscentsci.4c00525 (PMC11363324; doi:10.1021/acscentsci.4c00525)
Supplement: Supplementary file 1 — oc4c00525_si_001.pdf [file oc4c00525_si_001.pdf]

Signaling modulation mediated by ligand water interactions with sodium site at μOR

Rohini S. Ople[1], Nokomis Ramos-Gonzalez[1], Qiongyu Li[2], Briana L. Sobecks,[3,5] Deniz Aydin,[3,5] Alexander S. Powers[3,5], Abdelfattah Faouzi,[1] Benjamin J. Polacco[3], Sarah M. Bernhard[1], Kevin Appourchaux[1], Sashrik Sribhashyam[1], Shainnel O. Eans[4], Bowen A.Tsai[4], Ron O. Dror[3,5], Balazs R. Varga[1], Haoqing Wang[5*], Ruth Hüttenhain[5*], Jay P. McLaughlin,[4*] Susruta Majumdar[1*]

[1]Center for Clinical Pharmacology, University of Health Sciences & Pharmacy at St. Louis and Washington University School of Medicine, St. Louis, Missouri 63110, United States; Department of Anesthesiology and Washington University Pain Center, Washington University School of Medicine, St. Louis, Missouri 63110, United States

[2]Department of Cellular and Molecular Pharmacology, University of California, San Francisco, San Francisco CA, 94158, United States

[3]Department of Computer Science, Stanford University, Stanford, CA 94305, United States; Department of Structural Biology, Stanford University School of Medicine, Stanford, CA 94305, United States

[4]Department of Pharmacodynamics, University of Florida, Gainesville, Florida 032610, United States

[5]Department of Molecular & Cellular Physiology, Stanford University School of Medicine, Stanford, CA 94305, United States

*Corresponding authors:

Susruta Majumdar; Email: susrutam@email.wustl.edu; orcid.org/0000- 0002-2931-3823.

Jay P. McLaughlin; Email: jpmclaughlin@ufl.edu; orcid.org/0000-0001-9851-9342.

Haoqing Wang; Email: hwangab@stanford.edu; orcid.org/ 0000-0003-0277-3018.

Ruth Hüttenhain; Email: ruthh@stanford.edu; orcid.org/0000-0002-0896-5910

Table of Contents

1. Chemistry Section: General Experimental......3
1.1. General Synthetic Procedure no. 1 4
1.2. Chemical characterization of compounds prepared from procedure no. 1 5
1.3. General Synthetic Procedures no. 2......8
1.3. Chemical characterization of compounds prepared from procedure no. 2 9
2. Section: Materials and methods 11
2.1. Chemicals and Buffers......11
2.2. BRET based assays (TRUPATH and arrestin signaling) 11
2.3. Nb39 assay 12
2.4. cryo-EM 12
2.5. MD simulations 15
2.6. APEX reaction, biotinylated protein enrichment and preparation for mass spectrometry analysis .17
2.7. Mice study-Invivo assays 19
2.8. Antinociception 20
2.9. Respiratory and locomotor effects......20
3.0. Pharmacokinetic Study......20
3.1. Opioid induced withdrawal effects......21
3. References......21
4. ^{1}H and ^{13}C NMRs......24
5. **Additional supplementary figures and table** 46

Chemistry Section:

General Experimental

All the reagents, starting materials, and solvents were purchased from Sigma-Aldrich Chemicals, Ambeed, Chemscene, Chemimpex and used as such without further purification. Air-sensitive reagents and solutions were transferred to glass apparatus using syringe or cannula via rubber septa. The progress of reactions was monitored using thin-layer chromatography (TLC) with 0.25 mm precoated silica gel plates (60 F254). TLCs were visualized either with UV light or by immersion in an ethanolic solution of phosphomolybdic acid (PMA), para-anisaldehyde, 2,4-DNP, $KMnO_4$, Ninhydrin solution followed by heating with a heat gun for ~15 s. Reaction mixtures were purified by silica flash chromatography on E. Merck 230−400 mesh silica gel 60 using a Teledyne ISCO Combi Flash Rf instrument with UV detection at 280 and 254 nm using 0-20% methanol in DCM solvent system. These compound fractions were further purified on ACCQ Prep HP150 combiflash instrument using acetonitrile containing 0.05% TFA: water containing 0.05% TFA solvent system on REDISEP prep C18 column, 100 Å, 20 x 250 mm. 50 mg of compound was dissolved in 1 to 1.5 ml of MeOH as per compound solubility and the injection volume was 500 µl and the run time was 45 min on a 20% ACN/water to 100%ACN run over 45 min at a flow rate of 18.8 ml/min.

RediSep Rf silica gel normal phase columns were used. The yields reported are isolated yields. All ^1H NMR and ^{13}C NMR spectra were recorded using a Varian 400 or 500 MHz spectrometer at Washington University School of Medicine in St. Louis collected via the Bruker Topspin Software (Bruker Topspin 3.5 pI 6). Coupling constants were measured in Hertz. NMR spectra were processed with Mestre Nova software (ver. 10.0.2.). Chemical shifts are reported in parts per million (ppm) relative to residual solvent peaks rounded to the nearest 0.01 for proton and 0.1 for carbon ($CDCl_3$ ^1H: 7.26, ^{13}C: 77.16). The following abbreviations were used to explain multiplicities: s = singlet, d = doublet, t = triplet, q = quartet, m = multiplet, br = broad. Accurate masses are reported for the molecular ion $[M + H]^+$. High-resolution mass spectra were recorded using positive-ion mode electrospray ionization with an Apollo II ion source on a Bruker 10 Tesla APEX -Q exactive FTICR-MS. Chemical nomenclature was generated using Chem Bio Draw Ultra 13.0

General synthetic procedure for the reductive amination followed by propionylation (procedure no. 1)

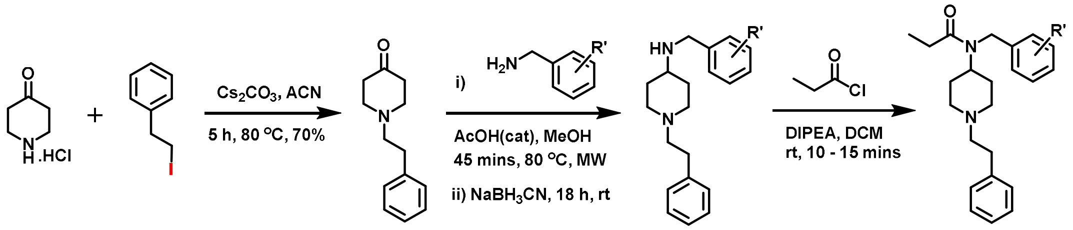

N-phenylethylpiperidin-4-one

4-piperidone monohydrate hydrochloride (3.6 g, 23.4 mmol, 1 equiv.) was dissolved in acetonitrile (20 mL) in a 100 mL round bottom flask equipped with a stir bar. To this mixture, cesium carbonate (15.2 g, 46.8 mmol, 2 equiv.) and (2- iodoethyl) benzene (3.05 mL, 4.88 g, 21.06 mmol) were added at room temperature. The reaction mixture was refluxed at 80 °C for 18 h. After 18 h, the mixture was cooled to room temperature, extracted with DCM (15 mL x 3), washed with saturated $NaHCO_3$ (15 mL) and brine (15 mL). The organic layer was dried over Na_2SO_4 and concentrated in vacuo, purified by flash column chromatography using hexane: ethyl acetate (0 to 100%)[1,2]

^{1}H NMR (400 MHz, chloroform-d): δ 7.33 - 7.27 (m, 2 H), 7.22 (d, J = 6.6 Hz, 3 H), 2.88 - 2.81 (m, 6 H), 2.76 - 2.73 (m, 1 H), 2.73 - 2.70 (m, 1 H), 2.48 (t, J = 6.0 Hz, 4 H)

General procedure for reductive amination followed by propionylation

N-phenylethylpiperidin-4-one (0.983 mmol) was dissolved in 5 mL methanol dried over 4 Å MS in 10 mL microwave vial equipped with a stir bar. To this, amine (0.983 mmol) was added followed by addition of 2-3 drops of acetic acid (cat.) The microwave vial was sealed and heated in microwave reactor at 80 °C for 45 minutes. After 45 minutes of microwave, sodium cyanoborohydride (1.96 mmol) was added slowly to the reaction mixture at ambient temperature and the reaction stirred for 18 h at ambient temperature. The formation of desired product was confirmed by TLC and LCMS analysis. The reaction mixture was filtered through a celite pad and washed with 20% MeOH: DCM. The solution was evaporated in vacuo. The intermediate secondary amine was forwarded to the next step without any purification. Next the intermediate amine was dissolved in dry DCM (5 mL) and to it DIPEA (1.96 mmol) was added followed by propionyl chloride (0.885 mmol). The reaction mixture was stirred at room temperature for 10 to 15 mins and quenched with ice water after completion of the reaction. The reaction mixture was extracted with 10% MeOH: DCM (10 mL x 3), washed with saturated $NaHCO_3$ (10 mL), brine (10 mL) and dried over Na_2SO_4. The organic layer was evaporated in vacuo and purified by combi flash chromatography (DCM/Methanol – 0 to 20%) to obtain RO76

and analogues. These compounds were further purified using reverse phase column chromatography (solvent system - acetonitrile with 0.05%TFA: H_2O with 0.05%TFA) with 45 min of runtime. The final compounds were isolated as a TFA salt.

Characterization of compounds synthesized by reductive amination followed by propionylation (procedure no. 1)

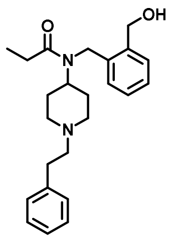

N-(2-(hydroxymethyl)benzyl)-N-(1-phenethylpiperidin-4-yl)propionamide (1)

Yield: 40%

^{1}H NMR (300 MHz, chloroform-d): δ 12.33 (br. s., 1 H), 7.39 - 7.36 (m, 1 H), 7.35 - 7.30 (m, 2 H), 7.29 (d, *J* = 2.9 Hz, 2 H), 7.25 (s, 1 H), 7.19 - 7.17 (m, 2 H), 7.10 - 7.07 (m, 1 H), 4.96 (t, *J* = 12.0 Hz, 1 H), 4.84 (s, 2 H), 4.74 (s, 2 H), 4.20 (br. s., 1 H), 3.57 (d, *J* = 11.7 Hz, 2 H), 3.17 - 3.10 (m, 2 H), 3.06 - 2.99 (m, 2 H), 2.76 (t, *J* = 10.3 Hz, 2 H), 2.41 - 2.23 (m, 4 H), 1.79 (d, *J* = 12.3 Hz, 2 H), 1.16 (t, *J* = 7.3 Hz, 3 H)

^{13}C NMR (125 MHz, chloroform-d): δ 175.7, 137.9, 136.2, 135.7, 130.3, 129.2, 128.7, 128.1, 127.63, 127.58, 125.1, 62.9, 58.6, 52.8, 48.7, 43.2, 30.7, 27.0, 26.4, 9.7

HRMS (ESI) calculated for $C_{24}H_{32}N_2O_2$ $[M+H]^+$ 381.253655, observed 381.253636

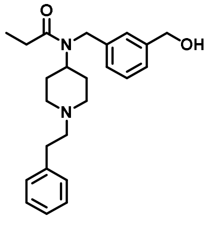

N-(3-(hydroxymethyl)benzyl)-N-(1-phenethylpiperidin-4-yl)propionamide (2/RO76)

Yield: 42%

^{1}H NMR (400 MHz, chloroform-d): δ 12.02 (br. s., 1 H), 7.31 – 7.28 (m, 3 H), 7.25 – 7.21 (m, 3 H), 7.15 (d, *J* = 7.0 Hz, 2 H), 7.05 (d, *J* = 7.0 Hz, 1 H), 4.85 - 4.78 (m, 1 H), 4.66 (s, 2 H), 4.54 (s, 2 H), 3.6 (d, *J* = 10.9 Hz, 2 H), 3.26 -

3.22 (m, 1 H), 3.15 – 3.11 (m, 2 H), 3.02 - 2.98 (m, 2 H), 2.76 (t, *J* = 10.5 Hz, 2 H), 2.37 (d, *J* = 7.0 Hz, 2 H), 2.15 - 2.06 (m, 2 H), 1.8 (d, *J* = 12.8 Hz, 2 H), 1.13 (t, *J* = 7.0 Hz, 3 H)

^{13}C NMR (100 MHz, chloroform-d): δ 175.4, 142.3, 137.9, 135.7, 129.2, 129.1, 128.8, 128.7, 127.6, 126.4, 124.7, 64.8, 58.5, 52.7, 49.1, 46.7, 30.6, 27.2, 26.7, 9.7

HRMS (ESI) calculated for $C_{24}H_{32}N_2O_2$ $[M+H]^+$ 381.253655, observed 381.253522

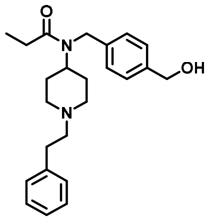

N-(4-(hydroxymethyl)benzyl)-N-(1-phenethylpiperidin-4-yl)propionamide (3)

Yield: 45%

^{1}H NMR (500 MHz, chloroform-d): δ 12.15 (br. s., 1 H), 7.32 (d, *J* = 7.8 Hz, 2 H), 7.29 (d, *J* = 7.8 Hz, 2 H), 7.24 (t, *J* = 7.1 Hz,1 H), 7.15 (d, *J* = 7.3 Hz, 2 H), 7.12 (d, *J* = 7.8 Hz, 2 H), 4.82 (t, *J* = 12.2 Hz, 1 H), 4.65 (s, 2 H), 4.52 (s, 2 H), 4.10 (br.s., 1H), 3.58 (d, *J* = 11.2 Hz, 2 H), 3.15 – 3.11 (m, 2 H), 3.01 - 2.98 (m, 2 H), 2.75 (t, *J* = 11.7 Hz, 2 H), 2.33 (q, *J* = 7.3 Hz, 2 H), 2.08 (q, *J* = 12.7 Hz, 2 H), 1.74 (d, *J* = 13.2 Hz, 2 H), 1.12 (t, *J* = 7.3 Hz, 3 H)

^{13}C NMR (100 MHz, chloroform-d): δ 175.4, 140.7, 136.9, 135.8, 129.2, 128.7, 127.7, 127.5, 125.9, 64.7, 58.4, 52.6, 48.9, 46.3, 30.6, 27.1, 26.6, 9.6

HRMS (ESI) calculated for $C_{24}H_{32}N_2O_2$ $[M+H]^+$ 381.253655, observed 381.253473

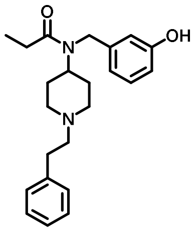

N-(3-hydroxybenzyl)-N-(1-phenethylpiperidin-4-yl)propionamide (4)

Yield: 35%

^{1}H NMR (400 MHz, chloroform-d): δ 11.24 (br. s., 1 H), 7.24 – 7.18 (m, 3 H), 7.11 – 7.08 (m, 3 H), 6.73 (d, *J* = 7.8 Hz, 1 H), 6.63 (s, 1 H), 6.58 (d, *J* = 7.8 Hz, 1 H), 4.70 – 4.64 (m, 1 H), 4.38 (s, 2 H), 3.50 (d, *J* = 10.1 Hz, 2 H), 3.42

(s, 1 H), 3.12 – 3.08 (m, 2 H), 2.96 - 2.92 (m, 2 H), 2.80 -2.73 (m, 2 H), 2.30 - 2.25 (m, 2 H), 2.10 -2.02 (m, 2 H), 1.74 (d, *J* = 12.1 Hz, 2 H), 1.05 (t, *J* = 7.0 Hz, 3 H)

^{13}C NMR (100 MHz, chloroform-d): δ 175.8, 157.6, 139.2, 135.6, 130.2, 129.1, 128.7, 127.5, 116.9, 114.8, 112.9, 58.3, 52.5, 50.4, 49.3, 46.6, 30.5, 27.1, 26.5, 9.6

HRMS (ESI) calculated for $C_{23}H_{30}N_2O_2$ $[M+H]^+$ 367.238005, observed 367.237924

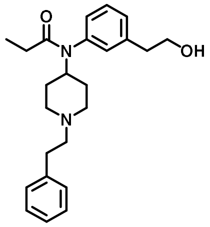

N-(3-(2-hydroxyethyl)phenyl)-N-(1-phenethylpiperidin-4-yl)propionamide (5)

Yield: 48%

^{1}H NMR (500 MHz, chloroform-d): δ 12.07 (br. s., 1H), 7.38 - 7.34 (m, 1H), 7.31 - 7.29 (m, 2H), 7.27 (s, 1H), 7.25 (d, *J* = 2.4 Hz, 1H), 7.15 (d, *J* = 7.3 Hz, 2H), 7.01 (br. s., 1H), 6.87 (d, *J* = 7.8 Hz, 1H), 4.96 - 4.90 (m, 1H), 3.95 (br. s., 1H), 3.82 (br. s., 1H), 3.69 (d, *J* = 12.2 Hz, 1H), 3.60 (d, *J* = 13.2 Hz, 1H), 3.23 - 3.17 (m, 1H), 3.10 – 3.04 (m, 1H), 2.98 (t, *J* = 7.8 Hz, 2H), 2.85 (br. s., 2H), 2.79 (br. s., 2H), 2.29 - 2.21 (m, 1H), 2.10 – 1.99 (m, 3H), 1.87 - 1.84 (m, 1H), 1.75 - 1.68 (m, 1H), 1.03 (t, *J* = 7.32 Hz, 3H)

^{13}C NMR (100 MHz, chloroform-d): δ 174.5, 142.1, 136.8, 135.7, 131.6, 129.8, 129.7, 129.2, 128.7, 127.8, 127.6, 58.5, 53.2, 52.3, 48.8, 30.7, 28.5, 27.5, 27.4, 9.7

HRMS (ESI) calculated for $C_{24}H_{32}N_2O_2$ $[M+H]^+$ 381.253655, observed 381.253385

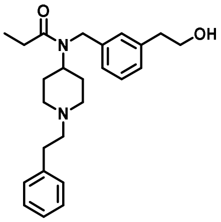

N-(3-(2-hydroxyethyl)benzyl)-N-(1-phenethylpiperidin-4-yl)propionamide (6)

Yield: 55%

^{1}H NMR (400 MHz, chloroform-d): δ 12.21 (br. s., 1 H), 7.29 - 7.26 (m, 2 H), 7.24 - 7.20 (m, 2 H), 7.15 - 7.10 (m, 3 H), 7.02 (br. s., 1 H), 6.97 (d, *J* = 7.4 Hz, 1 H), 4.98 (br. s., 1 H), 4.81 (t, *J* = 11.5 Hz, 1 H), 4.51 (s, 2 H), 3.82 (t, *J*

= 6.0 Hz, 2 H), 3.58 (d, J = 10.9 Hz, 2 H), 3.14 - 3.10 (m, 2 H), 3.0 - 2.97 (m, 2 H), 2.83 - 2.73 (m, 4 H), 2.34 (q, J = 7.0 Hz, 2 H), 2.10 (q, J = 7.0 Hz, 2 H), 1.79 (d, J = 12.8 Hz, 2 H), 1.11 (t, J = 7.0 Hz, 3 H)

^{13}C NMR (100 MHz, chloroform-d): δ 175.3, 139.9, 137.7, 135.8, 129.1, 128.7, 128.3, 127.4, 126.7, 123.6, 63.2, 58.3, 52.5, 49.0, 46.5, 39.1, 30.5, 27.1, 26.6, 9.6

HRMS (ESI) calculated for $C_{25}H_{34}N_2O_2$ [M+H]$^+$ 395.269305, observed 395.269259

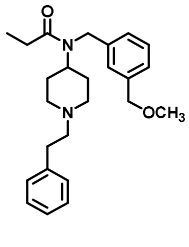

N-(3-(methoxymethyl)benzyl)-N-(1-phenethylpiperidin-4-yl)propionamide (7)

Yield: 40%

^{1}H NMR (400 MHz, chloroform-d): δ 12.71 (br. s., 1 H), 7.33 - 7.27 (m, 3 H), 7.23 (t, J = 7.0 Hz, 2 H), 7.16 (d, J = 7.0 Hz, 2 H), 7.13 (s, 1 H), 7.06 (d, J = 7.4 Hz, 1 H), 4.86 (t, J = 11.9 Hz, 1 H), 4.54 (s, 2 H), 4.42 (s, 2 H), 3.62 (d, J = 11.3 Hz, 2 H), 3.38 (s, 3 H), 3.16 - 3.12 (m, 2 H), 3.04 – 3.00 (m, 2 H), 2.75 (t, J = 11.7 Hz, 2 H), 2.31 (q, J = 7.3 Hz, 2 H), 2.15 (q, J = 12.1 Hz, 2 H), 1.79 (d, J = 12.8 Hz, 2 H), 1.11 (t, J = 7.2 Hz, 3 H)

^{13}C NMR (100 MHz, chloroform-d): δ 175.4, 139.3, 138.0, 135.8, 129.14, 129.11, 128.7, 127.5, 126.9, 124.8, 74.5, 58.4, 58.4, 52.6, 48.9, 46.3, 30.6, 27.1, 26.6, 9.6

HRMS (ESI) calculated for $C_{25}H_{34}N_2O_2$ [M+H]$^+$ 395.269305, observed 395.269178

General synthetic procedure of N-alkylation followed by propionylation (procedure no. 2)

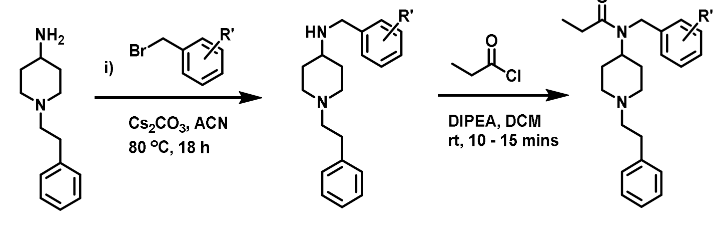

To a solution of 1-phenethylpiperidin-4-amine (0.587 mmol) and substituted benzyl bromides (0.587 mmol) in 5 mL acetonitrile was added cesium carbonate (1.17 mmol). The reaction mixture was heated at 80 °C overnight and then filtered through celite bed, further washed with 20% MeOH: DCM solvent system. The solution was concentrated in vacuo and the solid obtained carried forward to the next step without any purification. The intermediate was then

dissolved in dry DCM (5 mL) and to it DIPEA (1.17 mmol) was added followed by propionyl chloride (0.528 mmol). The reaction mixture was stirred at room temperature for 10 to 15 mins and quenched with ice water after completion of reaction. The reaction mixture was extracted with 10% MeOH: DCM (5 mL x 3), washed with saturated $NaHCO_3$ (5 mL), brine (5 mL) and dried over Na_2SO_4. The organic layer was evaporated in vacuo and purified by combi flash chromatography (DCM/Methanol – 0 to 20%) to obtain RO76 analogues. These compounds further purified using reverse phase column chromatography (solvent system - acetonitrile with 0.05%TFA: H_2O with 0.05%TFA) afforded colorless sticky compound as a TFA salt.

Characterization of compounds synthesized using N-alkylation followed by propionylation (procedure no. 2)

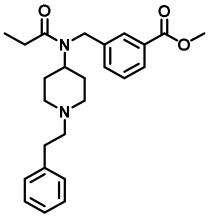

Methyl 3-((N-(1-phenethylpiperidin-4-yl)propionamido)methyl)benzoate (8/RO152)

Yield: 39%

^{1}H NMR (400 MHz, chloroform-d): δ 11.67 (br. s., 1 H), 7.93 (d, J = 7.4 Hz, 1 H), 7.80 (s, 1 H), 7.42 (t, J = 7.4 Hz, 1 H), 7.36 (d, J = 7.0 Hz, 1 H), 7.29 - 7.20 (m, 3 H), 7.14 (d, J = 7.4 Hz, 2 H), 4.87 (t, J = 10.1 Hz, 1 H), 4.57 (s, 2 H), 3.88 (s, 3 H), 3.63 (d, J = 11.3 Hz, 2 H), 3.17 (t, J = 7.8 Hz, 2 H), 2.99 (t, J = 7.6 Hz, 2 H), 2.81 (t, J = 11.3 Hz, 2 H), 2.30 (q, J = 7.0 Hz, 2 H), 2.10 (q, J = 11.8 Hz, 2 H), 1.82 (d, J = 12.8 Hz, 2 H), 1.11 (t, J = 6.8 Hz, 3 H)

^{13}C NMR (100 MHz, chloroform-d): δ 175.7, 166.8, 138.1, 135.5, 131.0, 130.3, 129.3, 129.2, 129.0, 128.7, 127.6, 126.4, 58.5, 52.7, 52.4, 49.0, 46.2, 30.6, 27.1, 26.5, 9.6

HRMS (ESI) calculated for $C_{25}H_{32}N_2O_3$ $[M+H]^+$ 409.248569, observed 409.248433

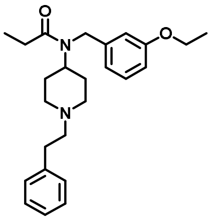

N-(3-ethoxybenzyl)-N-(1-phenethylpiperidin-4-yl)propionamide (9)

Yield: 43%

^{1}H NMR (400 MHz, chloroform-d): δ 12.91 (br. s., 1 H), 7.32 - 7.29 (m, 2 H), 7.25 - 7.21 (m, 2 H), 7.17 (d, *J* = 7.0 Hz, 2 H), 6.78 (d, *J* = 8.2 Hz, 1 H), 6.72 (d, *J* = 7.8 Hz, 1 H), 6.68 (s, 1 H), 4.86 (t, *J* = 12.1 Hz, 1 H), 4.51 (s, 2 H), 4.00 (q, *J* = 7.0 Hz, 2 H), 3.63 (d, *J* = 11.3 Hz, 2 H), 3.17 - 3.13 (m, 2 H), 3.06 – 3.02 (m, 2 H), 2.74 (t, *J* = 11.5 Hz, 2 H), 2.33 (q, *J* = 7.4 Hz, 2 H), 2.17 (q, *J* = 12.3 Hz, 2 H), 1.82 (d, *J* = 13.2 Hz, 2 H), 1.41 (t, 7.0 Hz, 3 H), 1.12 (t, *J* = 7.4 Hz, 3 H)

^{13}C NMR (100 MHz, chloroform-d): δ 173.9, 159.6, 139.5, 135.9, 130.1, 129.2, 128.8, 127.5, 117.7, 113.3, 112.0, 63.6, 58.5, 52.6, 48.9, 46.3, 30.7, 27.1, 26.6, 14.9, 9.7

HRMS (ESI) calculated for $C_{25}H_{34}N_2O_2$ $[M+H]^+$ 395.269305, observed 395.269181

Procedure for the synthesis of 3-((N-(1-phenethylpiperidin-4-yl)propionamido)methyl)benzoic acid (10)

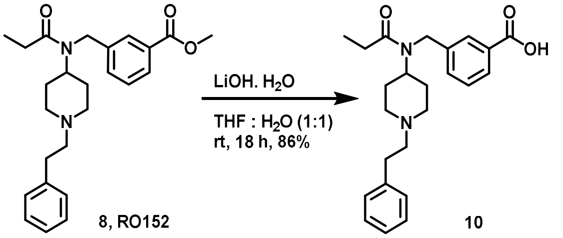

To a solution of **8**, **RO152** (36 mg, 0.088 mmol) in THF: H_2O (3 mL:3 mL), lithium hydroxide monohydrate (7.4 mg, 0.176 mmol) was added, and reaction mixture stirred at RT overnight. Then solvents were evaporated, and the product was acidified by the addition of 1N HCl (1.5 mL) and extracted with 20% MeOH: DCM (5 mL x 2). The combined organic layers were dried over Na_2SO_4 then concentrated in vacuo. The final product was purified by reverse phase column chromatography (solvent system - acetonitrile with 0.05%TFA: H_2O with 0.05%TFA) with 45 min runtime offered compound **10** (30 mg, 86%) as a colorless transparent compound as a TFA salt.

^{1}H NMR (400 MHz, chloroform-d): δ 11.95 (br. s., 1 H), 7.89 (d, *J* = 7.0 Hz, 1 H), 7.81 (s, 1 H), 7.44 - 7.38 (m, 2 H), 7.31 - 7.27 (m, 2 H), 7.24 - 7.22 (m, 1 H), 7.17 (d, *J* = 7.0 Hz, 2 H), 4.98 – 4.93 (m, 1 H), 4.59 (s, 2 H), 3.74 (d, *J* = 11.3 Hz, 2 H), 3.21 - 3.17 (m, 2 H), 3.06 – 3.03 (m, 2 H), 2.81 (t, *J* = 11.5 Hz, 2 H), 2.30 (q, *J* = 7.0 Hz, 2 H), 2.20 – 2.11 (m, 2 H), 1.85 (d, *J* = 13.2 Hz, 2 H), 1.12 (t, *J* = 7.2 Hz, 3 H)

^{13}C NMR (100 MHz, chloroform-d): δ 174.0, 157.2, 138.1, 135.7, 130.9, 130.6, 129.4, 129.3, 129.2, 128.7, 127.5, 126.9, 58.5, 52.7, 48.9, 46.3, 30.6, 27.2, 26.6, 9.6

HRMS (ESI) calculated for $C_{24}H_{30}N_2O_3$ $[M+H]^+$ 395.232919, observed 395.232899

2nd Section: Material and Methods

Chemicals and Buffers.

Various chemicals and buffers required for assays were purchased from Sigma-Aldrich, Cayman chemicals, Fischer Scientific, Greiner Bio-One, Invitrogen, gibco.

BRET based assays (TRUPATH and arrestin signaling)[3-4]

To measure G proteins dissociation or β-arrestin recruitment, 2.5 million cells were plated in 10 cm dishes and incubated overnight at 37 ºC. The next day, cells were co-transfected, using Lipofectamine 2000 following vendor protocol. Human MOR, Gα-RLuc8, Gβ, Gγ-GFP2 plasmids (2.5 µg per construct) were transfected at a ratio of 1:1:1:1 for G protein dissociation and a 1:5 DNA ratio of Rluc tagged MOR, Venus-tagged N-terminal β-arrestin (1 or 2) was used for β-arrestin recruitment. The following day, transfected cells were plated into a 384-well Poly-D-lysine coated plate with DMEM supplemented with 1% dialyzed FBS at a density of 20,000 cells per well and incubated overnight at 37 ºC. On the day of the assay, the media was removed, and cells were rinsed with 30 µL of assay buffer (1×Hank's balanced salt solution (HBSS), 20 mM HEPES, pH 7.4), followed by the addition of the 30 µL substrate buffer (7.85 µM coelenterazine 400a for G-protein or 7.85 µM coelenterazine h for β-arrestin in assay buffer) for 5 min in the dark at room temperature. After a 5 min equilibration period, the cells were treated with 15 µL of drug (3X drug concentration in assay buffer with 0.3% bovine serum albumin) for an additional 5 min in the dark at room temperature. The plates were then read four times in a BioTek Synergy Neo Alpha plate reader using 395 nm (RLuc8-coelenterazine 400a) and 510 nm (GFP2) emission filters and the measurements from the 10 min read were used for G protein analyses. For β-arrestin, the plates were read six times using 480 (RLuc8-coelenterazine h) and 530 nm (YFP2) emission filters and the measurements from the 15 min read were used for β-arrestin recruitment analyses. The ratio of GFP2/RLuc8 for G protein and eYFP/RLuc for β-arrestin were calculated per well in quadruplicates and plotted as a function of drug concentration, normalized to % control agonist stimulation and analyzed using "log(agonist) *vs.* response" in GraphPad Prism 10.0.

Nb39 assay[5]

HEK293T cells were transiently transfected with MOR-Rluc8 and Nb39-Venus in a 1:3 ratio, after 16 hrs cells were plated onto a poly-D-lysine coated 384-well plate in DMEM supplemented with 1% dialyzed FBS at 20,000 cells/well in 40 µl. After a further 24 hrs, cells were washed with 20 µl assay buffer (1X HBSS, 20 mM HEPES) then incubated with 20 µl per well coelenterazine h (5 µM, in assay buffer) for 5 min in the dark. Drugs were diluted to 30 µM in drug buffer (assay buffer + 0.3 g/ml BSA), 10 µl was added onto the plate (final concentration of 10 µM in the well) and incubated for 5 min in the dark. The plate was then immediately read for luminescence at 480 nm and Venus emission at 530 nm on a BioTek Synergy Neo plate reader. The ratio of Venus/Rluc8 signal was calculated, Nb39 recruitment was calculated by subtracting the mean ratio of the wells that received DMSO vehicle control.

Cryo-EM

Expression and purification of the µ-opioid receptor (µOR)

The µOR construct was expressed and purified as previously described[6]. Briefly, receptor was expressed in *Spodoptera frugiperda (Sf9)* insect cells using the baculovirus method (Expression Systems), and media was supplemented with 1 µM Naloxone. Cells expressing µOR were harvested and solubilized from membranes using 20 mM hydroxy-ethylpiperazine ethane sulfonic acid (HEPES), pH 7.4, 100 mM sodium chloride (NaCl), 1% n-dodecyl-β-D-maltopyranoside (DDM), 0.1% cholesteryl hemisuccinate (CHS), 2 mM $MgCl_2$, 1 µM Naloxone and protease inhibitors. Membranes were homogenized with a douncer, stirred for one hour at 4 degrees, and the soluble fraction was isolated by centrifugation and applied to a nickel-chelating sepharose resin. The Ni-NTA elution in 0.1%DDM/0.01%CHS was then incubated with 0.1% lauryl maltose neopentyl glycol (LMNG)/0.01% CHS for 1 hour on ice to exchange the detergents. After detergent exchange, 2 mM $CaCl_2$ was added, and the sample was loaded onto M1 anti-Flag resin and washed with progressively lower concentrations of salt and naloxone. The µOR was then eluted from M1 resin in a buffer consisting of 20 mM Hepes pH 7.5, 100 mM NaCl, 0.003% L-MNG/0.0003% CHS supplemented with 1 uM naloxone, Flag peptide and 5 mM EDTA. The M1 elute was further purified by size exclusion chromatography on the Superdex 200 10/300 gel filtration column (GE Healthcare) in 20 mM HEPES pH 7.5, 100 mM NaCl, 0.003% L-MNG/0.0003% CHS. The monomeric fractions were pooled, concentrated, and flash frozen in liquid nitrogen.

Expression and purification of heteromeric G_{i1}

Heterotrimeric Gi was expressed and purified as previously described.[7] Briefly, heterotrimeric G_{i1} was expressed in *Trichoplusia ni (T.ni)* insect cells using the baculovirus method (Expression Systems). Two viruses were used to infect the insect cells, one encoding the wild-type human $G_{\alpha i1}$ subunit and another one encoding the wild-type human β1γ2 subunits. The cells were harvested 48 hours post-transfection, and the pellet was flash frozen with liquid nitrogen and stored at -80°C. Cells lysis was conducted in hypotonic buffer. Membranes were harvested by centrifugation and solubilized using a douncer in 20 mM Hepes pH 7.4, 100 mM NaCl, 1% sodium cholate, 0.05% DDM, 5 mM magnesium chloride, 5 mM bME, 5 mM imidazole, 20 mM GDP and protease inhibitors. The solubilization mixture was stirred for one hour at 4 degrees. After centrifugation, the supernatant was loaded on a Ni-NTA chromatography column, washed in 0.05% DDM buffer to remove the cholate. After elution using 250 mM imidazole, human rhinovirus 3C protease (3C protease) was added and the histidine tag was cleaved overnight at 4 °C during dialysis. Then the heterotrimeric Gi without tag will be further purified through reverse Ni-NTA chromatography. Gi heterotrimer was separated from excess betagamma using a MonoQ 5/50 GL column (GE Healthcare). The protein was diluted to lower the imidazole concentration and loaded onto the column in 20 mM Hepes pH 7.4, 50 mM NaCl, 1 mM $MgCl_2$, 0.05% DDM/CHS, 100 mM TCEP and 20 mM GDP. The heterotrimer was eluted with a linear gradient of 0–50% with buffer containing 1 M NaCl. Eluted fractions were concentrated to 200-250 uM and after addition of 20% glycerol the protein was flash frozen and stored at -80 °C.

Expression and Purification of scFv16

scFv16 was developed and purified as previously described.[8] Basically, scFv with C terminal His tag was expressed in *Trichuplusia ni* Hi5 insect cells. After infection and expression, the insect cell supernatant was loaded onto Ni-NTA resin and the scFv was eluted in 20 mM HEPES pH 7.5, 500 mM NaCl, and 250 mM imidazole. The eluate was incubated with 3C protease overnight to cleave the C-terminal His tag. After dialysis into the buffer consisting of 20 mM HEPES pH 7.5 and 100 mM NaCl, scFv16 was further purified by reverse Ni-NTA chromatography. The flow-through was collected and applied over a Superdex 200 16/60 column (GE Healthcare). The scFv16 fractions were pooled, concentrated, and flash frozen.

Formation and purification of the μOR-Gi1-scfv16 complex for Cryo-EM

μOR was incubated with 10-fold molar excess of RO76 ligand for 1 hour on ice. A 1.5-fold molar excess of G_{i1} was first incubated with 1% LMNG/0.1% CHS for 1 h on ice, and then the RO76 bound μOR and G_{i1} were mixed and incubate on ice for another hour. Afterward, Apyrase (1-unit, NEB) was added to catalyze GDP hydrolysis, 1 hour later 2-fold molar excess of scfv16 was added to the complex, and the complex was incubated overnight on ice. The following day, the complex was diluted in 20 mM Hepes pH 7.4, 100 mM NaCl, 0.003% LMNG/0.0003%CHS, 10 uM RO76, 2 mM Ca^{2+} and loaded onto M1 anti-FLAG affinity chromatography. Detergent concentration was lowered by washing with buffer containing 20 mM Hepes pH 7.4, 100 mM NaCl, 0.001% LMNG/0.0001%CHS, 2 mM Ca^{2+}, 10 uM RO76. Complex was eluted in 20 mM Hepes pH 7.4, 100 mM NaCl, 0.00075% LMNG/0.000075% CHS, FLAG peptide, 5 mM EDTA, 10 uM RO76. Free receptor was separated from the complex by size exclusion chromatography on a Superdex 200 10/300 Increase column in 20 mM Hepes pH 7.4, 100 mM NaCl, 0.00075% LMNG/0.000075% CHS, 1 uM RO76. Peak fractions were concentrated to 15-20 mg/ml, filtered, and used for electron microscopy experiments.

Cryo-EM data collection and processing

3 μL aliquot of the μOR-Gi1-scfv16 complex was applied onto glow-discharged 300 mesh grids (Ultraufoil R1.2/1.3) and vitrified using a Vitrobot Mark IV (Thermo Fischer Scientific) under 100% humidity and 4 °C conditions. Cryo-EM data were collected on a Titan Krios electron microscope operating at 300 kV and equipped with a K3 direct electron detector. Movies were acquired with a calibrated pixel size of 0.8677 Å/pixel and a total dose of ~54.45 electrons/Å^2, fractionated across 50 frames (Figure S2, S3).

Data processing was performed using RELION 4.0.1 and cryoSPARC v4.[9-10] Initially, motion correction was carried out on the movies using RELION's built-in implementation, followed by Contrast transfer function (CTF) estimation using CTFFIND4. [11] Reference-based particle picking utilized previously determined GPCR-G protein 2D classes. 2,578,269 particles were picked, subjected to 2D classification in cryosparc to remove low-quality particles, and convert back into RELION and further sorted through two rounds of 3D classification. The format convertion was done using pyem (https://github.com/asarnow/pyem). This process yielded 397,087 particles and a 4.7 Å resolution structure after 3D refinement.

Subsequent steps include 2 rounds of CtfRefine, particle polishing. After that, the structure reached 3.2 Å resolution. The particles were then sorted using 3D classification without image alignment, using a mask to exclude the micelle and flexible alpha-helical domain, which generated 228,101 particles and a 3.1 Å map. To improve the local resolution of the receptor, we performed another round of 3D classification without image alignment with mask only on the receptor part. After that 127,261 particles left and that can be refined into a 3.2 Å map but with better receptor density. The resulting map was sharpened using the Phenix auto sharpen function to enhance map quality.[12]

Lastly, local resolution estimation and 3DFSC were employed to assess the local resolution and orientation distribution of the final dataset.[13-14] (Figure S2-B, C)

Model building and refinement

The individual structures of μOR-G_{i1} heterotrimer, and scfv16 were independently docked into the final sharpened map. Model and geometric restraints for RO76 were generated using the Phenix elbow tool.[15] The model was iteratively refined and validated through multiple rounds of Phenix real-space refinement and manual refinement in Coot (Figure S3).[16-17]

System setup for MD simulations

We performed simulations of μOR bound to LFT, MP, RO76, or RO152. The LFT and MP simulations were the same as in our previous work..[18] We initiated the RO76 and RO152 simulations using the cryoEM structure from this manuscript of RO76 bound to the μOR-Gi1-scFv16 complex. For the latter simulations, we manually changed RO76 to RO152 using Maestro (Schrodinger Release 2023-2: Maestro, Schrodinger, 2023). We removed the Gi1 and scFv16 proteins before simulation. For each ligand, we performed six independent simulations, each initiated with random and independent initial atom velocities. The RO76 and RO152 simulations were each 2 μs in length. Our previous MP and LFT simulations were 3.5 μs in length; to ensure consistency, we only considered the first 2 μs of each simulation in our analysis.

For all simulation conditions, the protein structures were aligned to the Orientations of Proteins in Membranes entry for 5C1M (active μOR bound to BU72 using PyMOL, and crystal waters from 5C1M were incorporated).[19] Prime (Schrödinger) was used to model missing side chains, and to add capping groups to protein chain termini. All ligand parameters were generated using the Paramchem webserver. Protonation states of all titratable residues were assigned

at pH 7, except for D114$^{2.50}$ and D164$^{3.49}$, which were protonated (neutral) in all simulations, as these conserved residues are reported to be protonated in the active states of several class A GPCRs.[20-21] Histidine residues were modeled as neutral, with a hydrogen atom bound to the epsilon nitrogen, as we did not find any cases where moving the hydrogen to the delta nitrogen would help optimize the local hydrogen bonding network. Using Dabble, the prepared protein structures were inserted into a pre-equilibrated palmitoyl-oleoyl-phosphatidylcholine (POPC) bilayer, the system was solvated, and sodium and chloride ions were added to neutralize the system and to obtain a final concentration of 150 mM. The final systems comprised approximately 59,000 atoms, and system dimensions were approximately 80 × 80 × 90 $Å^3$.

MD simulation and analysis protocols

We used the CHARMM36m force field for proteins, the CHARMM36 force field for lipids and ions, and the TIP3P model for waters.[22-23] All simulations were performed using the Compute Unified Device Architecture (CUDA) version of particle-mesh Ewald (PME) MD in AMBER20 on graphics processing units (GPUs).[24]

Systems were first minimized using three rounds of minimization, each consisting of 500 cycles of steepest descent followed by 500 cycles of conjugate gradient optimization. Thus, 10.0 and 5.0 kcal mol^{-1} $Å^{-2}$ harmonic restraints were applied to protein, lipids and ligand for the first and second rounds of minimization, respectively. Further, 1 kcal mol^{-1} $Å^{-2}$ harmonic restraints were applied to protein and ligand for the third round of minimization. Systems were then heated from 0 K to 100 K in the constant number (N), constant-volume (V) and constant-temperature (T) or NVT ensemble over 12.5 ps and then from 100 K to 310 K in the constant number (N), pressure (P) and constant-temperature (T) or NPT ensemble over 125 ps, using 10.0 kcal mol^{-1} $Å^{-2}$ harmonic restraints applied to protein- and ligand-heavy atoms. Subsequently, systems were equilibrated at 310 K and 1 bar in the NPT ensemble, with harmonic restraints on the protein and ligand nonhydrogen atoms tapered off by 1.0 kcal mol^{-1} $Å^{-2}$ starting at 5.0 kcal mol^{-1} $Å^{-2}$ in a step-wise fashion every 2 ns for 10 ns, and then by 0.1 kcal mol^{-1} $Å^{-2}$ every 2 ns for 20 ns. Production simulations were performed without restraints at 310 K and 1 bar in the NPT ensemble using the Langevin thermostat and the Monte Carlo barostat, and using a timestep of 4.0 fs with hydrogen mass repartitioning. Bond lengths were constrained using the SHAKE algorithm.[25] Nonbonded interactions were cut off at 9.0 Å, and long-range electrostatic interactions were calculated using the PME method with an Ewald coefficient of approximately 0.31 Å, and 4th order B-splines.

The PME grid size was chosen such that the width of a grid cell was approximately 1 Å. The use of a 9.0-Å nonbonded interaction cutoff and a 4-fs timestep with hydrogen mass repartitioning may have affected the structural and kinetic properties of the lipid bilayer, potentially introducing artifacts in our simulations.

The AmberTools17 CPPTRAJ package was used to reimage trajectories to 1 ns per frame.[26] Simulations were visualized and analyzed using Visual Molecular Dynamics (VMD) and PyMOL (Schrodinger).[27]

Hydrogen bond contacts were measured using the GetContacts package, which sets the donor-to-acceptor distance as less than 3.5 Å and donor-hydrogen-acceptor angle as greater than 110 degrees.[28] Water-mediated bonds were calculated, as defined by GetContacts, as interactions where the two contact regions hydrogen bond to either the same water molecule or to two consecutive water molecules that form an extended water bridge. For this paper, interactions with one water or two waters were considered separately, with plots showing only a single water interaction unless otherwise specified. When the frequencies of both conditions were calculated, frames with both one and two waters forming interactions were categorized as single water mediated interaction frames to avoid overlap. The water mediated interactions described in this paper were calculated between the closer side-chain oxygen of $D114^{2.50}$ and either the hydroxyl of RO76 or the carbonyl of RO152. The $Y^{7.43}$-$Q^{2.60}$ hydrogen bond was calculated between the tyrosine side chain oxygen and the glutamine side chain nitrogen. Pi stacking interactions were quantified by computing the distance between the centroid of the ligand and tyrosine phenyls. Each centroid was calculated as the center of three alternating carbons on the aromatic rings. A centroid distance below 5 Å indicates the presence of pi-pi stacking. All images from MD simulation were chosen to reflect representative behavior across all simulation trajectories.

APEX reaction, biotinylated protein enrichment and preparation for mass spectrometry analysis.

HEK293T cells expressing the APEX2 enzyme fused to the human μOR (μOR-APEX) and the spatial reference APEX constructs were incubated with 500 μM biotin-phenol at 37 °C for 30 min. The receptor was activated with 10 μM RO76 over a time course of 30 min. The spatial reference APEX samples were not treated with RO76. APEX labeling was initiated pre-activation (time 0) and after 1, 5, 10 and 30 min of activation by 1:1 mixing of the H_2O_2 containing media (1 mM H_2O_2 final) with the biotin-phenol containing media at room temperature. After 45 s of the biotinylation reaction, the cells were washed three times (1 min each) with ice cold quenching buffer (PBS supplemented with 10

mM sodium ascorbate, 10 mM sodium azide, and 5 mM Trolox). Cells were then collected in 8 mL of quench buffer and pelleted by centrifugation at 4 °C for 10 min at 3000 x g. For cell lysis, cells were homogenized using probe sonication in RIPA (50 mM Tris, 150 mM NaCl, 1% Triton X-100, 0.25% sodium deoxycholate, 0.25% SDS, pH 7.4) supplemented with 10 mM sodium ascorbate, 10 mM sodium azide, 5 mM Trolox, 1 mM DTT, and protease inhibitors (Roche Complete). To remove the cell debris, cell lysate was centrifuged at 10,000 x g for 10 min, and the supernatant was taken for streptavidin enrichment of biotinylated proteins.

The enrichment of biotinylated proteins was automated with the KingFisher Flex (Thermo Fisher Scientific). Supernatants were incubated at 4 °C for 18 hrs with magnetic streptavidin beads (Pierce™ Streptavidin Magnetic Beads, Thermo Fisher Scientific) which were pre-washed twice with RIPA buffer. Following incubation, beads were washed three times with RIPA buffer, one time with 1 M KCl, one time with 0.1 M Na_2CO_3, one time with 2 M urea in 50 mM Tris-HCl (pH 8) buffer, and two times with 50 mM Tris-HCl (pH 8) buffer. Beads were maintained in 200 uL of 2 M urea in 50 mM Tris-HCl (pH 8) buffer for on-bead digestion of proteins. Samples were reduced with 5 mM TCEP at 37 °C for 30 min, followed by alkylation with 5 mM IAA at room temperature in the dark for another 30 min, which was quenched by addition of DTT (5 mM final). For tryptic digestion, 1 ug of trypsin and LysC was added to beads and incubated with shaking at 37 °C for 4 hrs. To ensure complete digestion another 0.5 ug of trypsin were added the next morning with an incubation at 37 °C with shaking. Supernatants were taken and saved for desalting using NEST C18 MicroSpin columns.

Unbiased mass spectrometric data acquisition and protein quantification for APEX samples.

μOR-APEX and spatial APEX reference samples were analyzed on an Orbitrap Exploris 480 mass spectrometry system (Thermo Fisher Scientific) coupled to a Easy nLC 1200 nano-flow ultra high-pressure liquid chromatography (Thermo Fisher Scientific) interfaced via a Nanospray Flex nanoelectrospray source. Samples were reconstituted in 1% formic acid and loaded onto a C18 column (25 cm x 75 μm I.D. packed with ReproSil Pur C18 AQ 1.9 μm particles). Mobile phase A consisted of 0.1% FA, and mobile phase B consisted of 0.1% FA/80% ACN. Peptides were separated at a flow rate of 300 nl/min using a gradient increasing buffer B over 40 min to 16% B, followed by an increase over 26 min to 28% B and 4 min to 44% B. The mass spectrometer acquired data in a data-independent acquisition (DIA) mode, collecting one full scan in the Orbitrap at 120,000 resolution followed by DIA MS/MS scans within a m/z range of 350-1050 with a fragmentation window size set to 20 m/z. The resolution of orbitrap for MS2

scans was set to 15,000 and a normalized collision energy of 30 was used for fragmentation. The DIA data were analyzed with Spectronaut (Biognosys) using direct DIA analysis default parameters for the identification and quantification of proteins. Normalization in Spectronaut was turned off. Data were searched against the Uniprot Human database. Peptide ion intensities from Spectronaut were summarized to protein intensities using the MSstats (version 4.4.1) function dataProcess with default settings except missing-value imputation was disabled and only high-quality and informative features were used (MBimpute = FALSE, remove_uninformative_feature_outlier = TRUE, featureSubset = "highQuality"). All proteins with only one quantified peptide were left out of further analysis.

Statistical analysis of APEX-MS samples

Each protein's trend over the time course after RO76 treatment was scored by fitting the log2 intensities with a continuous cubic-polynomial curve over time using the R functions lm and poly. To better fit the rapid changes, especially between time 0 and 1 minute, the collected timepoints were encoded by their ranks (1, 2, 3, 4, and 5 for 0, 1, 5, 10, and 30 minutes). The model included an additive term for the batch–a protein's background intensity was expected and allowed to vary between batches. The time-dependent model was compared with a null-model that contained only the batch term using the R function anova to compute an F statistic and p-value. The maximum mean change between time 0 and any single later time, after imputing any missing values using the fitted model, was used as the maximum log2 fold change for that protein.

Mice

Male C57BL/6J mice (24–38 g, 8-12 weeks) were purchased from Jackson Laboratories (Bar Harbor, ME). Male CD1 mice (29-45 g, 8–12 weeks of age) were purchased from Charles River Laboratories (Wilmington, MA). Male MOR KO were bred in the McLaughlin laboratory at University of Florida and used when 8-12 weeks old. Progenitors of the colonies for μOR KO were obtained from Jackson Labs. All mice used throughout the manuscript were opioid naïve. All mice were maintained on a 12 h light/dark cycle with Purina rodent chow and water available ad libitum and housed in groups of five until testing. These mice were kept at a constant temperature of 22 ± 2 °C, and relative humidity was maintained at 40–50%. All animal studies reported adhere to the ARRIVE guidelines[29]. All procedures were preapproved by the Institutional Animal Care and Use Committee (University of Florida) and conducted according to the 2011 NIH Guide for the Care and Use of Laboratory Animals. C57BL/6J mice were used in assays of warm-water tail withdrawal[5, 30] and locomotor.[5, 30]

Antinociception[5,11]

The 55 °C warm-water tail-withdrawal assay was conducted in C57BL/6J mice as a measure of acute thermal antinociception as described previously.[31] Briefly, each mouse was tested for baseline tail-withdrawal latency prior to drug administration. Following drug administration, the latency for each mouse to withdraw the tail was measured every 10 min until latency returned to the baseline value. A maximum response time of 15 s was utilized to prevent tissue damage. If the mouse failed to display a tail-withdrawal response within 15 s, the tail was removed from the water and the animal was assigned a maximal antinociceptive score of 100%. Data are reported as percent antinociception, calculated by the equation: % antinociception = 100 × [(test latency−baseline latency)/(15−baseline latency)]. This was utilized to account for innate variability between mice. Compounds were administered subcutaneously (sc) or oral (po) and the analgesic action of compounds was assessed at the peak effect.

Respiratory and locomotor effects[5, 11]

Respiration rates and spontaneous ambulation rates were monitored using the automated, computer-controlled Comprehensive Lab Animal Monitoring System (CLAMS, Columbus Instruments, Columbus, OH) as described previously.[31-33] Awake, freely moving adult male mice (C57BL6/J wild-type, μOR KO) were habituated in closed, sealed individual apparatus cages (23.5 cm x 11/5 cm x 13 cm) for 60 min before testing. A baseline for each animal was obtained over the 60-min period before drug injection, and testing began immediately post-injection. Vehicle, morphine (30 mg/kg, sc), RO76 (10 and 30 mg/kg, sc) were administered subcutaneously and five min later mice were confined to the CLAMS testing cages for 140 min. Using a pressure transducer built into the sealed CLAMS cage, the respiration rate (breaths/min) of each occupant mouse was measured. Infrared beams located in the floor measured locomotion as ambulations, from the number of sequential breaks of adjacent beams. Data are expressed as percent of vehicle control response.

Pharmacokinetic Study[5]

RO76 was administered to mice sc at 30 mg/kg dose in four C57BL/6J mice. At 20 min post administration of the drug, mice were anesthetized under isoflurane, blood was removed, and animals were sacrificed for brain removal. Brains were quickly rinsed off with PBS, blot-dried and snap frozen. Tissue samples were then weighed and placed into Navy bead lysis kit tubes. Naïve tissue was used to prepare Standard, Quality control (QC) and Blanks samples

in tissue matrix. To each sample tube was added the appropriate volume of cold acetonitrile: water (3:1) to achieve a tissue concentration of 200 mg/mL. Tubes were placed in a bead beater for 3 minutes then centrifuged at 3200 rpm for 5 minutes at 4 °C. The supernatants were transferred in Eppendorf tubes and stored at −80 °C until the day of analysis. The day of the analysis, the samples were thawed on ice, mixed vigorously then centrifuged at 3200 rpm for 5 minutes at 4 °C. Supernatant (30 µl) were collected and transferred into a 96-well plate. In the same way, 30 µl of Standards, QC, Blanks and Double blanks samples freshly made in the matrix were transferred into the plate. Then cold Acetonitrile (150 µl) spiked with internal standard (IS) was added to blanks, standards, QCs and unknown samples. Only cold acetonitrile (150 µl) was added to the double blanks. Samples were mixed vigorously for 10 min then centrifuged at 3200 rpm for 10 minutes at 4 °C. Supernatant were transferred into a 96-well plate, evaporated to dryness under nitrogen at RT. Samples were reconstituted in 100 µL of 0.1% v/v formic acid in water: acetonitrile (90:10). Plate was sealed, vortexed during 5 min, briefly centrifuged then submitted for LC/MS analysis as described previously.[16]

Opioid induced withdrawal effects[34]

Animals were randomly assigned to one of the following groups: saline (i.p.), morphine (10– 75 mg/kg, i.p.), RO76 (30 mg/kg, s.c.). Dosing for experimental design, was performed on a 5-day schedule, with an escalating dose of the compound given twice daily for 4 days, with a final dose administered on the 5th day. Two hours posttreatment on the last day, all mice were administered naloxone (10 mg/kg, s.c.) and precipitated opioid withdrawal signs were assessed.[35-37]

References

1. Valdez, C. A.; Leif, R. N.; Mayer, B. P., An efficient, optimized synthesis of fentanyl and related analogs. *PLoS One* **2014,** *9* (9), e108250.
2. Faouzi, A.; Uprety, R.; Gomes, I.; Massaly, N.; Keresztes, A. I.; Le Rouzic, V.; Gupta, A.; Zhang, T.; Yoon, H. J.; Ansonoff, M.; Allaoa, A.; Pan, Y. X.; Pintar, J.; Morón, J. A.; Streicher, J. M.; Devi, L. A.; Majumdar, S., Synthesis and Pharmacology of a Novel µ-δ Opioid Receptor Heteromer-Selective Agonist Based on the Carfentanyl Template. *Journal of medicinal chemistry* **2020,** *63* (22), 13618-13637.
3. DiBerto, J. F.; Smart, K.; Olsen, R. H. J.; Roth, B. L., Agonist and antagonist TRUPATH assays for G protein-coupled receptors. *STAR protocols* **2022,** *3* (2), 101259.
4. DiBerto, J. F.; Olsen, R. H. J.; Roth, B. L., TRUPATH: An Open-Source Biosensor Platform for Interrogating the GPCR Transducerome. In *Bioluminescence: Methods and Protocols, Volume 2*, Kim, S.-B., Ed. Springer US: New York, NY, 2022; pp 185-195.
5. Chakraborty, S.; DiBerto, J. F.; Faouzi, A.; Bernhard, S. M.; Gutridge, A. M.; Ramsey, S.; Zhou, Y.; Provasi, D.; Nuthikattu, N.; Jilakara, R.; Nelson, M. N. F.; Asher, W. B.; Eans, S. O.; Wilson, L. L.; Chintala, S. M.; Filizola, M.; van Rijn, R. M.; Margolis, E. B.; Roth, B. L.; McLaughlin, J. P.; Che, T.; Sames, D.; Javitch, J. A.; Majumdar, S.,

A Novel Mitragynine Analog with Low-Efficacy Mu Opioid Receptor Agonism Displays Antinociception with Attenuated Adverse Effects. *Journal of medicinal chemistry* **2021,** *64* (18), 13873-13892.
6. Faouzi, A.; Wang, H.; Zaidi, S. A.; DiBerto, J. F.; Che, T.; Qu, Q.; Robertson, M. J.; Madasu, M. K.; El Daibani, A.; Varga, B. R.; Zhang, T.; Ruiz, C.; Liu, S.; Xu, J.; Appourchaux, K.; Slocum, S. T.; Eans, S. O.; Cameron, M. D.; Al-Hasani, R.; Pan, Y. X.; Roth, B. L.; McLaughlin, J. P.; Skiniotis, G.; Katritch, V.; Kobilka, B. K.; Majumdar, S., Structure-based design of bitopic ligands for the micro-opioid receptor. *Nature* **2023,** *613* (7945), 767-774.
7. Koehl, A.; Hu, H.; Maeda, S.; Zhang, Y.; Qu, Q.; Paggi, J. M.; Latorraca, N. R.; Hilger, D.; Dawson, R.; Matile, H.; Schertler, G. F. X.; Granier, S.; Weis, W. I.; Dror, R. O.; Manglik, A.; Skiniotis, G.; Kobilka, B. K., Structure of the micro-opioid receptor-G(i) protein complex. *Nature* **2018,** *558* (7711), 547-552.
8. Maeda, S.; Koehl, A.; Matile, H.; Hu, H.; Hilger, D.; Schertler, G. F. X.; Manglik, A.; Skiniotis, G.; Dawson, R. J. P.; Kobilka, B. K., Development of an antibody fragment that stabilizes GPCR/G-protein complexes. *Nat Commun* **2018,** *9* (1), 3712.
9. Kimanius, D.; Dong, L.; Sharov, G.; Nakane, T.; Scheres, S. H. W., New tools for automated cryo-EM single-particle analysis in RELION-4.0. *The Biochemical journal* **2021,** *478* (24), 4169-4185.
10. Punjani, A.; Rubinstein, J. L.; Fleet, D. J.; Brubaker, M. A., cryoSPARC: algorithms for rapid unsupervised cryo-EM structure determination. *Nat Methods* **2017,** *14* (3), 290-296.
11. Rohou, A.; Grigorieff, N., CTFFIND4: Fast and accurate defocus estimation from electron micrographs. *J Struct Biol* **2015,** *192* (2), 216-21.
12. Terwilliger, T. C.; Sobolev, O. V.; Afonine, P. V.; Adams, P. D., Automated map sharpening by maximization of detail and connectivity. *Acta Crystallogr D Struct Biol* **2018,** *74* (Pt 6), 545-559.
13. Tan, Y. Z.; Baldwin, P. R.; Davis, J. H.; Williamson, J. R.; Potter, C. S.; Carragher, B.; Lyumkis, D., Addressing preferred specimen orientation in single-particle cryo-EM through tilting. *Nat Methods* **2017,** *14* (8), 793-796.
14. Cardone, G.; Heymann, J. B.; Steven, A. C., One number does not fit all: mapping local variations in resolution in cryo-EM reconstructions. *J Struct Biol* **2013,** *184* (2), 226-36.
15. Moriarty, N. W.; Grosse-Kunstleve, R. W.; Adams, P. D., electronic Ligand Builder and Optimization Workbench (eLBOW): a tool for ligand coordinate and restraint generation. *Acta Crystallogr D Biol Crystallogr* **2009,** *65* (Pt 10), 1074-80.
16. Emsley, P.; Lohkamp, B.; Scott, W. G.; Cowtan, K., Features and development of Coot. *Acta Crystallogr D Biol Crystallogr* **2010,** *66* (Pt 4), 486-501.
17. Liebschner, D.; Afonine, P. V.; Baker, M. L.; Bunkoczi, G.; Chen, V. B.; Croll, T. I.; Hintze, B.; Hung, L. W.; Jain, S.; McCoy, A. J.; Moriarty, N. W.; Oeffner, R. D.; Poon, B. K.; Prisant, M. G.; Read, R. J.; Richardson, J. S.; Richardson, D. C.; Sammito, M. D.; Sobolev, O. V.; Stockwell, D. H.; Terwilliger, T. C.; Urzhumtsev, A. G.; Videau, L. L.; Williams, C. J.; Adams, P. D., Macromolecular structure determination using X-rays, neutrons and electrons: recent developments in Phenix. *Acta Crystallogr D Struct Biol* **2019,** *75* (Pt 10), 861-877.
18. Qu, Q.; Huang, W.; Aydin, D.; Paggi, J. M.; Seven, A. B.; Wang, H.; Chakraborty, S.; Che, T.; DiBerto, J. F.; Robertson, M. J.; Inoue, A.; Suomivuori, C. M.; Roth, B. L.; Majumdar, S.; Dror, R. O.; Kobilka, B. K.; Skiniotis, G., Insights into distinct signaling profiles of the μOR activated by diverse agonists. *Nature chemical biology* **2023,** *19* (4), 423-430.
19. Huang, W.; Manglik, A.; Venkatakrishnan, A. J.; Laeremans, T.; Feinberg, E. N.; Sanborn, A. L.; Kato, H. E.; Livingston, K. E.; Thorsen, T. S.; Kling, R. C.; Granier, S.; Gmeiner, P.; Husbands, S. M.; Traynor, J. R.; Weis, W. I.; Steyaert, J.; Dror, R. O.; Kobilka, B. K., Structural insights into μ-opioid receptor activation. *Nature* **2015,** *524* (7565), 315-321.
20. Ghanouni, P.; Schambye, H.; Seifert, R.; Lee, T. W.; Rasmussen, S. G.; Gether, U.; Kobilka, B. K., The effect of pH on beta(2) adrenoceptor function. Evidence for protonation-dependent activation. *The Journal of biological chemistry* **2000,** *275* (5), 3121-7.
21. Mahalingam, M.; Martínez-Mayorga, K.; Brown, M. F.; Vogel, R., Two protonation switches control rhodopsin activation in membranes. *Proceedings of the National Academy of Sciences of the United States of America* **2008,** *105* (46), 17795-800.
22. Huang, J.; Rauscher, S.; Nawrocki, G.; Ran, T.; Feig, M.; de Groot, B. L.; Grubmüller, H.; MacKerell, A. D., Jr., CHARMM36m: an improved force field for folded and intrinsically disordered proteins. *Nature methods* **2017,** *14* (1), 71-73.
23. Yu, Y.; Krämer, A.; Venable, R. M.; Brooks, B. R.; Klauda, J. B.; Pastor, R. W., CHARMM36 Lipid Force Field with Explicit Treatment of Long-Range Dispersion: Parametrization and Validation for Phosphatidylethanolamine, Phosphatidylglycerol, and Ether Lipids. *Journal of chemical theory and computation* **2021,** *17* (3), 1581-1595.

24. Case, D. A.; Cheatham, T. E., 3rd; Darden, T.; Gohlke, H.; Luo, R.; Merz, K. M., Jr.; Onufriev, A.; Simmerling, C.; Wang, B.; Woods, R. J., The Amber biomolecular simulation programs. *Journal of computational chemistry* **2005,** *26* (16), 1668-88.
25. Ryckaert, J.-P.; Ciccotti, G.; Berendsen, H. J. C., Numerical integration of the cartesian equations of motion of a system with constraints: molecular dynamics of n-alkanes. *Journal of Computational Physics* **1977,** *23* (3), 327-341.
26. Roe, D. R.; Cheatham, T. E., 3rd, PTRAJ and CPPTRAJ: Software for Processing and Analysis of Molecular Dynamics Trajectory Data. *Journal of chemical theory and computation* **2013,** *9* (7), 3084-95.
27. Humphrey, W.; Dalke, A.; Schulten, K., VMD: visual molecular dynamics. *Journal of molecular graphics* **1996,** *14* (1), 33-8, 27-8.
28. Venkatakrishnan, A. J.; Rasmus, F.; Anthony, K. M.; Scott, A. H.; Augustine, C.; Daniel, H.; Albert, J. K.; Ramin, A.; Babu, M. M.; Brian, K. K.; Ron, O. D., Uncovering patterns of atomic interactions in static and dynamic structures of proteins. *bioRxiv* **2019**, 840694.
29. Kilkenny, C.; Browne, W. J.; Cuthill, I. C.; Emerson, M.; Altman, D. G., Improving bioscience research reporting: the ARRIVE guidelines for reporting animal research. *PLoS biology* **2010,** *8* (6), e1000412.
30. Wilson, L. L.; Alleyne, A. R.; Eans, S. O.; Cirino, T. J.; Stacy, H. M.; Mottinelli, M.; Intagliata, S.; McCurdy, C. R.; McLaughlin, J. P., Characterization of CM-398, a Novel Selective Sigma-2 Receptor Ligand, as a Potential Therapeutic for Neuropathic Pain. *Molecules (Basel, Switzerland)* **2022,** *27* (11).
31. Váradi, A.; Marrone, G. F.; Palmer, T. C.; Narayan, A.; Szabó, M. R.; Le Rouzic, V.; Grinnell, S. G.; Subrath, J. J.; Warner, E.; Kalra, S.; Hunkele, A.; Pagirsky, J.; Eans, S. O.; Medina, J. M.; Xu, J.; Pan, Y. X.; Borics, A.; Pasternak, G. W.; McLaughlin, J. P.; Majumdar, S., Mitragynine/Corynantheidine Pseudoindoxyls As Opioid Analgesics with Mu Agonism and Delta Antagonism, Which Do Not Recruit β-Arrestin-2. *Journal of medicinal chemistry* **2016,** *59* (18), 8381-97.
32. Uprety, R.; Che, T.; Zaidi, S. A.; Grinnell, S. G.; Varga, B. R.; Faouzi, A.; Slocum, S. T.; Allaoa, A.; Varadi, A.; Nelson, M.; Bernhard, S. M.; Kulko, E.; Le Rouzic, V.; Eans, S. O.; Simons, C. A.; Hunkele, A.; Subrath, J.; Pan, Y. X.; Javitch, J. A.; McLaughlin, J. P.; Roth, B. L.; Pasternak, G. W.; Katritch, V.; Majumdar, S., Controlling opioid receptor functional selectivity by targeting distinct subpockets of the orthosteric site. *eLife* **2021,** *10*.
33. Cirino, T. J.; Eans, S. O.; Medina, J. M.; Wilson, L. L.; Mottinelli, M.; Intagliata, S.; McCurdy, C. R.; McLaughlin, J. P., Characterization of Sigma 1 Receptor Antagonist CM-304 and Its Analog, AZ-66: Novel Therapeutics Against Allodynia and Induced Pain. *Frontiers in pharmacology* **2019,** *10*, 678.
34. Wilson, L. L.; Chakraborty, S.; Eans, S. O.; Cirino, T. J.; Stacy, H. M.; Simons, C. A.; Uprety, R.; Majumdar, S.; McLaughlin, J. P., Kratom Alkaloids, Natural and Semi-Synthetic, Show Less Physical Dependence and Ameliorate Opioid Withdrawal. *Cellular and molecular neurobiology* **2021,** *41* (5), 1131-1143.
35. Kamei, J.; Ohsawa, M., Role of noradrenergic functions in the modification of naloxone-precipitated withdrawal jumping in morphine-dependent mice by diabetes. *Life sciences* **1997,** *60* (15), Pl223-8.
36. Ozdoğan, U. K.; Lähdesmäki, J.; Scheinin, M., Influence of prazosin and clonidine on morphine analgesia, tolerance and withdrawal in mice. *European journal of pharmacology* **2003,** *460* (2-3), 127-34.
37. Wilson, L. L.; Harris, H. M.; Eans, S. O.; Brice-Tutt, A. C.; Cirino, T. J.; Stacy, H. M.; Simons, C. A.; León, F.; Sharma, A.; Boyer, E. W.; Avery, B. A.; McLaughlin, J. P.; McCurdy, C. R., Lyophilized Kratom Tea as a Therapeutic Option for Opioid Dependence. *Drug and alcohol dependence* **2020,** *216*, 108310.

^{1}H-NMR and ^{13}C-NMR Spectra

^{1}H NMR of N-phenylethylpiperidin-4-one (400 MHz, $CDCl_3$)

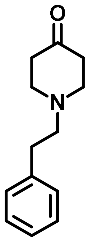

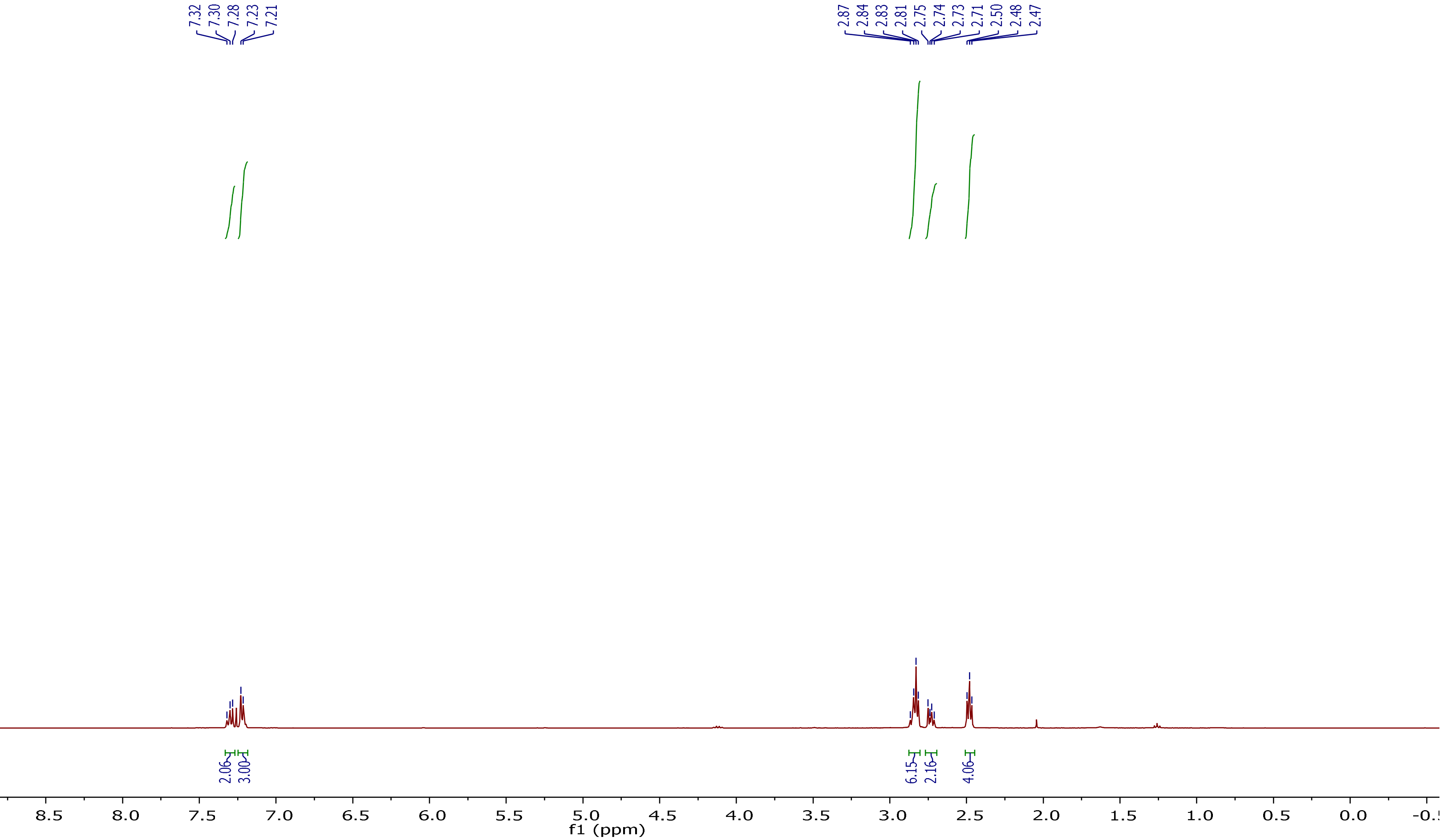

^{1}H NMR of N-(2-(hydroxymethyl)benzyl)-N-(1-phenethylpiperidin-4-yl)propionamide (1) (300 MHz, $CDCl_3$)

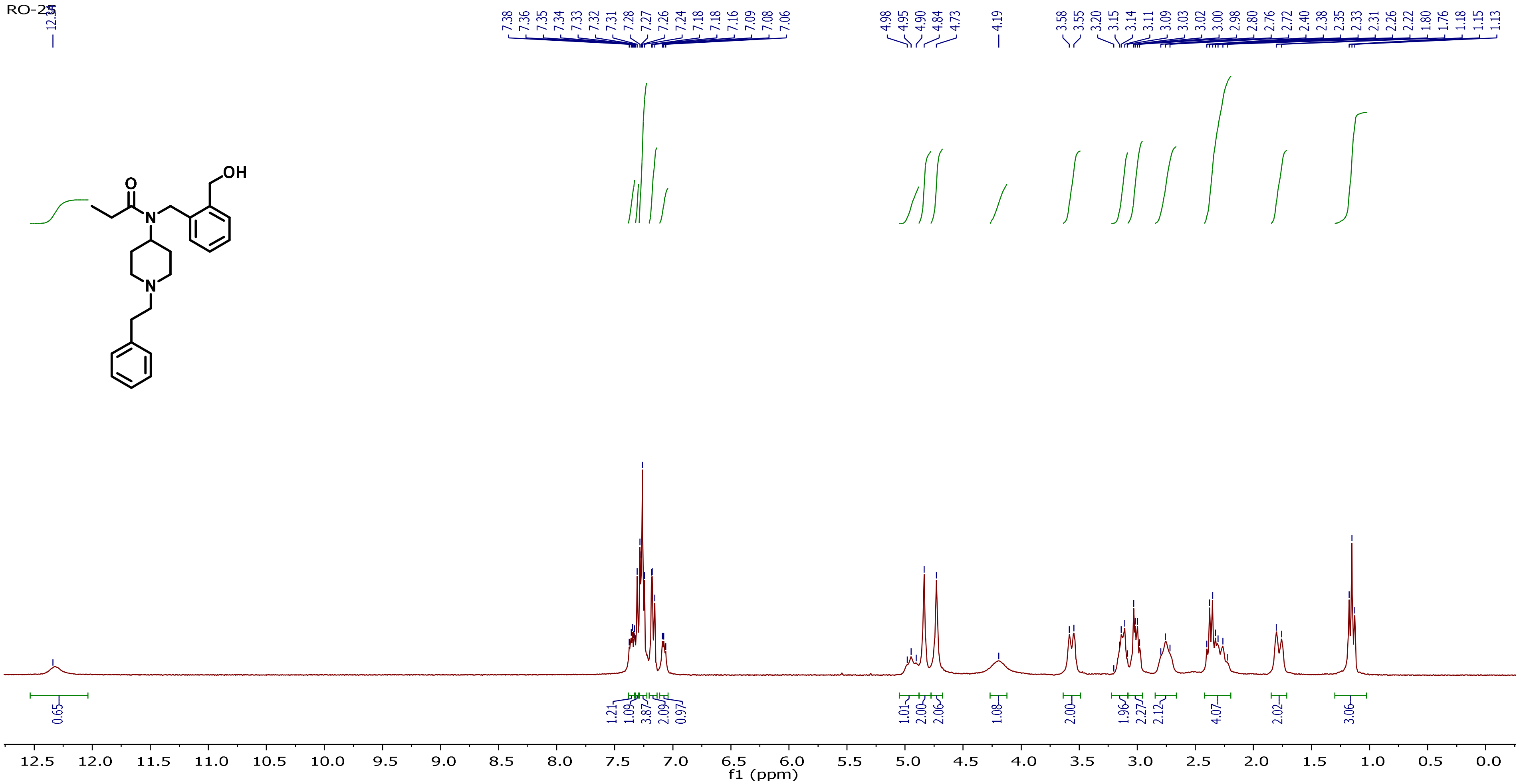

^{13}C NMR of N-(2-(hydroxymethyl)benzyl)-N-(1-phenethylpiperidin-4-yl)propionamide (1) (125 MHz, $CDCl_3$)

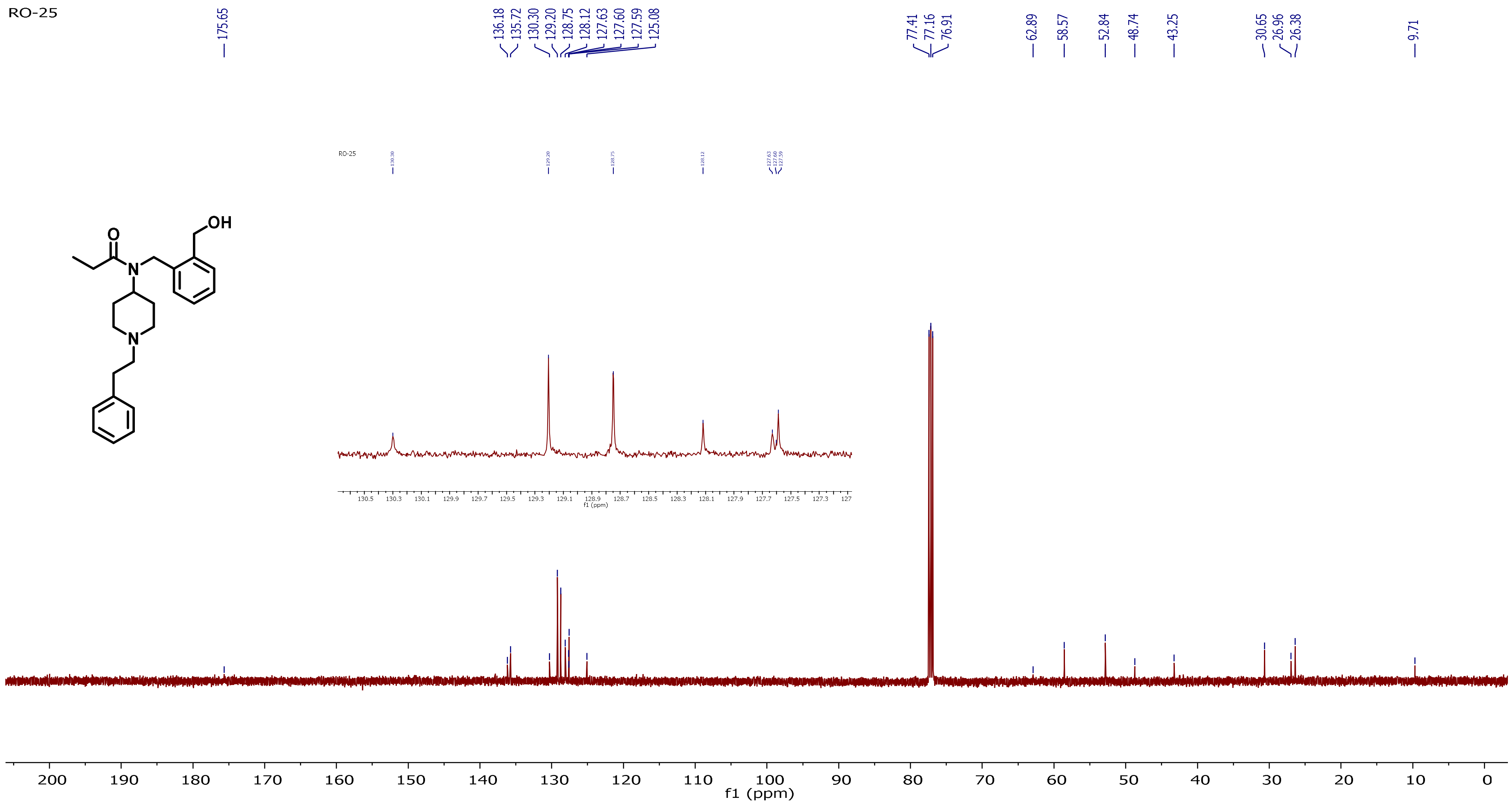

^{1}H NMR of N-(3-(hydroxymethyl)benzyl)-N-(1-phenethylpiperidin-4-yl)propionamide (2/RO76) (400 MHz, $CDCl_3$)

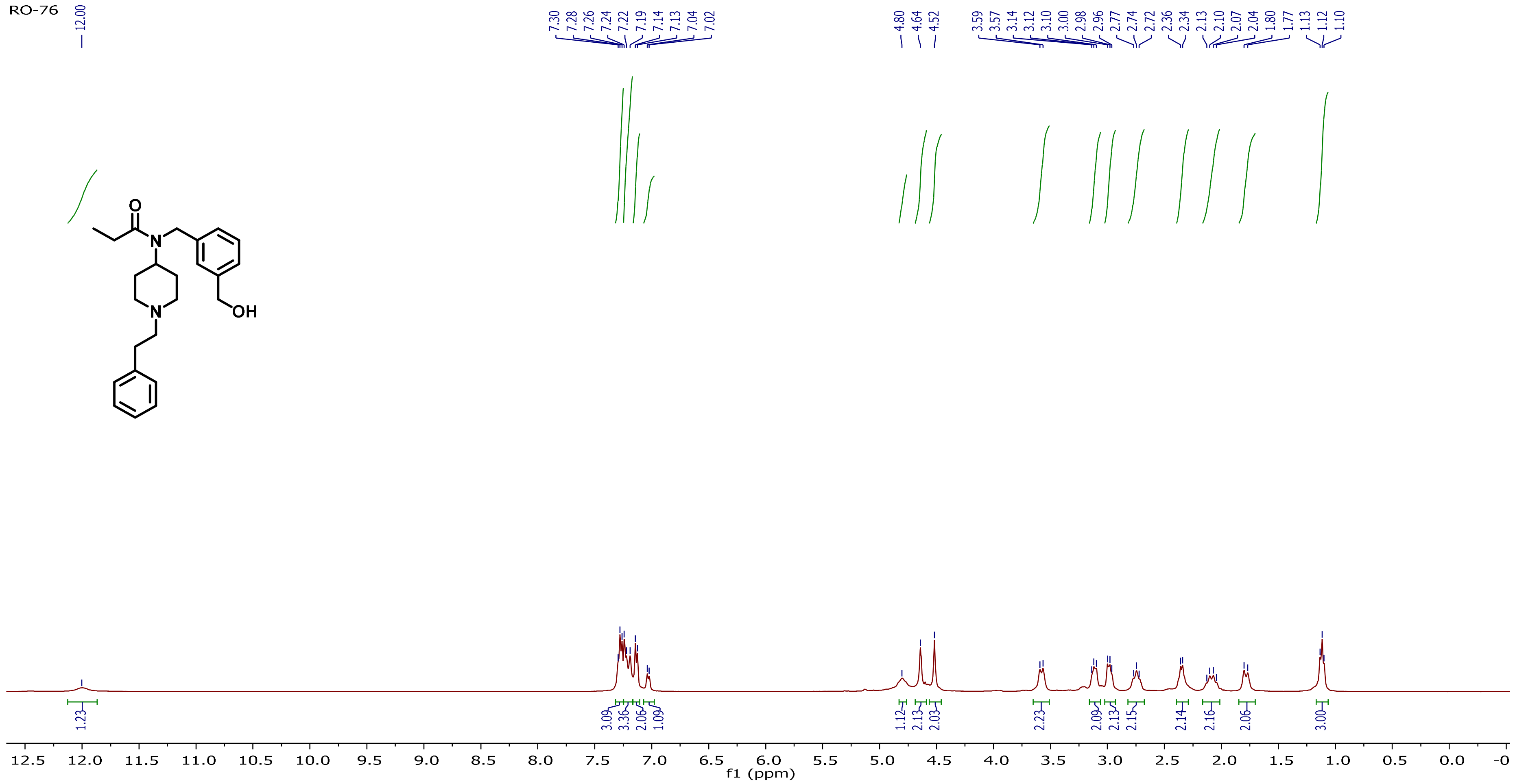

^{13}C NMR of N-(3-(hydroxymethyl)benzyl)-N-(1-phenethylpiperidin-4-yl)propionamide (2/RO76) (100 MHz, $CDCl_3$)

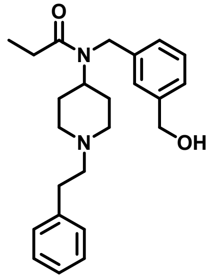

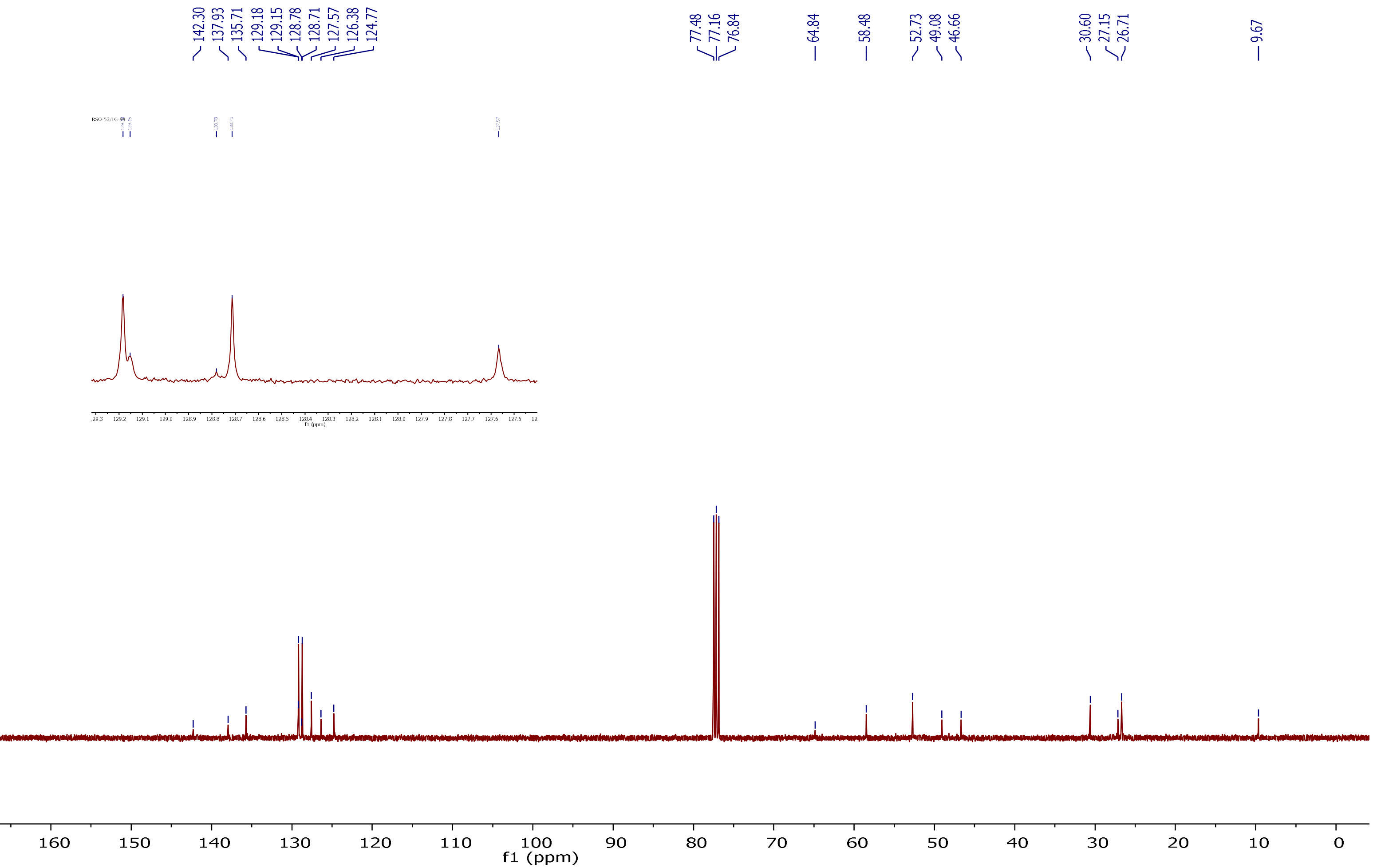

^{1}H NMR of N-(4-(hydroxymethyl)benzyl)-N-(1-phenethylpiperidin-4-yl)propionamide (3) (500 MHz, $CDCl_3$)

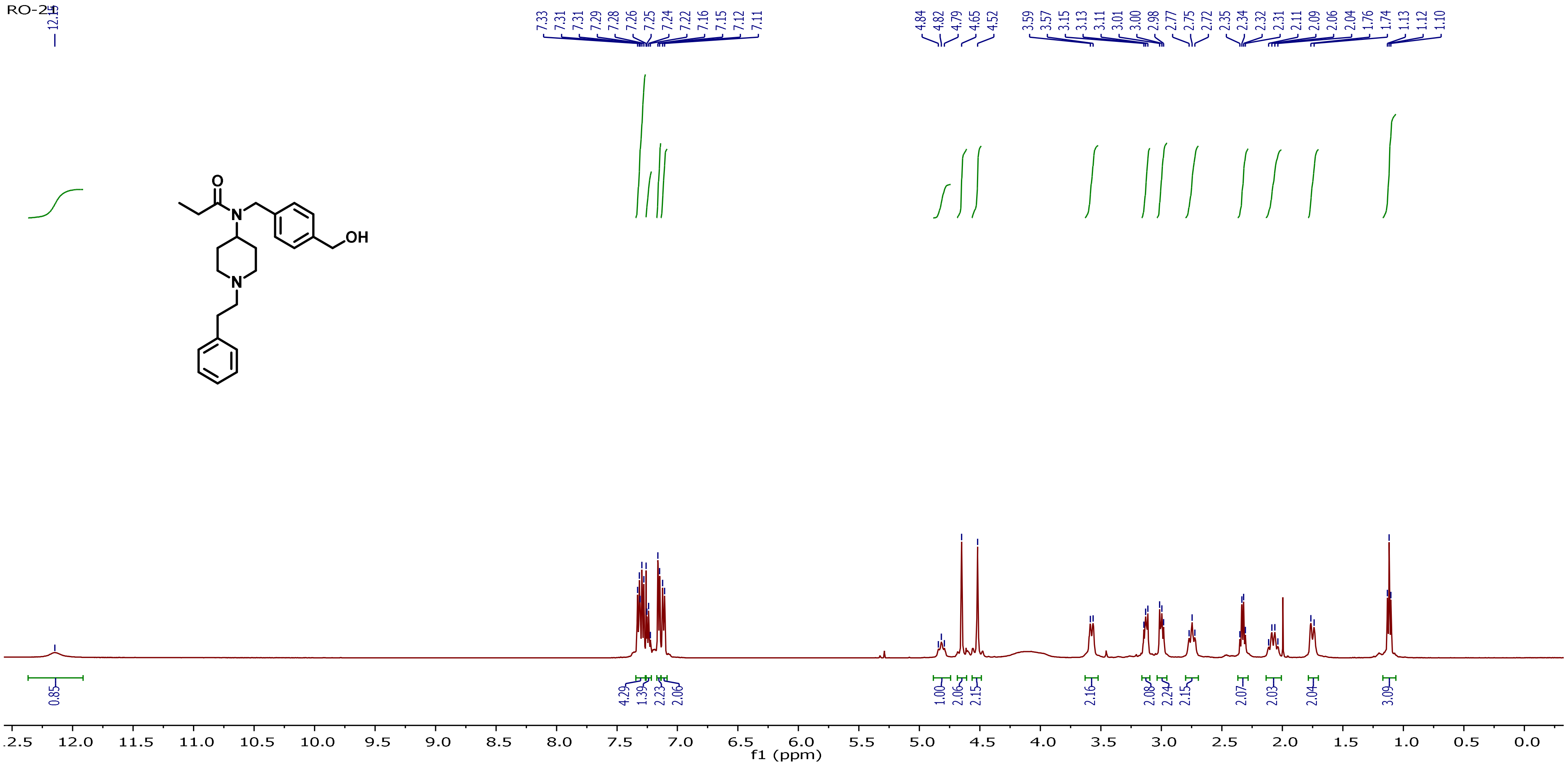

^{13}C NMR of N-(4-(hydroxymethyl)benzyl)-N-(1-phenethylpiperidin-4-yl)propionamide (3) (100 MHz, $CDCl_3$)

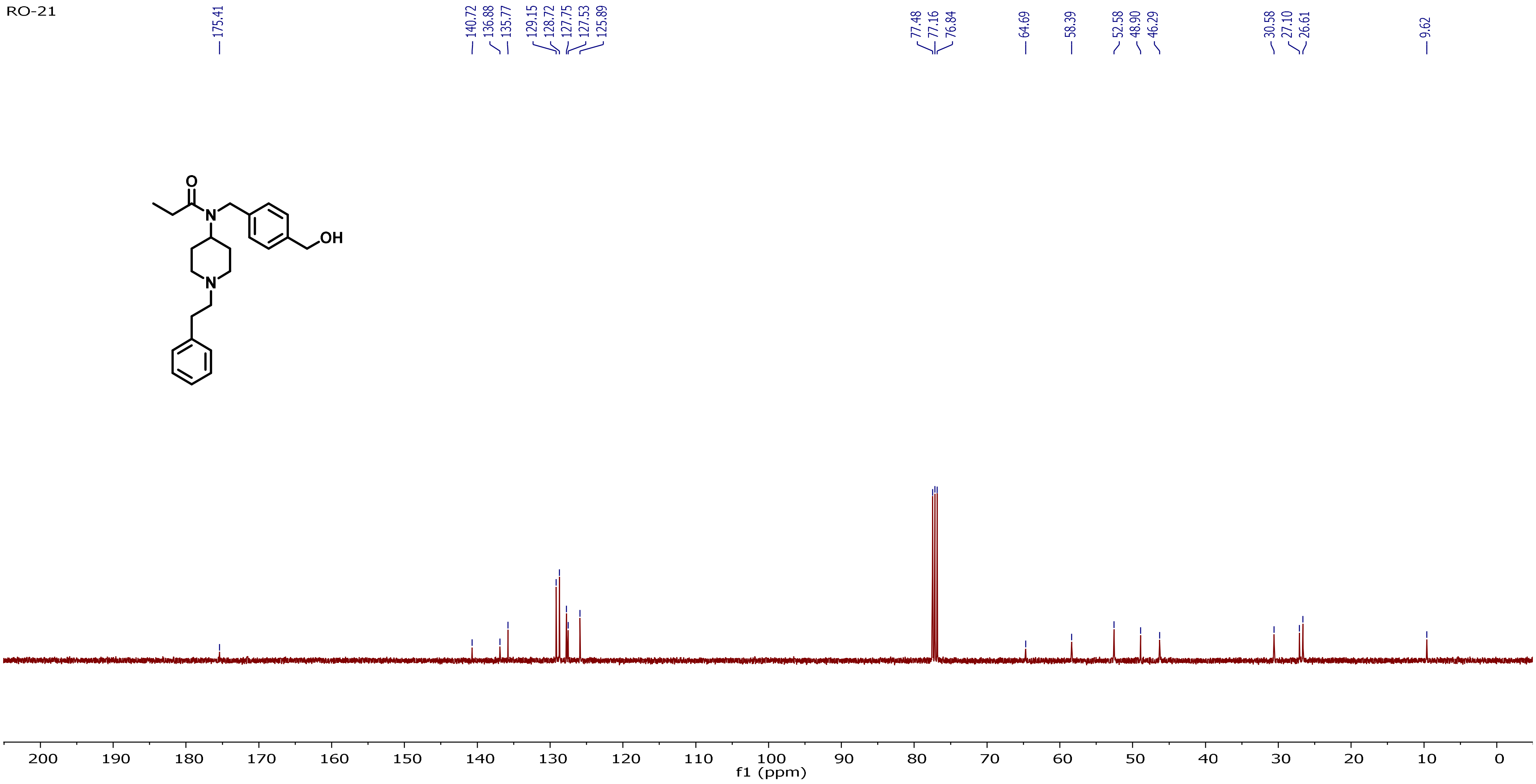

^{1}H NMR of N-(3-hydroxybenzyl)-N-(1-phenethylpiperidin-4-yl)propionamide (4) (400 MHz, $CDCl_3$)

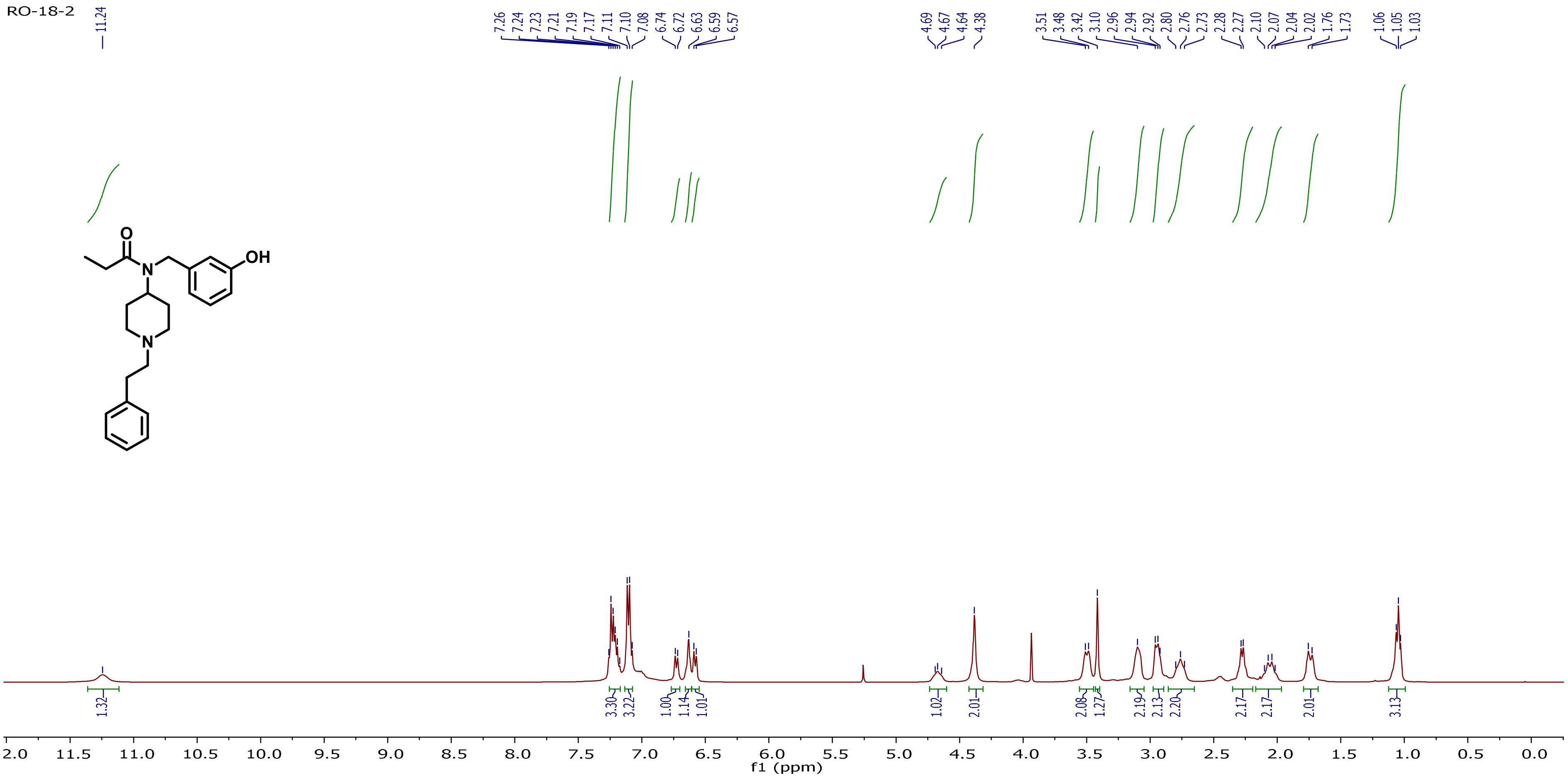

^{13}C NMR of N-(3-hydroxybenzyl)-N-(1-phenethylpiperidin-4-yl)propionamide (4) (100 MHz, $CDCl_3$)

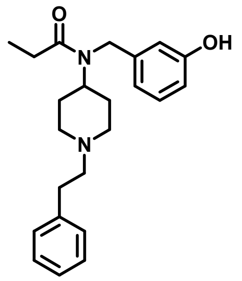

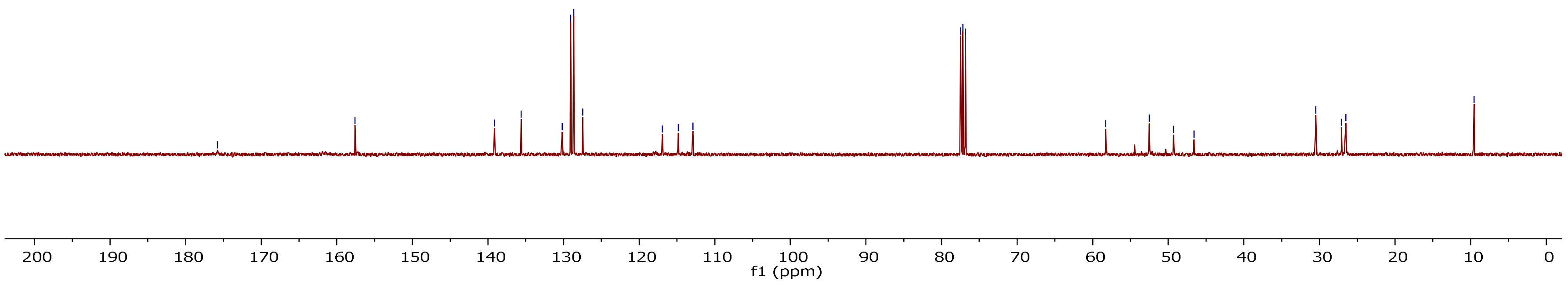

^{1}H NMR of N-(3-(2-hydroxyethyl)phenyl)-N-(1-phenethylpiperidin-4-yl)propionamide (5) (400 MHz, $CDCl_3$)

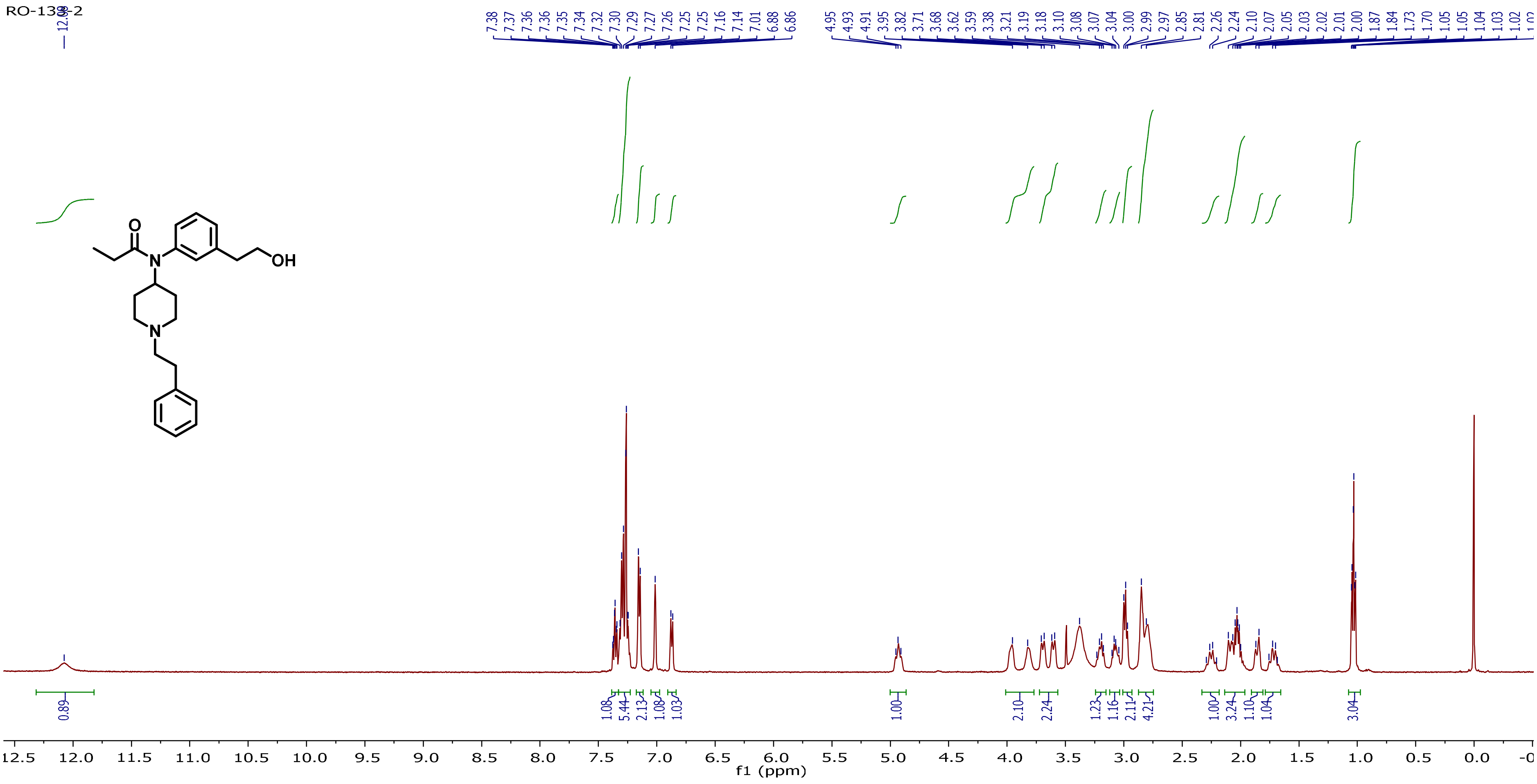

^{13}C NMR of N-(3-(2-hydroxyethyl)phenyl)-N-(1-phenethylpiperidin-4-yl)propionamide (5) (100 MHz, $CDCl_3$)

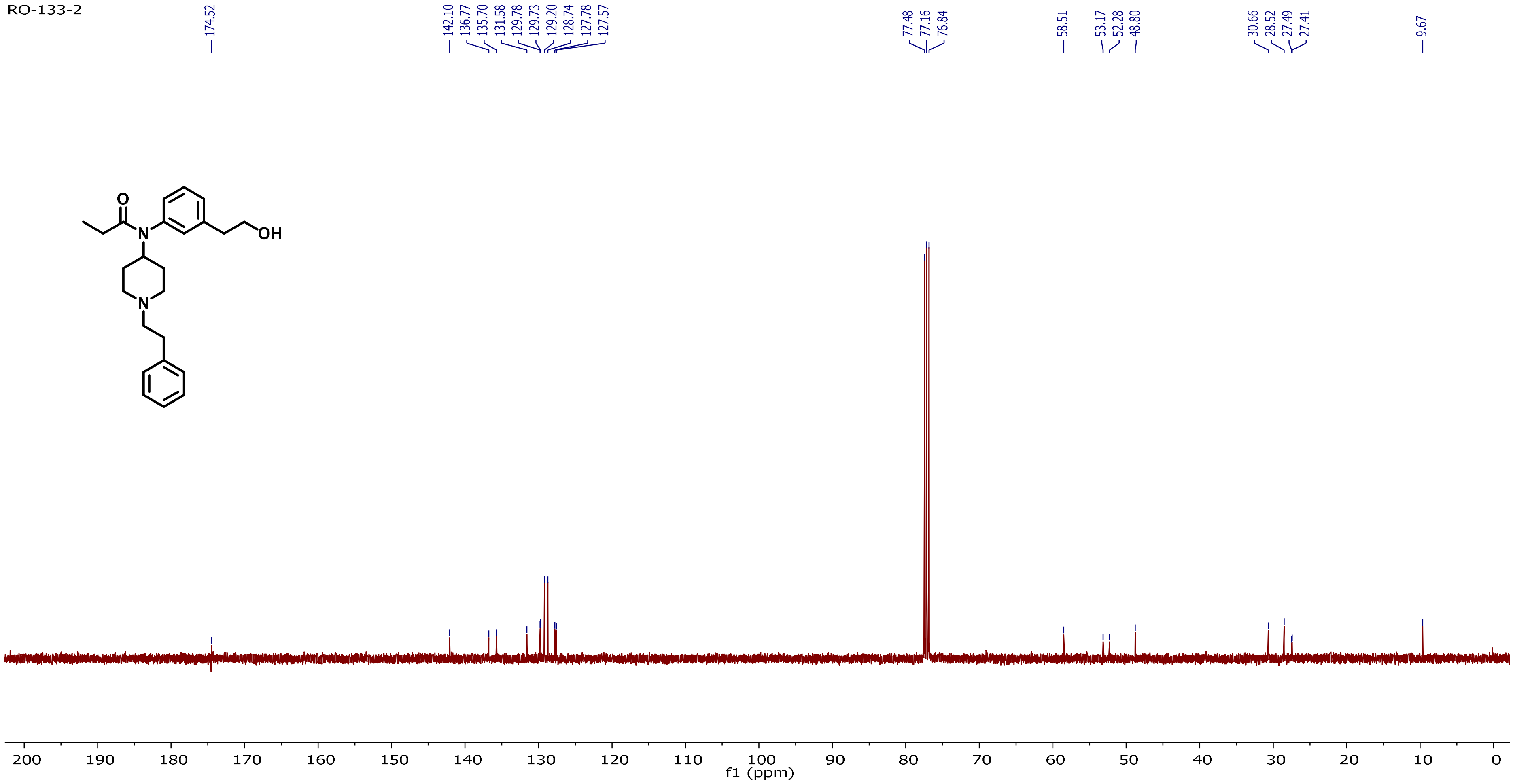

^{1}H NMR of N-(3-(2-hydroxyethyl)benzyl)-N-(1-phenethylpiperidin-4-yl)propionamide (6) (400 MHz, $CDCl_3$)

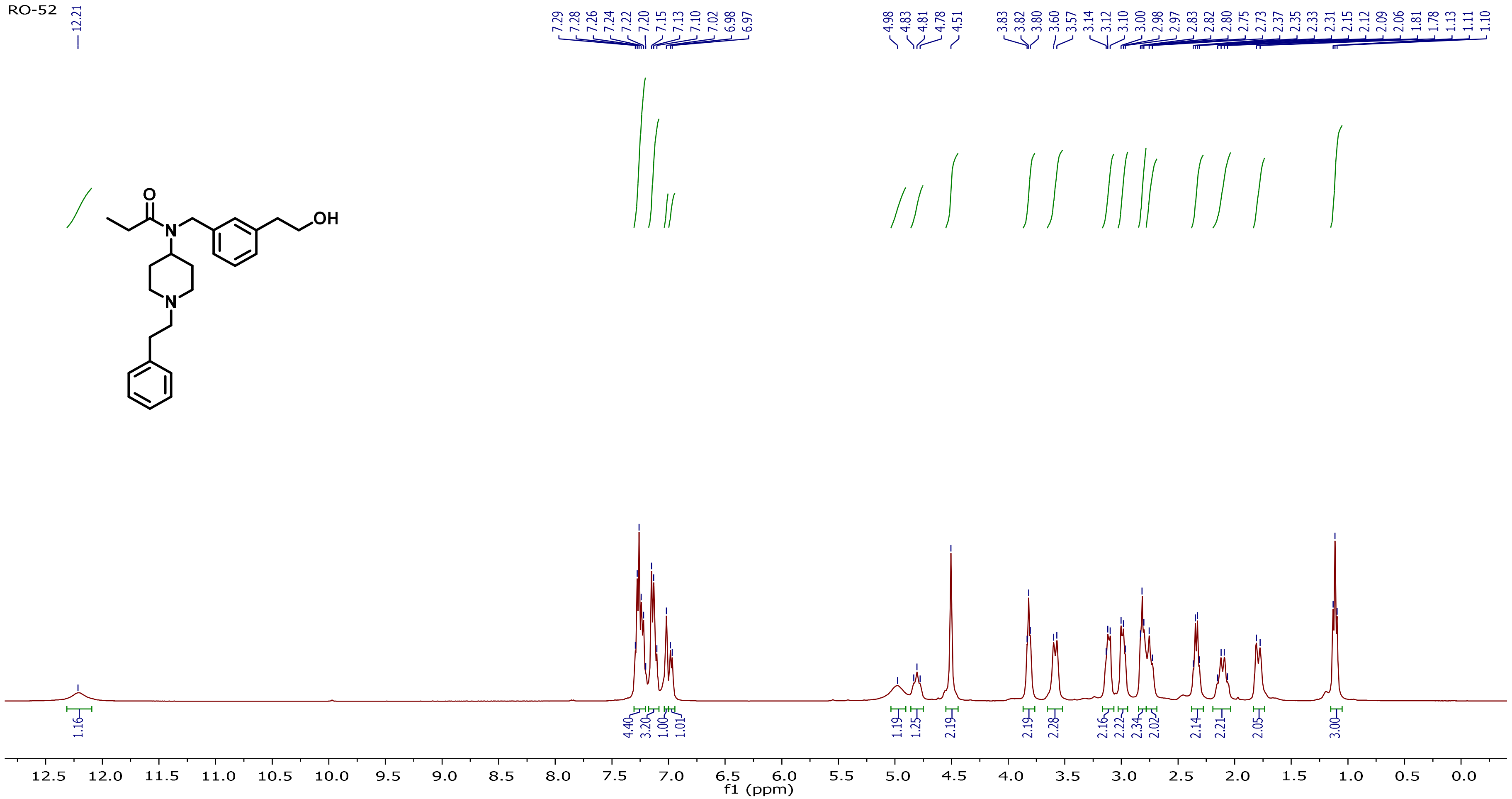

^{13}C NMR of N-(3-(2-hydroxyethyl)benzyl)-N-(1-phenethylpiperidin-4-yl)propionamide (6) (100 MHz, $CDCl_3$)

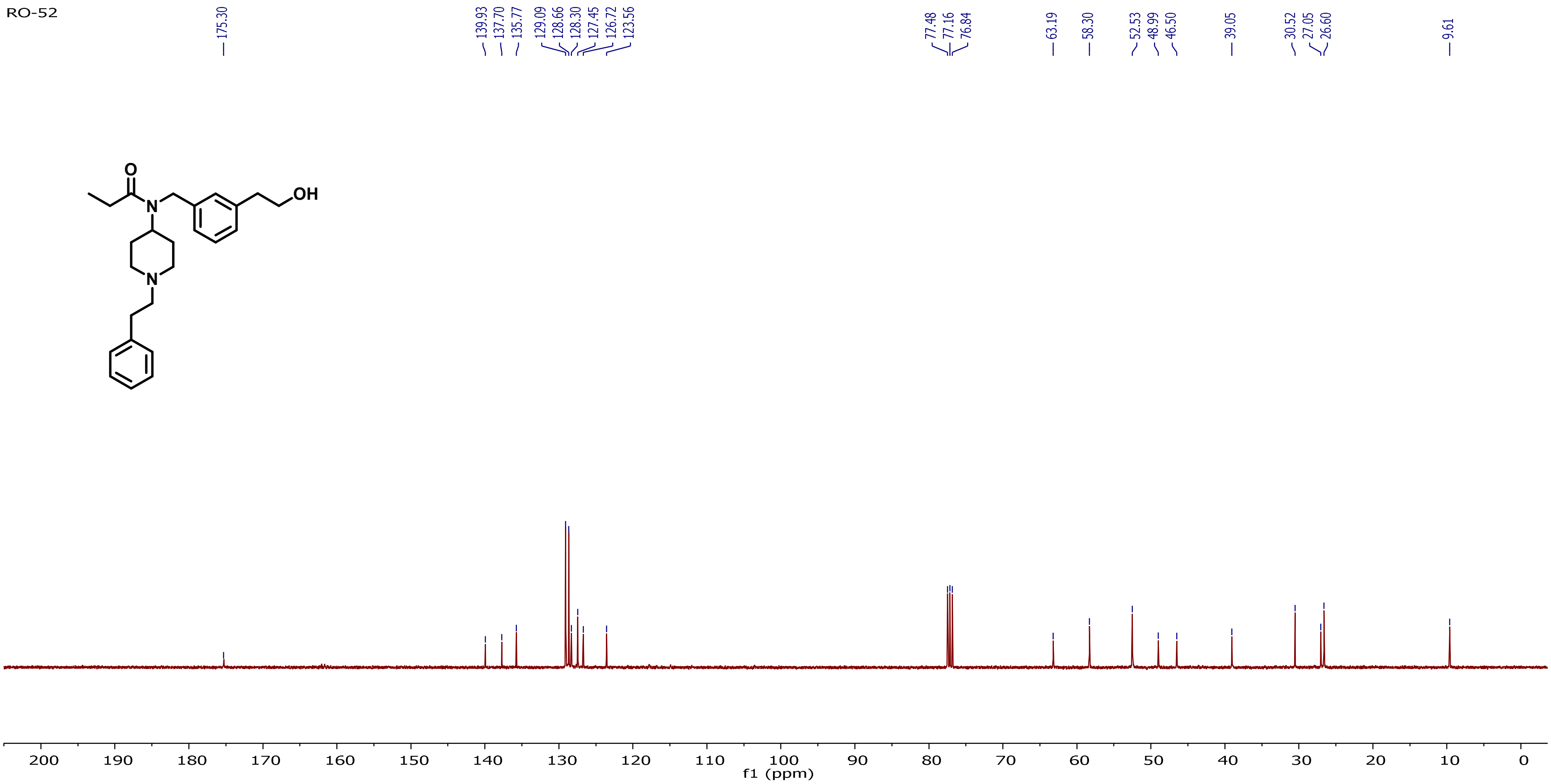

^{1}H NMR of N-(3-(methoxymethyl)benzyl)-N-(1-phenethylpiperidin-4-yl)propionamide (7) (400 MHz, $CDCl_3$)

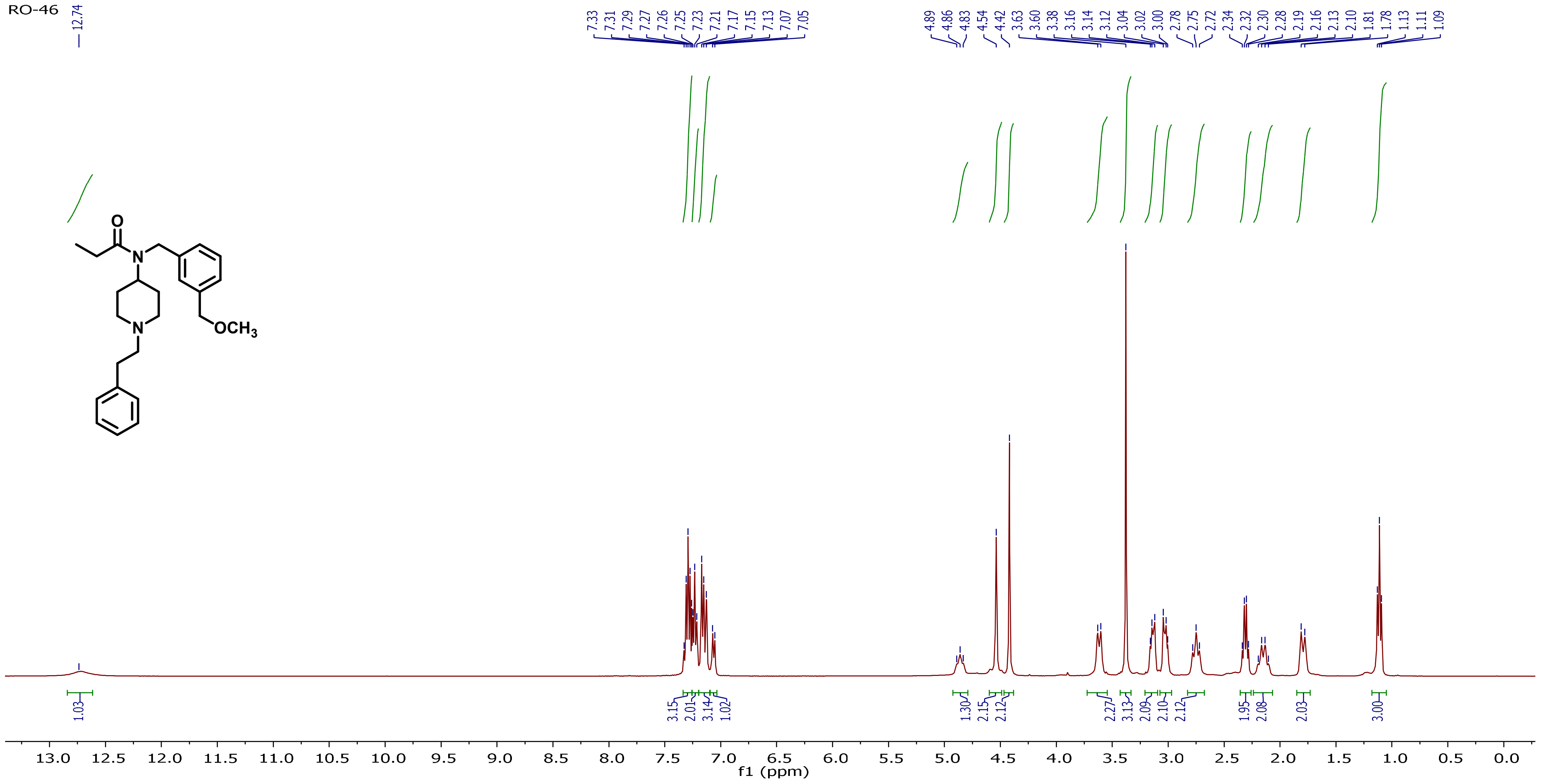

^{13}C NMR of N-(3-(methoxymethyl)benzyl)-N-(1-phenethylpiperidin-4-yl)propionamide (7) (100 MHz, $CDCl_3$)

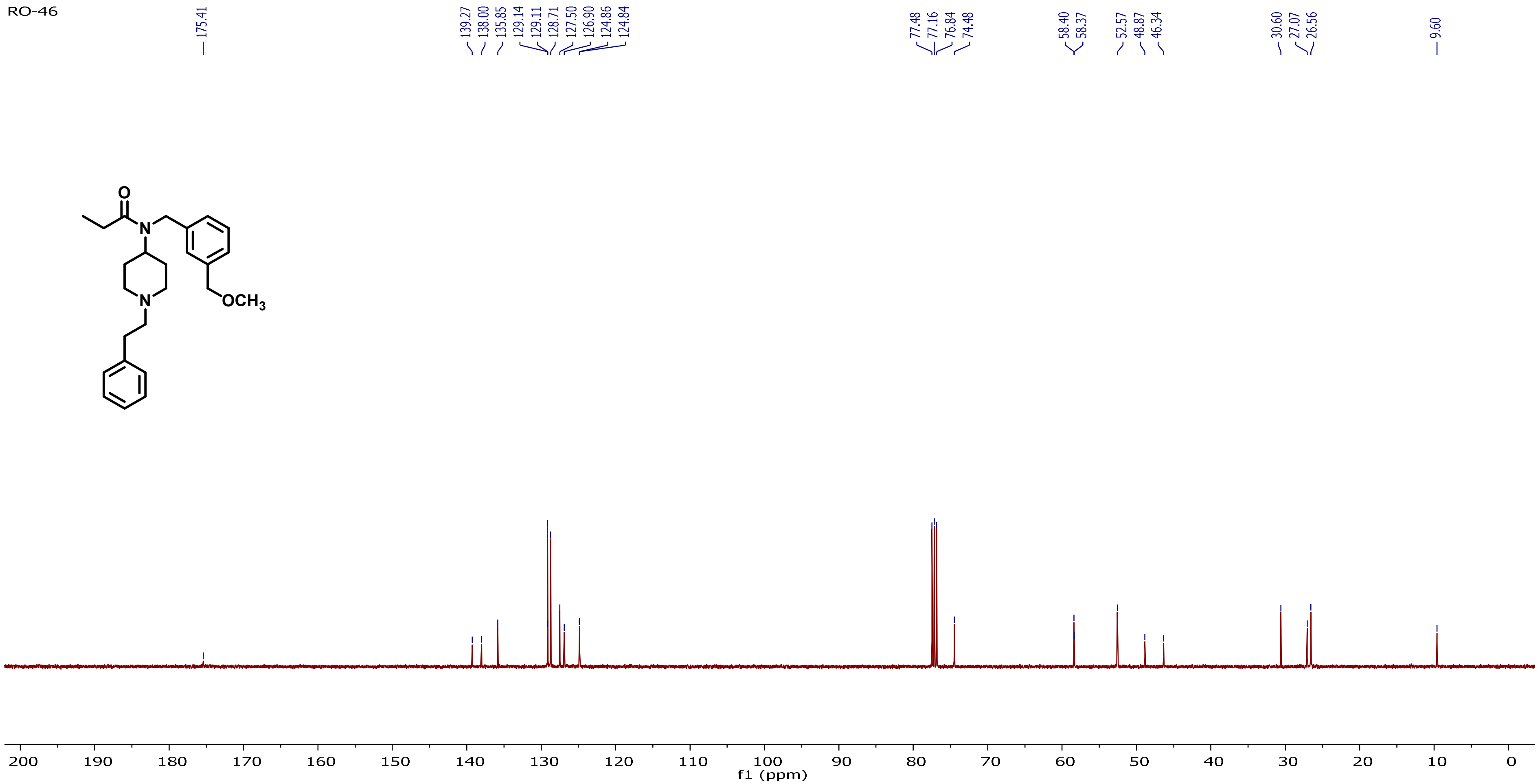

^{1}H NMR of Methyl 3-((N-(1-phenethylpiperidin-4-yl)propionamido)methyl)benzoate (8/RO-152) (400 MHz, $CDCl_3$)

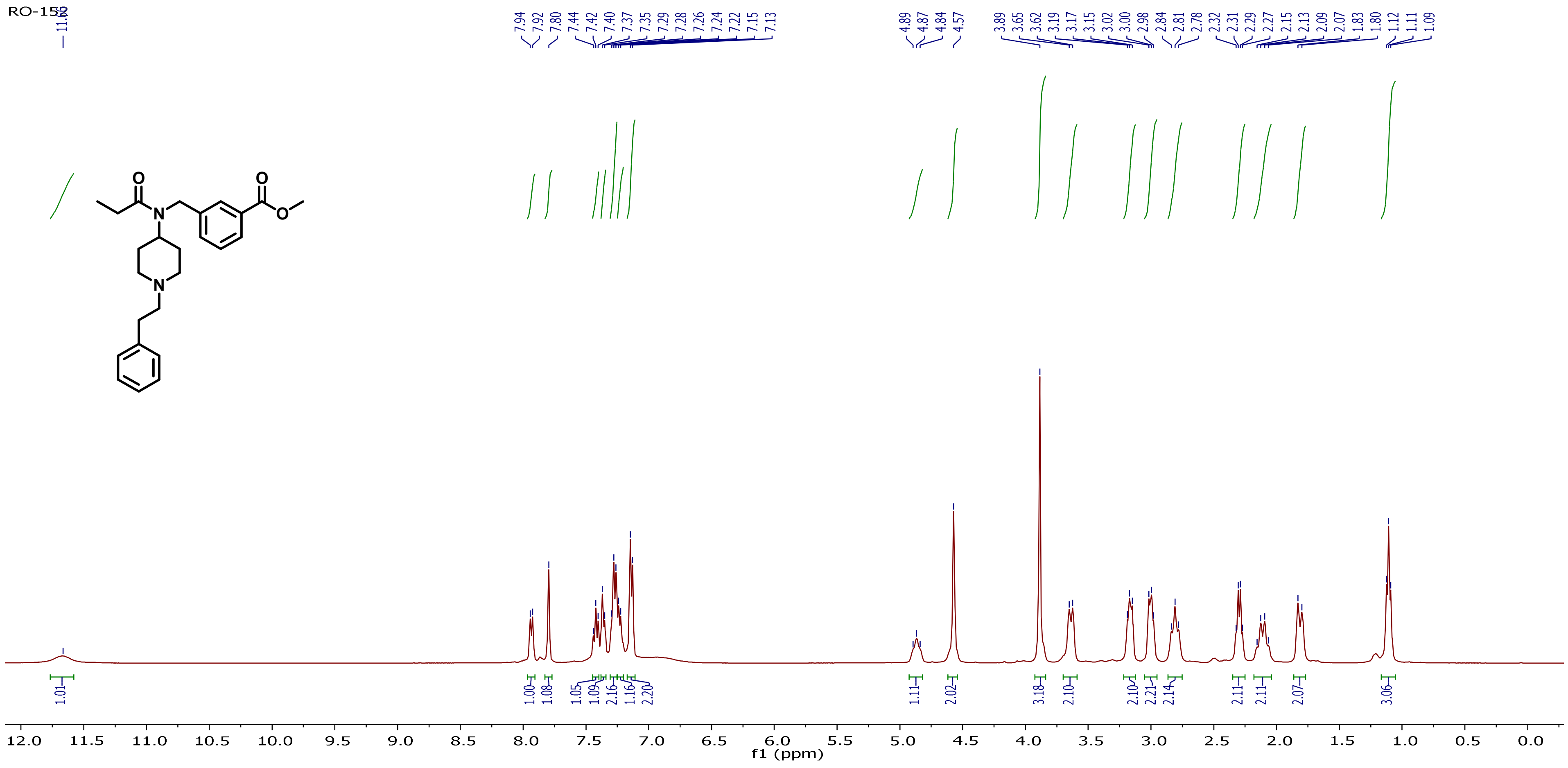

^{13}C NMR of Methyl 3-((N-(1-phenethylpiperidin-4-yl)propionamido)methyl)benzoate (8/RO-152) (100 MHz, $CDCl_3$)

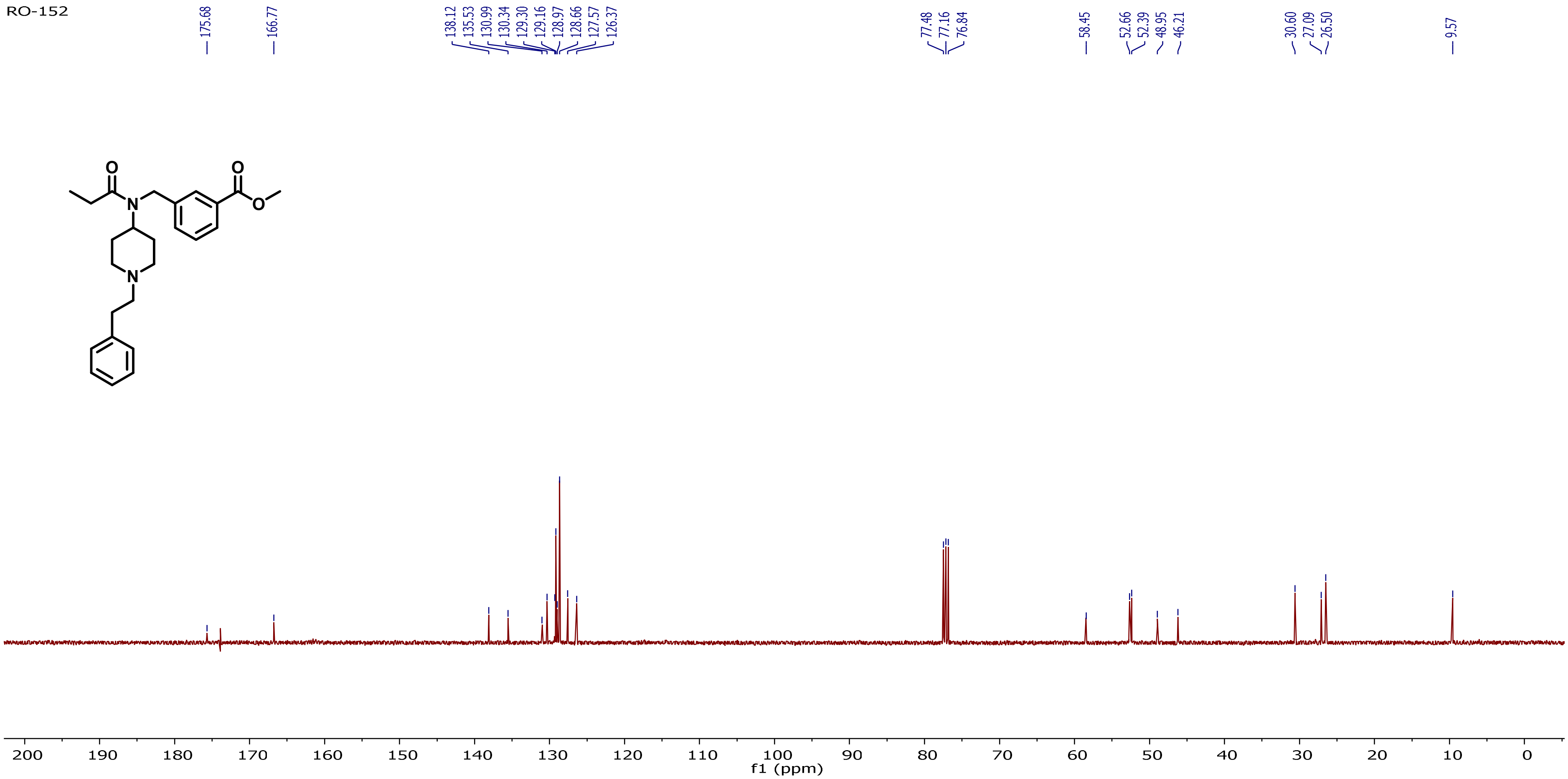

^{1}H NMR of N-(3-ethoxybenzyl)-N-(1-phenethylpiperidin-4-yl)propionamide (9) (400 MHz, $CDCl_3$)

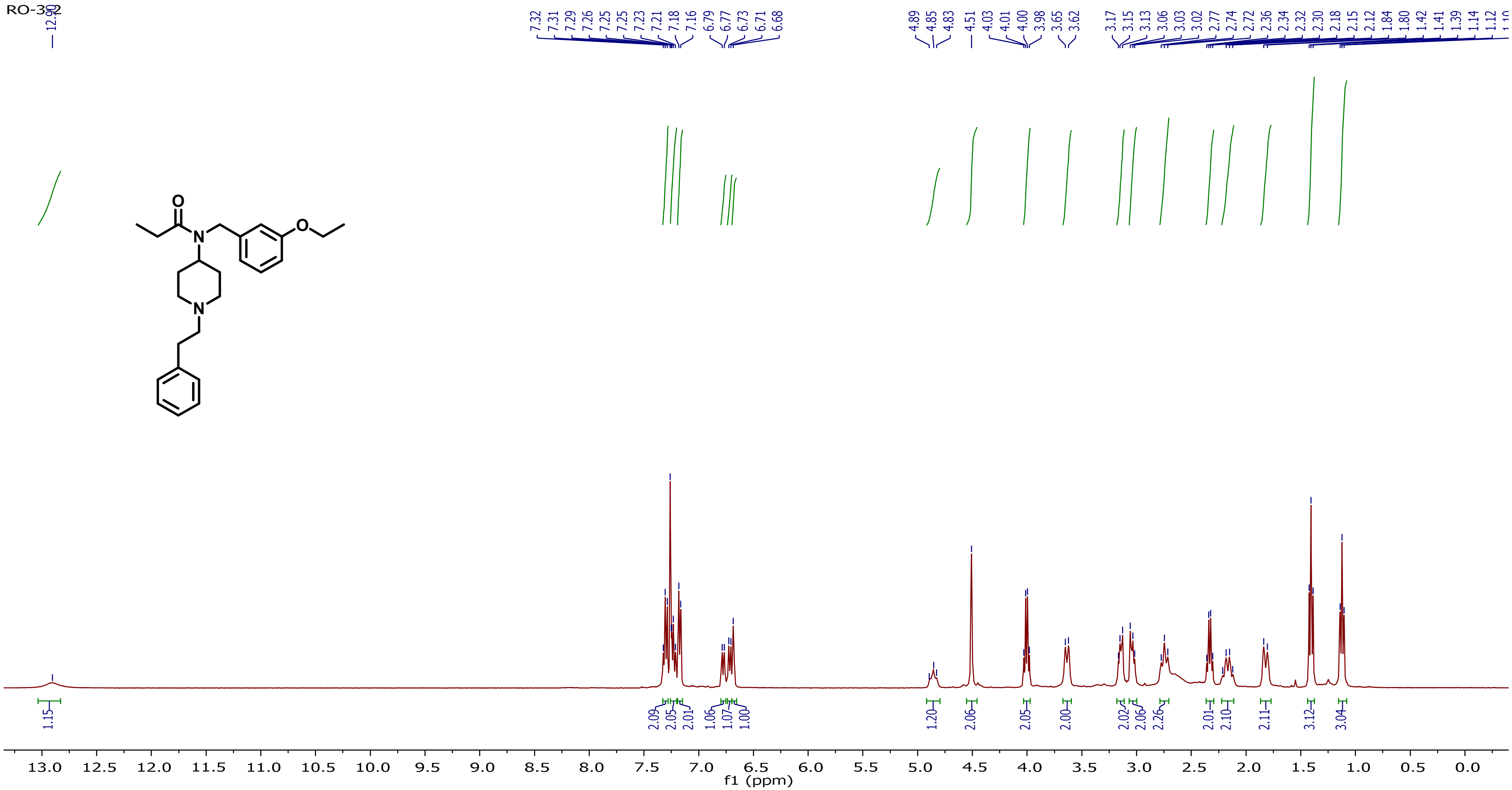

^{13}C NMR of N-(3-ethoxybenzyl)-N-(1-phenethylpiperidin-4-yl)propionamide (9) (100 MHz, $CDCl_3$)

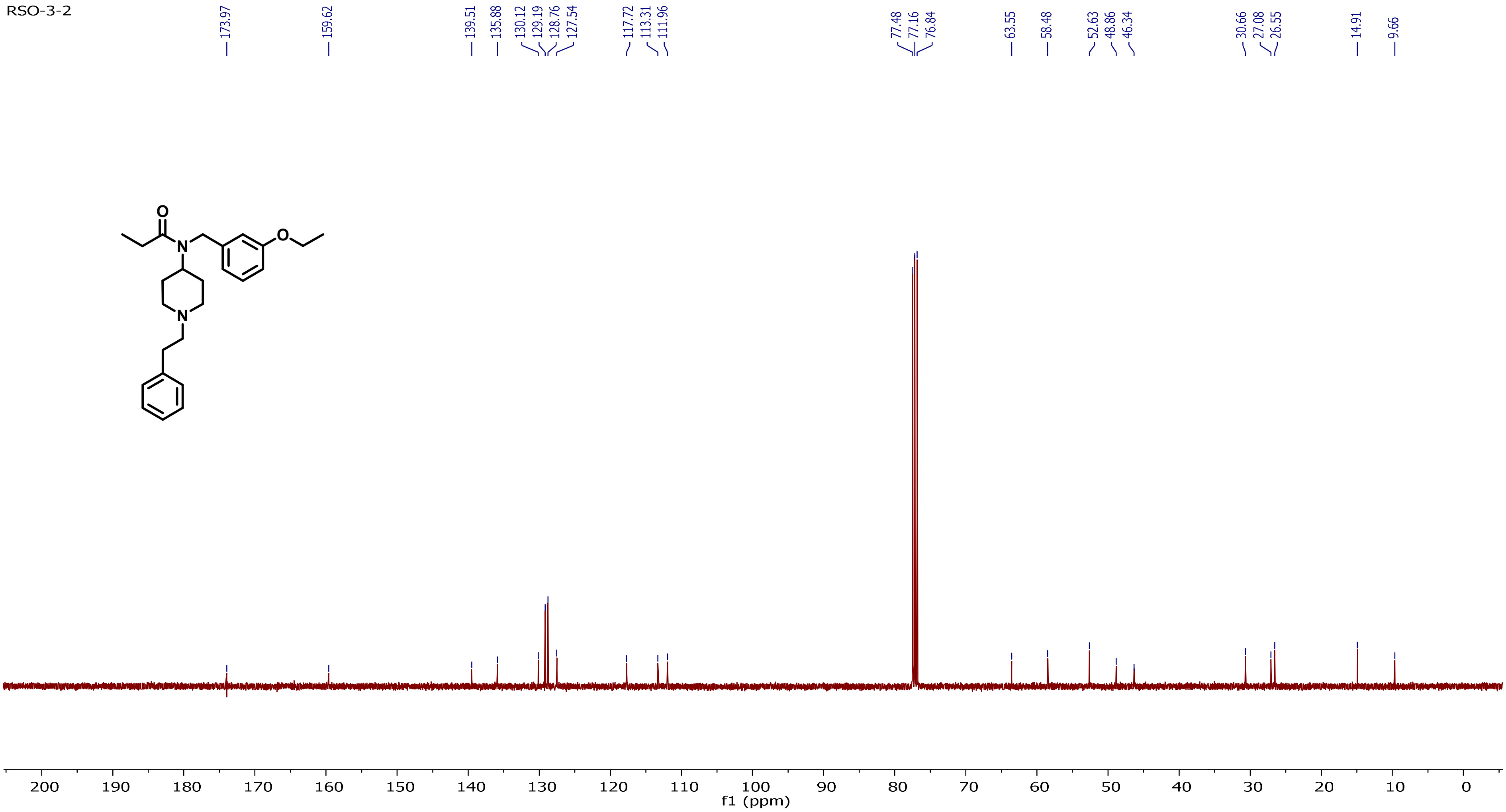

^{1}H NMR of 3-((N-(1-phenethylpiperidin-4-yl)propionamido)methyl)benzoic acid (10) (400 MHz, $CDCl_3$)

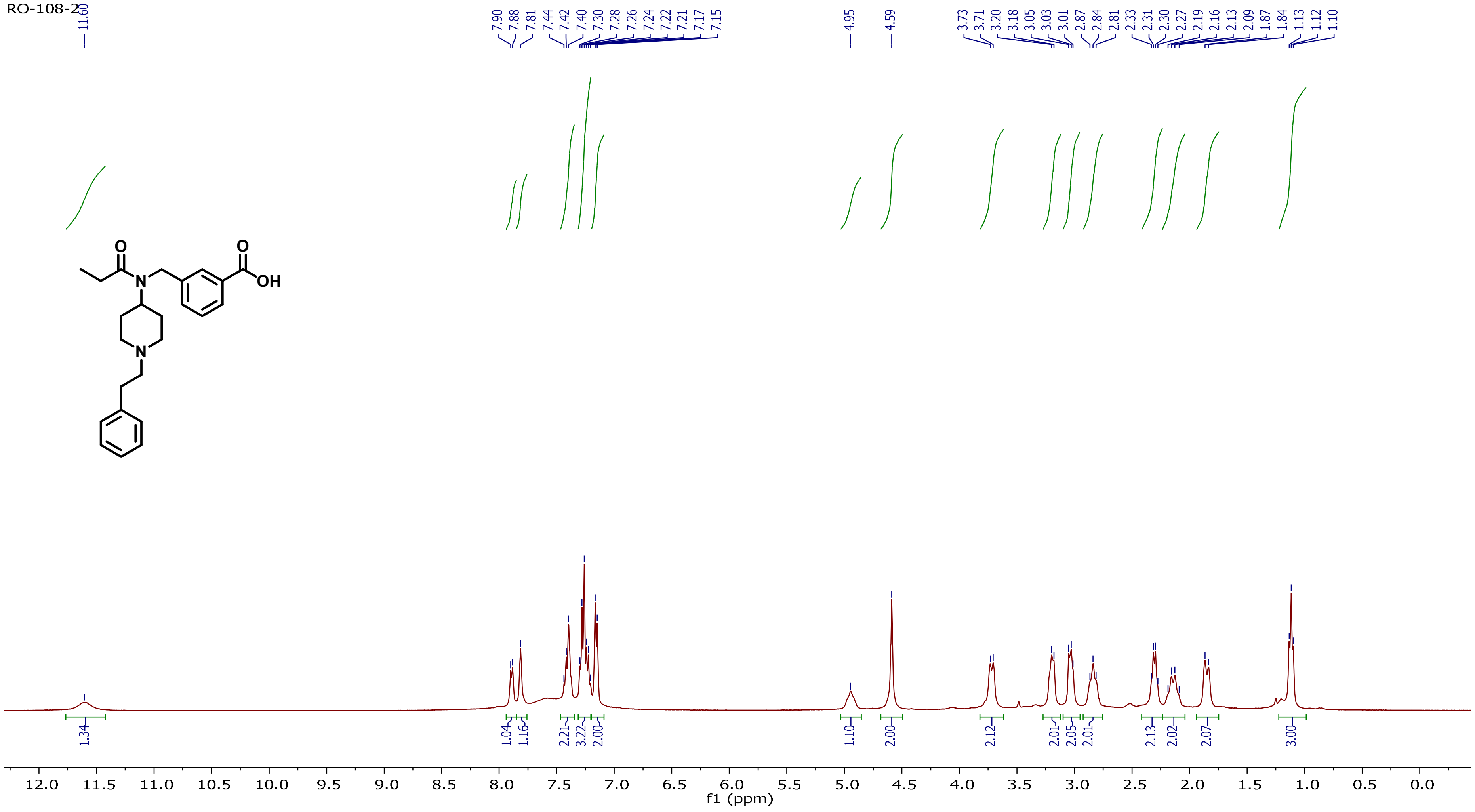

^{13}C NMR of 3-((N-(1-phenethylpiperidin-4-yl)propionamido)methyl)benzoic acid (10) (100 MHz, $CDCl_3$)

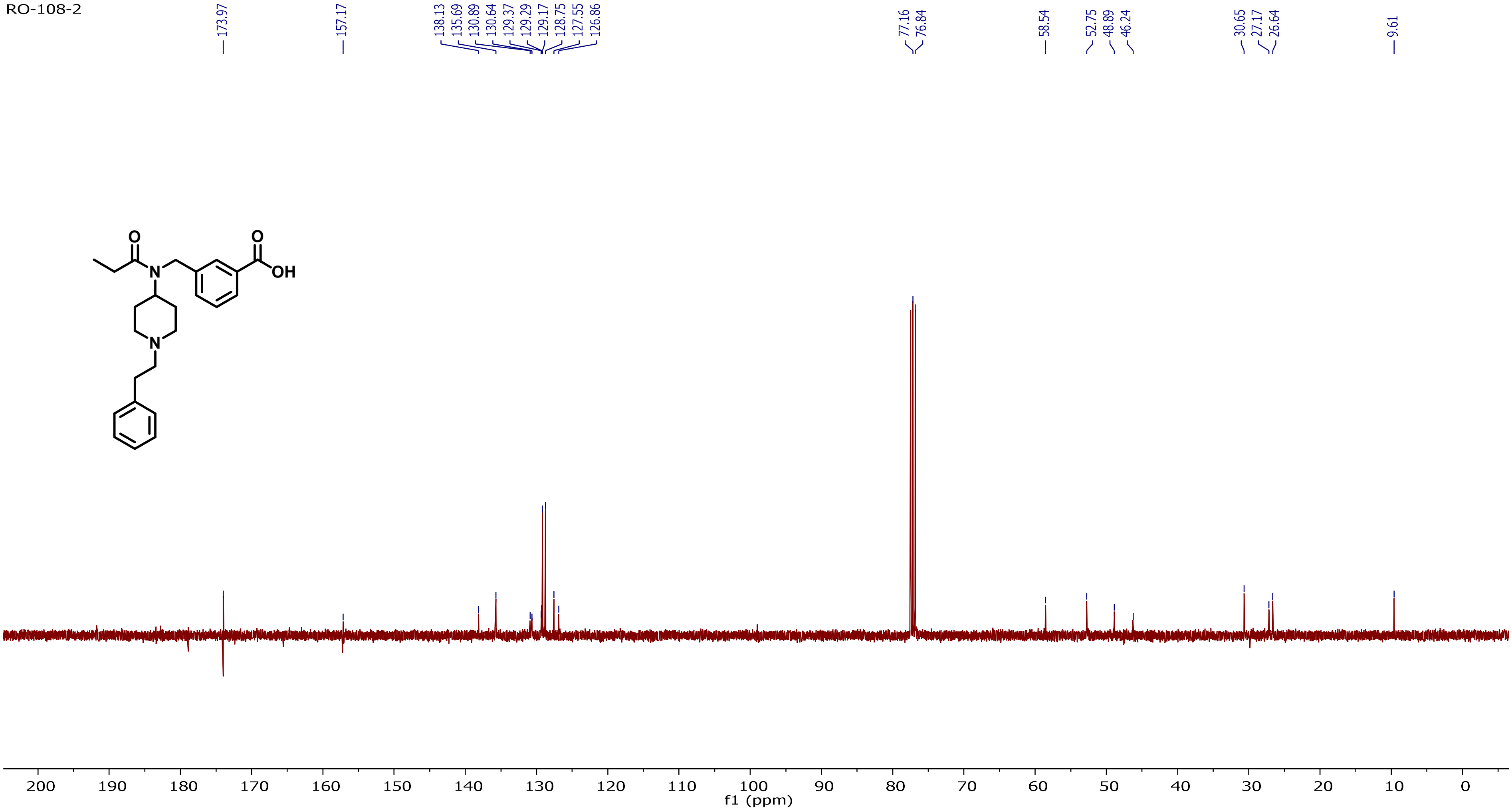

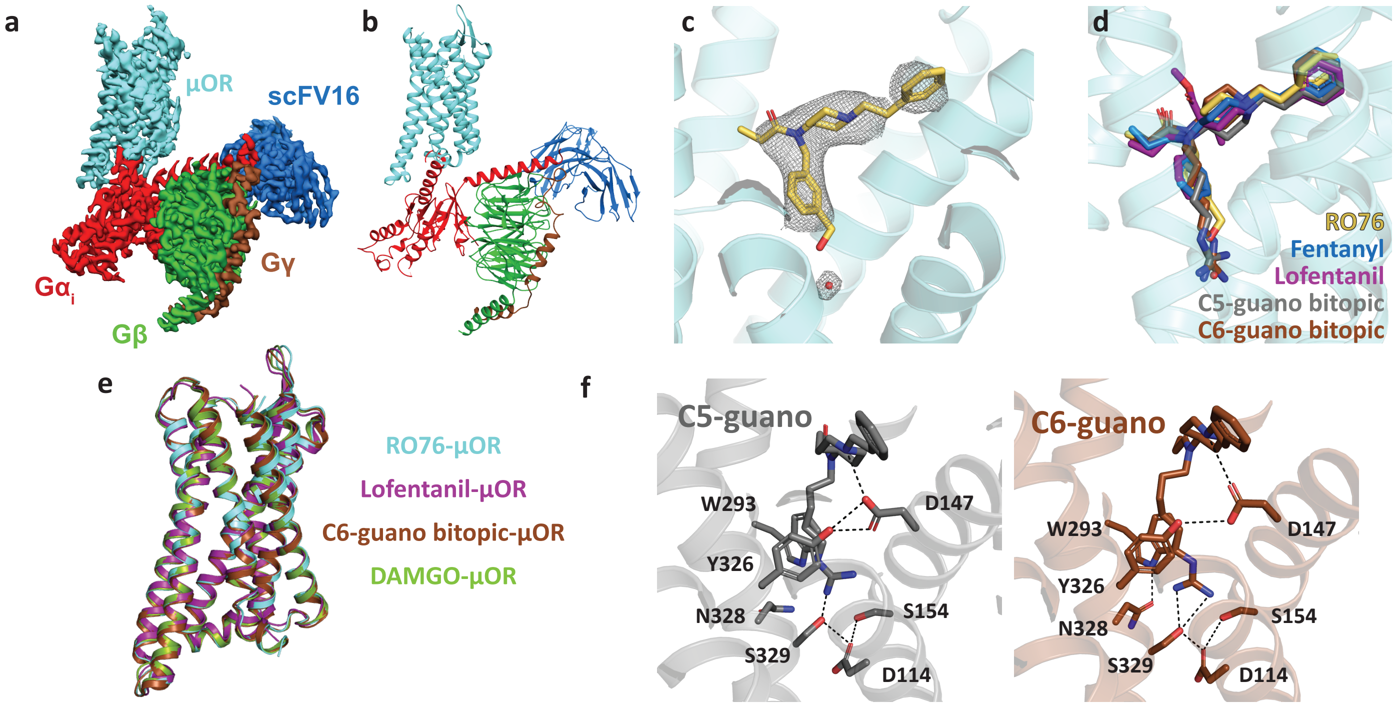

a
μOR
scFV16
Gγ
Gαi
Gβ
b
c
d
RO76
Fentanyl
Lofentanil
C5-guano bitopic
C6-guano bitopic
e
RO76-μOR
Lofentanil-μOR
C6-guano bitopic-μOR
DAMGO-μOR
f
C5-guano
W293
D147
Y326
N328
S154
S329
D114
C6-guano
W293
D147
Y326
N328
S154
S329
D114

Figure S1: Cryo-EM structure of RO76 bound to μOR-G_{i1}-scFv16 complex. (a) cryoEM map of **RO76** bound μOR–G_{i1}-scFv16 complex. **(b)** Fitted model coordinates of **RO76** bound μOR–G_{i1}-scFv16 complex. **(c)** cryoEM density of **RO76** as well as the water molecule underneath. The map is contoured at 9.8 σ. **(d)** Superposition of the **RO76** binding pose with fentanyl/fentanyl analogs. **(e)** Superposition of the overall conformation of **RO76/lofentanil/C6-guano bitopic/DAMGO** bound μOR. **(f)** Zoom-in view revealing the detailed interaction between **C5/C6-guano bitopic ligands** with orthosteric pocket residues.

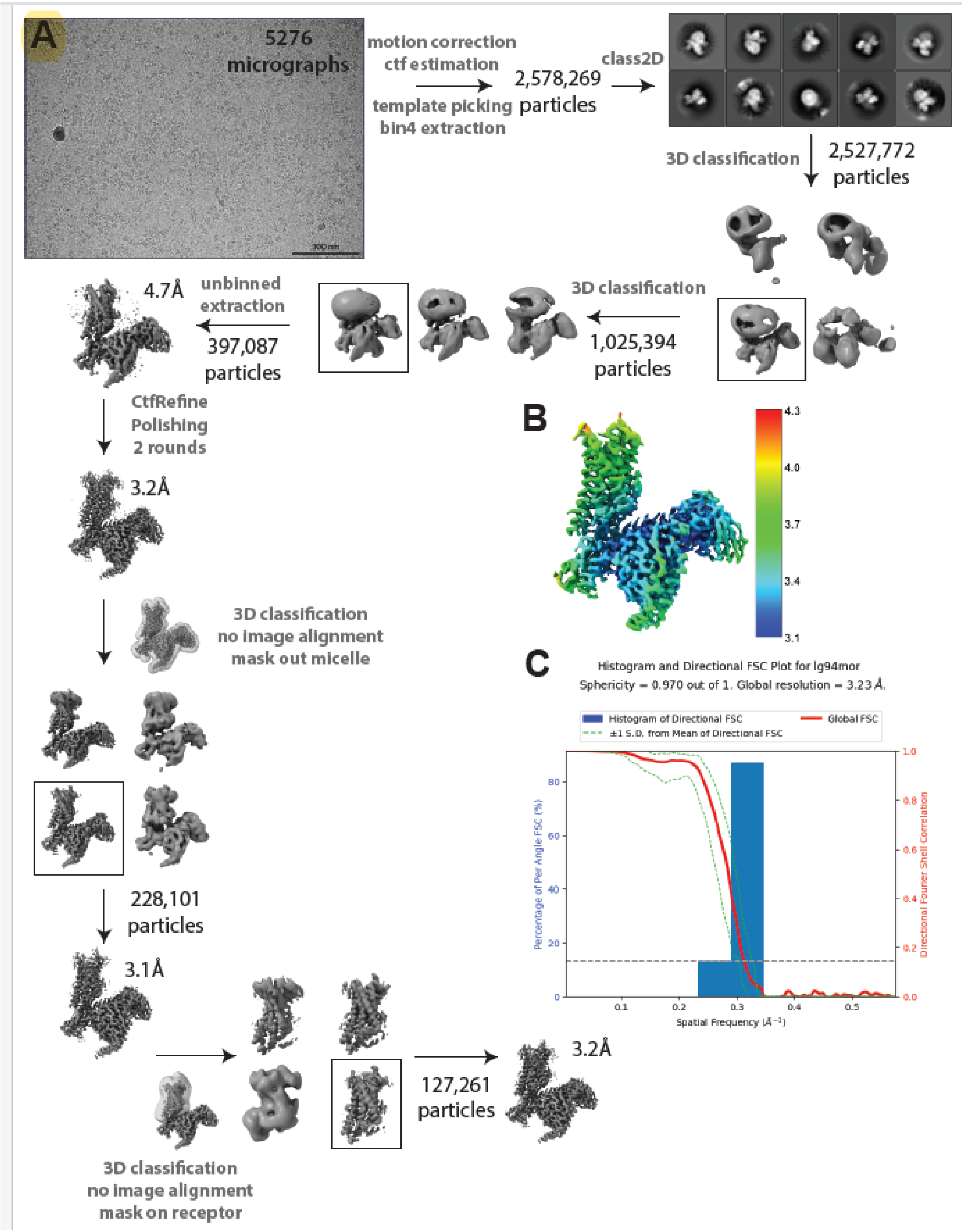

Figure S2: Cryo-EM data processing work-flows. (a) Representative micrographs, 2D classes, 3D classes and data processing procedures for **RO76** bound μOR–G_i-scFv16 complex. **(b)** Local resolution map of **RO76** bound μOR–G_i structures. **(c)** Gold-standard FSC curves for **RO76** bound μOR–G_i structures. Overall resolution is 3.23 Å for **RO76** bound μOR–G_i-scFv16 using the gold Standard FSC = 0.143

	RO76 bound μOR-Gi1-scFv16 complex. (EMDB-44812) (PDB 9BQJ)
**Data collection and processing**	
Magnification	130,000
Voltage (kV)	300
Electron exposure (e–/Å^2)	54.45
Defocus range (μm)	0.7-2
Pixel size (Å)	0.8677
Symmetry imposed	C1
Initial particle images (no.)	2578269
Final particle images (no.)	127261
Map resolution (Å)	3.2
FSC threshold	0.143
Map resolution range (Å)	3.1-4.3
**Refinement**	
Initial model used (PDB code)	7U2L
Model resolution (Å)	3.3
FSC threshold	0.5
Model resolution range (Å)	3.3-27.8
Map sharpening *B* factor (Å^2)	-89.84
Model composition.	
Non-hydrogen atoms	8524
Protein residues	1122
Ligands	1
B factors (Å^2)	
Protein	79.01
Ligand	101.43
R.m.s. deviations	
Bond lengths (Å)	0.003
Bond angles (°)	0.547
Validation	
MolProbity score	1.44
Clashscore	5.67
Poor rotamers (%)	0.23
Ramachandran plot	
Favored (%)	97.29
Allowed (%)	2.62
Disallowed (%)	0.09

Figure S3: Data collection, refinement, and model statistic of cryo-EM structure

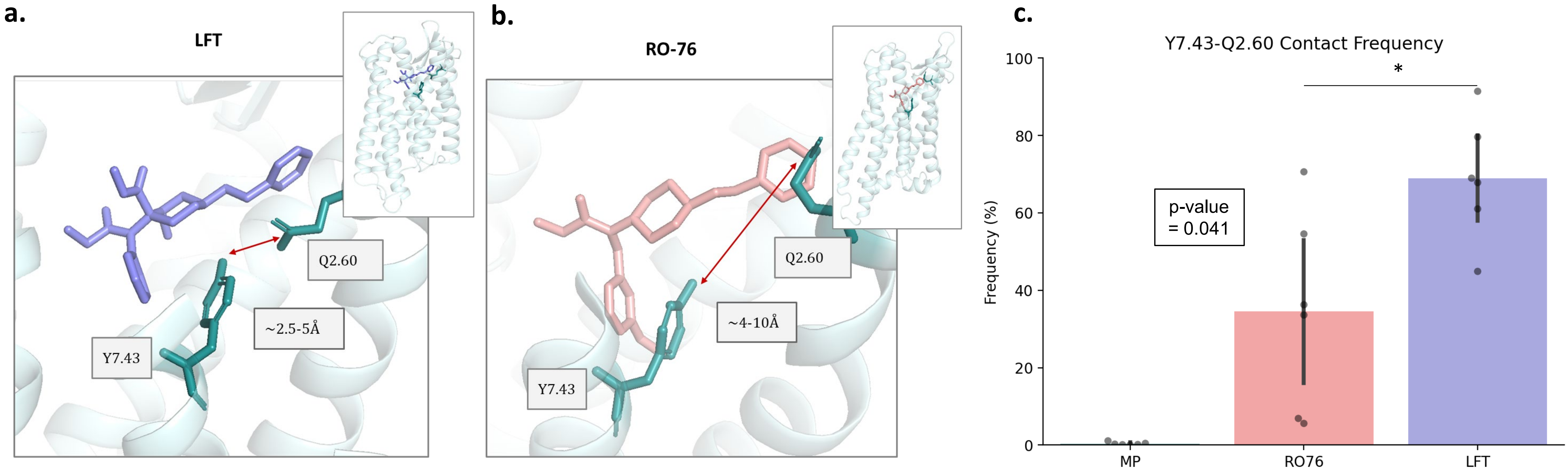

Figure S4: RO76 Demonstrates Lower $Y^{7.43}$-$Q^{2.60}$ H-Bond Frequency vs LFT

a. Representative frame of LFT interactions in simulation. All relevant residues are shown in licorice representation and colored teal. LFT is colored periwinkle. The typical distance between the side chain hydroxyl on $Y^{7.43}$ and the side chain nitrogen on $Q^{2.60}$ was about 2.5-5 Å.

b. Representative frame of **RO76** interactions in simulation. All relevant residues are shown in licorice representation and colored teal. **RO76** is colored in salmon. The distance between the side chain hydroxyl on $Y^{7.43}$ and the side chain nitrogen on $Q^{2.60}$ varied widely from 4-10 Å.

c. Hydrogen bond contact frequency between $Y^{7.43}$-$Q^{2.60}$. Black dots are used to represent averages for each simulation replicate, and black bars give the standard error of the mean (s.e.m.) for each condition. G protein biased MP shows no contact between these residues, while arrestin-biased LFT shows hydrogen bonds between these two residues in most frames. **RO76** lies in the middle of these two values, with the hydrogen bond occurring about a third of the time. Significance was calculated using the Mann-Whitney U test (p values: * < 0.05, ** < 0.01).
